# A large quantitative analysis of written language challenges the idea that all languages are equally complex

**DOI:** 10.1038/s41598-023-42327-3

**Published:** 2023-09-16

**Authors:** Alexander Koplenig, Sascha Wolfer, Peter Meyer

**Affiliations:** Department of Lexical Studies, Leibniz Institute for the German Language (IDS), Mannheim, Germany

**Keywords:** Psychology, Human behaviour

## Abstract

One of the fundamental questions about human language is whether all languages are equally complex. Here, we approach this question from an information-theoretic perspective. We present a large scale quantitative cross-linguistic analysis of written language by training a language model on more than 6500 different documents as represented in 41 multilingual text collections consisting of ~ 3.5 billion words or ~ 9.0 billion characters and covering 2069 different languages that are spoken as a native language by more than 90% of the world population. We statistically infer the entropy of each language model as an index of what we call average prediction complexity. We compare complexity rankings across corpora and show that a language that tends to be more complex than another language in one corpus also tends to be more complex in another corpus. In addition, we show that speaker population size predicts entropy. We argue that both results constitute evidence against the equi-complexity hypothesis from an information-theoretic perspective.

Language is one of our most complex traits^1^. But how complex is it? And are all of the ~ 7000 distinct languages on earth equally complex—or not^2^? Quantifying the statistical structure and complexity of human language is essential to understanding a large variety of phenomena in linguistics, the study of human culture and natural language processing from language learning to language evolution and from the role of culture in shaping cognitive skills to the creation of artificial intelligence^1,3–10^.

The equi-complexity hypothesis, i.e. the idea of a principle of “invariance of language complexity”^2^, has been a longstanding and largely unquestioned assumption in modern linguistics^11–19^. In recent times, however, researchers have begun to challenge and scrutinize this “axiom”^20–22^. While nowadays there is more consensus that the complexity of languages (and language varieties) can vary both in different sub-domains of linguistic description and overall^21–23^, there has not been, to the best of our knowledge, a large scale quantitative evaluation of the equi-complexity hypothesis. Apart from collecting suitable test data, such an evaluation has to overcome the difficulty of measuring overall language complexity in the first place^22,24^: given any of different linguistic sub-domains for which there is a proposed complexity measure (e.g., measures of morphological, syntactic, referential complexity), it would not be reasonable to simply sum these measures. Therefore, it has been claimed that it is in fact impossible to measure overall complexity of a language^24^. Notwithstanding this difficulty, a test of the equi-complexity hypothesis is important both with respect to practical aspects of natural language processing^25^ and from a theoretical point of view^21^. For example, a roughly equal degree of overall complexity would point towards “some internal mechanism that stems from human communication patterns, or from the limitations of the human brain”^24^ and thus could help to further understand the cognitive/neural architecture of language^26^, for example by linking equality in language complexity to recently revealed general similarities in the way different languages are neurally processed^27^.

Here, we build on information theory, an area of mathematics that links probability and communication^28^ and provides notions of complexity that are both objective and theory-neutral^29^. To measure complexity, we use a statistical coding approach^30^ where the relevant conditional probability distributions are learned from written text data, so-called corpora^29,31,32^. To this end, the fuzzy notion of complexity as some kind of vector of separate values each measuring complexity in different linguistic sub-domains^28^ is replaced by a measure that is related to predictability – the better the next symbol in a sequence from a language can be predicted, the lower the complexity of this language. Providing a blueprint for the quantitative study of the statistical character of language, Shannon^33,34^ showed that prediction, probability and understanding are intimately related^35,36^ by demonstrating that humans are very good (in fact until very recently much better than any machine^37,38^) in predicting subsequent linguistic material based on previous input. Since Shannon’s seminal work, numerous studies have revealed that adults, children and even infants show an extraordinary ability to (unconsciously) exploit statistical information on different levels in the input they receive in order to efficiently predict/process linguistic material^39–41^. One of the key quantities in information theory is the average per-symbol information content or entropy rate* h*^28^: since, due to grammatical, phonological, lexical and other regularities governing language use, not every sequence of symbols is allowed^42^, *h* both (i) measures how much choice a writer has when selecting successive symbols and (ii) quantifies the reader’s uncertainty when predicting upcoming symbols^33^. In what follows, we show that *h* can also be interpreted as a complexity metric^43^: the harder it is, on average, to predict upcoming text—i.e. the higher the value of* h*—the greater is the complexity of the text as a whole^42,44–46^. Here, we argue that *h* can thus also be used to compare the complexity of different languages.

## Estimating entropy as a measure of average prediction complexity

Following Ref.^47^, we represent a text *κ* as a random variable that is created by drawing (with replacement) from a set of symbol types $\mathcal{V} =\{{s}_{1},{s}_{2},{s}_{3},\dots ,{s}_{V}\}$, where *V* is the number of distinct symbol types, i.e. $V=|\mathcal{V}|$. Depending on the chosen level of analysis, symbol types are taken to be either (Unicode) characters or word types. Correspondingly, a symbol token is any reoccurrence of a symbol type^47^. We can then count how often each symbol appears in *κ* and call the resulting frequency ${f}_{j}$, and can then represent *κ* as a distribution of symbol frequencies. In order to quantify the amount of information contained in *κ*, we can calculate the Gibbs-Shannon entropy *H* of this distribution as^28^:

1 $$H\left(\kappa \right)=-\sum_{j=1}^{V}p\left({s}_{j}\right)\cdot \mathrm{log}p\left({s}_{j}\right),$$

where $p\left({s}_{j}\right)=\frac{{f}_{j}}{\sum_{j=1}^{V}{f}_{j}}$ is the maximum likelihood estimator of the probability of ${s}_{j}$ in *κ* consisting of $\sum_{j=1}^{V}{f}_{j}$ tokens*.* In what follows, all logs are to the base two, so the quantities are expressed in bits. *H(κ)* can be interpreted as the average number of (yes/no) guesses that are needed to correctly predict the type of a symbol token that is randomly sampled from *κ*.

The entropy rate or per-symbol entropy of a stochastic process can be formally defined as^28,47^:

2 $$h\left(\kappa \right)=\underset{N\to \infty }{\mathrm{lim}}\frac{1}{N}{H}_{N}\left(\kappa \right)=\underset{N\to \infty }{\mathrm{lim}}\frac{1}{N}H\left({t}_{1}^{N}\right),$$

where ${t}_{1}^{N}= {t}_{1}, {t}_{2},\dots ,{t}_{N}$ represents a block of consecutive tokens of length *N* and ${H}_{N}\left(\kappa \right)$ denotes the so-called block entropy of block size *N*^31,47^.

Following Ref.^43^, we define ${F}_{N}$ as the *prediction complexity* of ${t}_{N}$ given ${t}_{1}, {t}_{2},\dots ,{t}_{N-1}$ as follows:

3 $${F}_{N}\equiv H\left({t}_{N}|{t}_{1}^{N-1}\right).$$

${F}_{N}$ quantifies the uncertainty of the *N*th symbol, given all preceding tokens ${t}_{1}^{N-1}$. Assuming a stationary stochastic process^28,47^, ${F}_{N}$ reaches the entropy rate *h* as *N* tends to infinity^28,43^:

4 $$h\left(\kappa \right)=\underset{N\to \infty }{\mathrm{lim}}{F}_{N}.$$

In analogy to *H(κ)*, the entropy rate $h(\kappa )$ can be informally understood as the average number of guesses that are needed to guess the next symbol of a sequence and thus incorporating the notion that prediction and understanding are intimately related^7,31^. Information can then be defined as any kind of knowledge that, when in your possession, allows you to make predictions with greater accuracy than mere chance^48,49^. Thus, *h* encompasses complexity from various linguistic sub-domains, since any form of linguistic (e.g. grammatical, phonological, lexical, pragmatic) or non-linguistic (e.g. world) knowledge^42^ will help a reader or listener to predict more accurately and will therefore reduce *h*.

To estimate $h(\kappa )$ in an experimental setting, a guessing method can be used. Here, human subjects are repeatedly presented with *N*—1 tokens of a text and are then asked to guess the symbol type ${s}_{j}$ of ${t}_{N}$. It can be shown^28,34,48,50^ that the minimum number of guesses needed to correctly predict symbol type ${s}_{j}$ is directly related to the conditional probability $p\left({t}_{N}={s}_{j}|{t}_{1}^{N-1}\right)$ of *j* given the corresponding context as $-\mathrm{log}p\left({t}_{N}={s}_{j}|{t}_{1}^{N-1}\right)$. Thus, assuming that the subject always follows an optimal guessing strategy^48^, an estimate of the average prediction complexity $h(\kappa )$ of a text *κ* can be computed by taking the average of the logarithms.

${F}_{N}$ is one of the central properties of surprisal theory^51,52^. This theory suggests that language processing involves generating and updating predictions about upcoming words or linguistic structures based on the context. Following from that, the surprisal theory states that the processing difficulty in incremental language comprehension (measured, for example, via reading times or ERP magnitudes) is a function of ${F}_{N}$. The effect of surprisal (or self-information) can be shown for a variety of psycholinguistic phenomena like word or construction frequency effects, syntactic garden paths, and anti-locality effects^29^. This shows how information-theoretic measures can be used to predict the relative processing complexity of sequential linguistic data, for example in psycholinguistic experiments.

Against this background, parallel corpora offer an intriguing source of data because they can be considered translational equivalents^53^: parallel texts are basically texts in different languages containing the same message. Therefore, potential differences in prediction complexity cannot be attributed to differences in content, style or register^25,54^ (for a discussion of potentials confounds see Refs.^53–58^). We now call *κ* a parallel corpus that consists of individual texts ${\kappa }_{i}$, where *i* denotes 1,…, *I* different languages. To test the question of whether all languages are equally complex, one could propose an experiment utilizing human subjects with *i* = 1,…, *I* different different native languages. In such an experiment, each participant would be presented with an individual text ${\kappa }_{i}$, in order to compute $h({\kappa }_{i})$ for each participant/language. The resulting variable $h\left(\kappa \right)$ maps each individual text ${\kappa }_{i}$ to its computed entropy rate, $h\left({\kappa }_{i}\right)$. According to the equi-complexity hypothesis, the variance of $h\left(\kappa \right)$, $Var[h(\kappa )]$, should not be significantly different from zero. However, the validity of such a conclusion ultimately rests on a ceteris paribus assumption: do all participants share the same level of individual language proficiency, is the selected text representative for the *I* different languages or are there potential language-specific characteristics, or cultural and contextual factors that could bias the results? Thus, the fact that $Var[h(\kappa )] >0$ alone would not constitute strong evidence against the equi-complexity hypothesis. Instead, the experiment could be repeated with different experimental subjects and a different parallel corpus $\iota$ that is compared with parallel corpus *κ*, by estimating both $h({\kappa }_{i})$ and $h({\iota }_{i})$ for *i* = 1,…, *I*. If the equi-complexity hypothesis holds true, we could rank the values of both $h(\kappa )$ and $h(\iota )$. We could then correlate the resulting complexity rankings $R(\kappa )$ and $R(\iota )$, i.e. we could compute the Spearman correlation coefficient $\rho [h\left(\kappa \right),h(\iota )]$. It would constitute evidence in favour of the equi-complexity hypothesis if $\rho [h\left(\kappa \right),h(\iota )]$ were not significantly greater than zero. Extending this line of reasoning to a scenario where multiple different parallel corpora are being considered, we should expect that the expected or average value of $\rho [h(\kappa ),h(\iota )]$, $E\{\rho [h(\kappa ),h(\iota )]\}$, computed based on all parallel corpus pairs ($\kappa$*,*$\iota$) where $\kappa \ne \iota$, should be close to zero if the equi-complexity hypothesis holds true. On the other hand, it would constitute evidence against the equi-complexity hypothesis if $E\{\rho [h(\kappa ),h(\iota )]\}\gg 0$.

However, employing a guessing method in a multi-text and multi-language scenario as outlined above would be highly impractical. As argued by Kolmogorov (as characterized in Ref.^48^), for a precise estimation of $h(\kappa )$, given that the preceding *N*—1 symbols are known, guessing subjects would effectively need to accurately specify the conditional probability for any potential symbol type. That is, at any point *N* in the text, subjects would need to accurately indicate $p\left({t}_{N}={s}_{j}|{t}_{1}^{N-1}\right)$ for any symbol type ${s}_{j}\in \mathcal{V}$. As reported by Ref.^43^, even in a comparatively simple scenario, where subjects were asked to specify probability distributions for 27 different characters (A-Z and space), the majority of the subjects aborted the experiment. While this problem could potentially be circumvented by greatly increasing the number of human subjects^43,48^, the resulting experiment would be both very intricate and extraordinarily expensive. In addition, humans have a tendency to assign biased probabilities to rare events^59,60^. This tendency poses a significant challenge in the context at hand, as word frequency distributions typically comprise a large number of rare events^61^. An alternative to estimating $h(\kappa )$ that does not rely on human subjects is to use computational language models (LMs). In this context, cognitive scientists and computational linguists have pointed out that LMs, most notably exemplified by the widespread success of OpenAI's ChatGPT chatbot, provide a computational working model for empirically studying various aspects of human language^62–64^. Note that training such models aims to minimize ${F}_{N}$ by generating the most accurate and probable next symbol based on the context provided by the preceding symbols. This means that during training, the model forms probabilistic expectations about the next symbol in a text and uses the true next symbol as an error signal to update the model's latent parameters^63^. The concept of using a text prediction criterion to uncover fundamental aspects of the underlying linguistic system can be traced back to the pioneering work of Elman^65^, who demonstrated that by employing this criterion, language models are capable of uncovering crucial elements within the language structure, thereby contributing to our understanding of language processing and generation^63^. Modern LMs greatly expand upon the foundations laid by Elman in their ability to predict text and reveal deeper insights into the underlying linguistic system^62^. In addition and again illustrated by ChatGPT, training modern LMs to learn to predict upcoming linguistic material allows the LM to produce language that is to a great extent indistinguishable from the language it has been trained on^42,63^.

**Figure 1:**
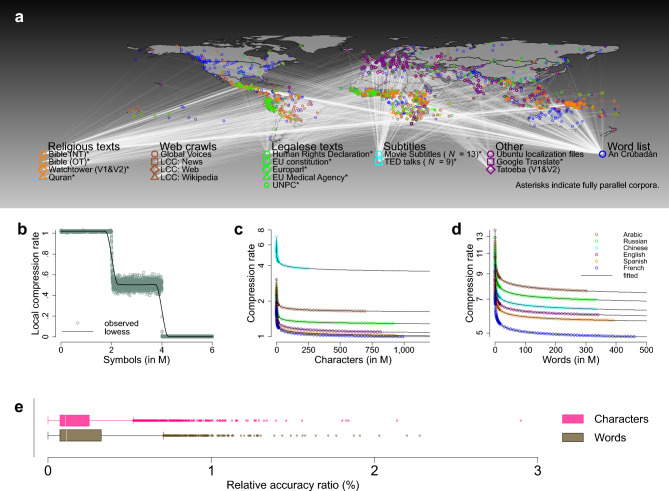
Dataset and entropy estimation. (**a**) Collected corpora and their geographical distribution. Asterisks indicate fully parallel corpora (see Supplementary Information: “Corpora” for details). (**b**) Illustration of PPM: we generated a synthetic string with 6 million (M) symbols from a source emitting two different symbol types whose statistical characteristics change every 2 M symbols: symbol types are randomly emitted for the first 2 M (expected *h* = 1.000), the second 2 M symbols are generated by a Hidden Markov sequence (expected *h* = 0.469), the third 2 M are generated by a pseudorandom sequence with mixed long-term dependencies (expected *h* = 0.000); emerald circles represent the local compression rate defined as the number of bits that PPM needs to compress the last 1000 symbols divided by 1000; the solid line represents a locally weighted scatterplot smoother (“lowess”). As can be seen, PPM successfully detects each pattern (see Methods: “Synthetic dataset” for details). ((**c**) and (**d**)) Compression rates (hollow circles) as a function of length in symbols (characters/words) for the six languages of the UNPC illustrate that without prior knowledge of the source, PPM learns to predict by acquiring a representation of the probabilistic structure of each language in one single pass. Solid lines represent our three parameter ansatz to estimate the asymptotic value of *h*. *NB*: For illustration purposes, only a selection of data points is shown for each language. (**e**) The distribution of relative accuracy ratios indicates that the curve can be accurately modelled by our ansatz. Box-plot elements throughout this paper are defined as follows: center line, median; box limits, first and third quartiles; whiskers as defined by Tukey^75^; points, outliers.

To estimate $h(\kappa )$ from corpus data, we make use of the fact that machine learning of natural languages can be seen as equivalent to text compression^42,66,67^. In particular, we focus on the wide class of compressors that consist of an LM and an entropy coder: the LM generates predicted probabilities $p\left({t}_{N}={s}_{j}|{t}_{1}^{N-1}\right)$ for the upcoming symbols given the past that are then taken by the entropy coder to perform compression by coding symbols with code length $-\mathrm{log}p\left({t}_{N}={s}_{j}|{t}_{1}^{N-1}\right)$^7,68^. Leveraging available corpora and multilingual text collections^69–72^, we compiled a database of parallel texts comprising a large variety of different text types, e.g. religious texts, legalese texts, subtitles for various movies and talks, and machine translations. Where necessary (and possible), we developed computational routines that made sure that the resulting corpora are as parallel as possible (see Methods: “Corpora” and Supplementary Fig. 1 for a quantitative description of the database). In addition, we added comparable corpora, i.e. texts that are not parallel but come from comparable sources and are therefore similar in content, again comprising very different text types/genres, e.g. newspaper texts, web crawls, Wikipedia articles, Ubuntu localization files, or translated example sentences from a free collaborative online database. Furthermore, we calculated Gibbs-Shannon unigram entropies *H* (Eq. (1)) based on word frequency information from the Crúbadán project^73^ that aims at creating text corpora for a large number of (especially under-resourced) languages. In total, we analysed 41 different multilingual corpora (Fig. 1a) consisting of 6513 documents or ~ 3.5 billion words/ ~ 9.0 billion characters covering 2069 different languages which are spoken as a native language by more than 90% of the world population and constitute ~ 46% of all languages that have a standardized written representation (see Supplementary Information: “Coverage”).

We use Prediction by partial matching (PPM), a computational LM originally developed for data compression^30,74^ to calculate the compression rate *r* (compressed size divided by message length *L* in symbols) as an index of the average prediction complexity for both words and characters as information encoding units^47^. Note that *r* is directly related to the quantity *perplexity* that is often used in natural language processing to measure the quality of a language model, where perplexity is defined as two to the power of *r*^29^. In order to compress, PPM, a variable-order Markov model, uses a set of up to 32 previous symbols as context to predict the most probable next symbol and thus effectively assigns a probability $p\left({t}_{N}={s}_{j}|{t}_{1}^{N-1}\right)$ for any symbol type ${s}_{j}\in \mathcal{V}$ based on the text it has already observed, which is exactly the information that is infeasible to get from human participants, as we have described above.

In Fig. 1b, we illustrate that PPM is a dynamic and adaptive method: every time the algorithm encounters new data, it updates its language model and is thus able to detect changing statistical characteristics of the source. With growing input, it gets better in predicting subsequent data^67^ (see Supplementary Table 1 for an interactive illustration), or put differently, PPM learns to exploit the statistical structure of the input, paralleling human language learning^41,42^ with interesting applications in natural language processing^76,77^, language production^78,79^ and—more generally—machine learning of patterns to predict (into) the future^7,79,80^. Figure 1c,d visualize this online learning behavior for the United Nations Parallel Corpus (UNPC, see Supplementary Information: “Corpora” for details)^81^ consisting of various documents in the six official languages of the United Nations. Importantly, *h* determines how hard it is to make accurate predictions once the statistical structure of the input language has been learned^31,32^. Therefore, when estimating *h* via compression, it is essential to take into account that the algorithm needs (a certain amount of) training to learn how to exploit the statistical structure of the input in order to make accurate predictions. This is especially relevant for natural languages where the convergence to the underlying source entropy is known to be notoriously slow, because of long-range correlations^82,83^ and due to the fact that a probabilistic model of language remains unknown^31,32^. We therefore calculate *r* for subsets of increasing length (coloured circles in Fig. 1c,d) and fit a nonlinear extrapolation function given by an ansatz^31,32^ to estimate the asymptotic value of *h* (solid lines in Fig. 1c,d; see Methods: “Estimating entropy” and Supplementary Figs. 2, 3 for details on extrapolation and statistical estimation).

Figure 1c,d visually indicate that our ansatz fits the observed curves very well (solid lines; see Supplementary Figs. 4–21 for all corpora, also see Supplementary Information: “Ansatz functions” and Supplementary Tables 2–4 for details). Figure 1e confirms this impression: 99% of the ~ 5000 compression series show an accuracy ratio, i.e. an approximate average percentage difference, between (held-out) observed and predicted values that is within 1% (see Eq. (8)).

One might want to object that *h* strongly depends on the writing system. For example, Fig. 1c demonstrates that it is indeed considerably more difficult to predict Chinese characters (*h* = 3.03 bits per symbol (bps)), obviously due to the fact that written Mandarin Chinese employs a logographic system where individual characters typically represent words/morphemes compared to the other five languages that employ alphabetic systems where symbols typically represent phonemes (here *h* $\in$ [0.89, 1.51] bps) which affects the capacity of the communication channel^33,58^. However, on the word level (Fig. 1d), Chinese (*h* = 5.51 bps) occupies a middle ground (*h* $\in$ [4.27, 6.25] bps). In general, variability in both *h* and *r* tends to be smaller for words (median relative standard deviation *SD*_*med*_ = 15.25% for *h* and *SD*_*med*_ = 11.60% for *r*) than for characters (*SD*_*med*_ = 32.03% for *h* and *SD*_*med*_ = 28.13% for *r*; see Supplementary Fig. 22). In what follows, we will take potential influences of the writing system into account by using writing system as a covariate and by replicating analyses specifically for documents that use the most widely adopted writing system, Latin script (~ 80% of all our documents).

## Results

**Figure 2:**
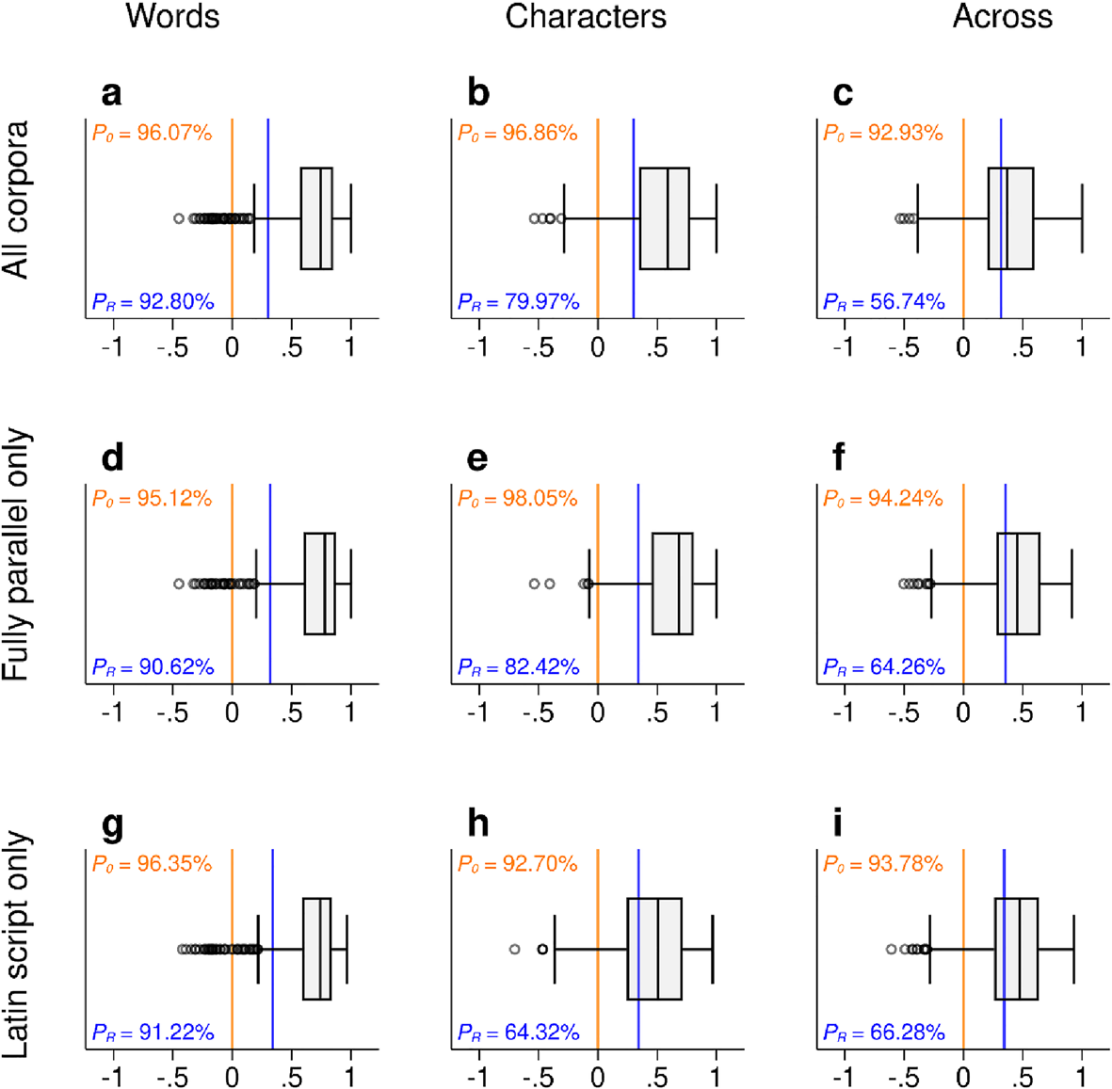
Testing the similarity of prediction complexity rankings. Distribution of pairwise Spearman correlations for the measure of prediction complexity *h* between all corpus pairs for all corpora (**a**–**c**), fully parallel corpora (**d**–**f**) and documents that use Latin script (**g**–**i**). (**a**,**d**,**f**) display the results for words as information encoding units, while (**b**,**e**,**h**) depict the results for characters as information encoding units. (**c**,**f**,**i**) visualize the cross-symbolic level results, showing the correlation distribution between words in one corpus and characters in another corpus. The value presented in the top-left corner represents *Ρ*_*0*_, indicating the percentage of *ρ*-coefficients that are above zero as shown by the orange line. The value in the bottom-left corner represents *Ρ*_*R*_, representing the percentage of *ρ*-coefficients that surpass the random baseline as shown by the blue line.

As outlined above, we evaluated the similarity of prediction complexity rankings by computing Spearman correlation coefficients $\rho [h(\kappa ),h(\iota )]$ for all corpus pairs ($\kappa$*,* $\iota$) where $\kappa \ne \iota$. We restricted computations to all text pairs with at least five common languages (see Supplementary Information: “Correlation matrix” for details). On the level of words as information encoding units (total number of correlation coefficients*,* ${N}_{\rho }$ = 764), the mean correlation coefficient is *ρ*_mean_ = 0.67, the median correlation across corpora is *ρ*_med_ = 0.74 (first quartile *Q*_1_ = 0.57). The percentage of $\rho$-coefficients that are above zero is *Ρ*_*0*_ = 96.07%. To put this into perspective, just by chance we would expect ~ 50% of all $\rho$-coefficients to be above zero. To further evaluate the statistical significance of this result, we randomly re-arranged the values of $h(\kappa )$ in each corpus pair to obtain ${h}{^\prime}(\kappa )$ and computed Spearman correlation coefficients between ${h}{^\prime}(\kappa )$ and $h(\iota )$, ${\rho }{^\prime}=\rho [{h}{^\prime}(\kappa ),h(\iota )]$. As a random baseline, we computed the 9th decile for the set of all permuted ${\rho }{^\prime}$-values. This means that 90% of all ${N}_{\rho {^\prime}}$ = 764 ${\rho }{^\prime}$-values show a value that is lower than or equal to this random baseline. The percentage of unpermuted $\rho$-coefficients that are above this baseline is *Ρ*_*R*_ = 92.80%. Putting this into perspective again, just by chance, we would expect *Ρ*_*R*_ to be ~ 10%. Figure 2a visualizes this result. A similar pattern arises if we measure *h* on the character level (${N}_{\rho }$ = 764; *ρ*_mean_ = 0.55; *ρ*_med_ = 0.59; *Q*_1_ = 0.35; *Ρ*_*0*_ = 96.86%; *Ρ*_*R*_ = 79.97%; see Fig. 2b). Even if we compare entropy rates *across* corpora and *across* symbolic levels, i.e. we correlate the distribution calculated for words in one corpus with the distribution calculated for characters in another corpus, there tends to be a positive statistical association (${N}_{\rho }$ = 1528; *ρ*_mean_ = 0.38; *ρ*_med_ = 0.37; *Q*_1_ = 0.20; *Ρ*_*0*_ = 92.94%; *Ρ*_*R*_ = 56.74%; see Fig. 2c). Analogous patterns emerge if the computations are restricted to fully parallel corpora (for words: ${N}_{\rho }$ = 512; *ρ*_mean_ = 0.69; *ρ*_med_ = 0.78; *Q*_1_ = 0.60; *Ρ*_*0*_ = 95.12%; *Ρ*_*R*_ = 90.62%; see Fig. 2d; for characters: ${N}_{\rho }$ = 512; *ρ*_mean_ = 0.61; *ρ*_med_ = 0.68; *Q*_1_ = 0.45; *Ρ*_*0*_ = 98.05%; *Ρ*_*R*_ = 82.42%; see Fig. 2e; across symbolic levels: ${N}_{\rho }$ = 1024; *ρ*_mean_ = 0.44; *ρ*_med_ = 0.46; *Q*_1_ = 0.28; *Ρ*_*0*_ = 94.24%; *Ρ*_*R*_ = 64.26%; see Fig. 2f). Analogous patterns also emerge if the computations are restricted to documents that use Latin script, in order to rule out the possibility that these results are mainly driven by the fact that different languages use different writing systems (for words: ${N}_{\rho }$ = 740; *ρ*_mean_ = 0.67; *ρ*_med_ = 0.74; *Q*_1_ = 0.59; *Ρ*_*0*_ = 96.35%; *Ρ*_*R*_ = 91.22%; see Fig. 2g; for characters: ${N}_{\rho }$ = 740; *ρ*_mean_ = 0.46; *ρ*_med_ = 0.50; *Q*_1_ = 0.24; *Ρ*_*0*_ = 92.70%; *Ρ*_*R*_ = 64.32%; see Fig. 2h; across symbolic levels: ${N}_{\rho }$ = 1480; *ρ*_mean_ = 0.43; *ρ*_med_ = 0.48; *Q*_1_ = 0.26; *Ρ*_*0*_ = 93.78%; *Ρ*_*R*_ = 66.28%; see Fig. 2i).

Supplementary Table 6 shows that similar results are obtained if we use *r* or *H* instead of *h* to evaluate the similarity of complexity rankings between different corpora. In addition, Supplementary Table 6 also contains results where we adjust our prediction complexity estimates for the potential influence of the text length to rule out the possibility that our results are mere artefacts resulting from the fact that most, if not all, quantities in the context of word frequency distributions vary systematically with the text length^61,84,85^.

We proceed by validating the above results against data from the Crúbadán project^73^. Based on word frequency information for 1943 different languages, we calculated Gibbs-Shannon unigram entropies (Eq. (1)) to generate a variable *H*_*Crúbadán*_. We then computed $\rho [h(\kappa ),{H}_{Cr\acute{u} bad\acute{a} n}]$ for each $\kappa$ among our 40 corpora. Results are very similar to the ones presented above if *H*_*Crúbadán*_ is correlated with *h* on the level of words, ${N}_{\rho }$ = 40; *ρ*_mean_ = 0.54; *ρ*_med_ = 0.51; *Q*_1_ = 0.42; *Ρ*_*0*_ = 97.50%; *Ρ*_*R*_ = 95.00%. Results are comparable but less pronounced if *H*_*Crubadan*_ is correlated with *h* on the level of characters, ${N}_{\rho }$ = 40; *ρ*_mean_ = 0.36; *ρ*_med_ = 0.30; *Q*_1_ = 0.20; *Ρ*_*0*_ = 97.50%; *Ρ*_*R*_ = 45.00%. These results indicate that it is appropriate to use *H*_*Crúbadán*_ as an additional index of prediction complexity in what follows.

To test if the similarity of prediction complexity rankings mainly results from the phylogenetic and geographical relatedness of languages^86–88^, we tested how well the distribution of *h* in one corpus can be predicted using its distribution in another corpus. To this end, we ran linear mixed effects models (LMM) where random intercepts for language family, macro-area and country are included to account for the genealogical and geographic relatedness of languages for all corpora. Again, we restricted the computations to all corpus pairs ($\kappa$*,* $\iota$) with at least five common languages for which genealogical and geographic information was available. We then computed a correlation coefficient, *ρ*^*LMM*^, that is based on a measure of explained variance by the fixed effects of the LMM^89^ and proceeded as above (see Methods: “Evaluating the similarity of complexity rankings” for details). We find that the results remain stable (for words: ${N}_{{\rho }^{LMM}}$ = 1482; ${\rho }_{mean}^{LMM}$ = 0.60; ${\rho }_{med}^{LMM}$ = 0.66; *Q*_1_ = 0.45; *Ρ*_*0*_ = 95.95%; *Ρ*_*R*_ = 91.09%; for characters: ${N}_{{\rho }^{LMM}}$ = 1,482; ${\rho }_{mean}^{LMM}$ = 0.52; ${\rho }_{med}^{LMM}$ = 0.56; *Q*_1_ = 0.30; *Ρ*_*0*_ = 96.90%; *Ρ*_*R*_ = 81.98%; across symbolic levels: ${N}_{{\rho }^{LMM}}$ = 3042; ${\rho }_{mean}^{LMM}$ = 0.30; ${\rho }_{med}^{LMM}$ = 0.27; *Q*_1_ = 0.13; *Ρ*_*0*_ = 91.29%; *Ρ*_*R*_ = 61.14%). Similarly, we can use *h* to predict *H*_*Crúbadán*_ (for *h* measured on the word level: ${N}_{{\rho }^{LMM}}$ = 39; ${\rho }_{mean}^{LMM}$ = 0.36; ${\rho }_{med}^{LMM}$ = 0.35; *Q*_1_ = 0.19; *Ρ*_*0*_ = 97.44%; *Ρ*_*R*_ = 82.05%; for *h* measured on the characters level: ${N}_{{\rho }^{LMM}}$ = 39; ${\rho }_{mean}^{LMM}$ = 0.23; ${\rho }_{med}^{LMM}$ = 0.14; *Q*_1_ = 0.06; *Ρ*_*0*_ = 94.87%; *Ρ*_*R*_ = 41.03%). Supplementary Table 6 shows that similar results are obtained if we use *r* or *H* instead of *h*. This table also contains results where computations are restricted to fully parallel corpora or Latin script and shows that results remain stable if the potential influence of the text length is controlled for.

If the equi-complexity hypothesis holds true, there cannot be any association between prediction complexity and language external factors. However, in what follows we will demonstrate that the estimated number of language speakers, as proxy for population structure^90^, predicts prediction complexity. To account for the potential non-independence of data-points described above, we ran separate LMMs by considering a set of models, which corresponds to all possible combinations of inclusions and exclusions of the following covariates: (i) (crossed) random intercepts for the following groups: corpus, language family, language, macro-area, country and writing script; (ii) random slopes (i.e. we allow the effect of population size to vary across different groups) for all groups except language (since speaker population size does not vary within languages) and (iii) a potential fixed effect for the (log of) speaker population size (see Methods: “Differences across populations” for details). As a means of selecting between models, we use Akaike’s information criterion AIC^91^ where lower values indicate a more apt model. On both symbolic levels, we fitted a total of 550 different models. On the word level, the model with the lowest AIC includes random intercepts for corpus, language family, language, macro-area, country and writing script and random slopes for corpus, language family and country. The estimated effect of speaker population size, *β*_LMER_ = 0.054 (standard error *s.e.* = 0.013; number of cases *N* = 3853) is significant at *p* < 0.001. To further test whether the inclusion of the fixed effect for speaker population size is warranted, we selected the best LMM that does not include a fixed effect or random slopes for speaker population size and calculated ΔAIC, i.e. the difference between the AIC-value for the model without the inclusion of speaker population size and the full model from above. Only if this value is positive, the inclusion of the speaker population size seems appropriate and the greater the value of ΔAIC, the greater the support for the full model. Here a value of ΔAIC = 30.71 strongly supports the inclusion of speaker population size in the model^92^. On the character level, the best model includes random intercepts for corpus, language family, language, macro-area, country and writing script and random slopes for corpus, language family and writing script. The coefficient for *β*_LMER_ = 0.058 (*s.e.* = 0.027; *N* = 3853) is significant at *p* < 0.05. A value of ΔAIC = 42.56 again supports the inclusion of speaker population size. We then fit additional models for *H*_*Crúbadán*_ as the outcome. Here, models do not contain a random effect for corpus, but LMMs additionally contain fixed effects for text length, available number of documents (both logged) and a binary variable indicating whether the word frequency list is truncated to account for differences in the way different Crúbadán word lists were generated (see Supplementary information: S3.8 for details). In total, we fit 194 different models. The best model includes random intercepts for language family, language, macro-area, country and writing script and random slopes for language family, macro-area, country and writing script. The coefficient for *β*_LMER_ = 0.160 (*s.e.* = 0.029; *N* = 1914) is significant at *p* < 0.001. A value of ΔAIC = 130.65 again supports the inclusion of speaker population size into the model. In sum, these results indicate that languages with more speakers tend to have a higher prediction complexity which in turn questions the idea that all languages are equally complex. Additional results for *r* and *H* as outcome are presented in Supplementary Table 7. This table also contains results where computations are restricted to fully parallel corpora and shows that results remain stable if the potential influence of the text length is controlled for.

Finally, we want to discuss and examine several potential limitations and extensions of our study.

First, we tested if the obtained similarity of language complexity rankings between different corpora can mainly be attributed to the degree that different languages make use of inflectional morphology^25^. We used the Treetagger^93^ with a corresponding language-specific parameter file to lemmatize 16 translations of the European constitution (EUconst, see Supplementary Information: “Corpora”) prior to estimation of *h* on both symbolic levels to remove the effect of inflectional morphology^25^ and to generate a variable $h({\mathrm{EUconst}}^{lemma})$. We then computed $\rho [h(\kappa ),h({\mathrm{EUconst}}^{lemma})]$ for all 38 corpora that share at least five languages with EUconst. Results are stable for lemmatized texts on both symbolic levels, words: ${N}_{\rho }$ = 38; *ρ*_mean_ = 0.57; *ρ*_med_ = 0.56; *Q*_1_ = 0.42; *Ρ*_*0*_ = 100.00%; *Ρ*_*R*_ = 71.05%; characters: ${N}_{\rho }$ = 38; *ρ*_mean_ = 0.55; *ρ*_med_ = 0.59; *Q*_1_ = 0.46; *Ρ*_*0*_ = 97.37%; *Ρ*_*R*_ = 76.32%. Hence, our overall results still stand, even if we control for differences in inflectional morphology.

Secondly, due to the large amount of textual data, we used an off-the-shelf compressor that is optimized for speed and memory usage^7,32^, 7-zip PPMd^94^. While PPM consistently performs well on text compression benchmarks^7,79^, its language model is rather simple. To rule out the possibility that more complex/larger language models would lead to different results, we compressed the parallel BibleOT corpus (see Supplementary Information: “Corpora”) again, but used a much more sophisticated algorithm called CMIX. Compared to PPMd, CMIX uses an ensemble of several thousand of independent prediction models that are combined using different deep neural network architectures^79,95,96^ and some of the contexts used for prediction are allowed to be non-contiguous in order to capture longer-term dependencies^79^. CMIX achieves state-of-the-art compression rates at the expense of much higher CPU/memory usage. In addition, CMIX is slower than PPMd by several orders of magnitude (Supplementary Table 8 shows that it takes CMIX on average ~ 4724 times longer to compress than PPMd). Instead of using an extrapolation approach, we therefore only used CMIX to compute compression rates $r({\mathrm{BibleOT}}_{CMIX})$. We then computed $\rho [h(\kappa ),r({\mathrm{BibleOT}}_{CMIX})]$ for all 38 corpora that share at least five languages with BibleOT. Again, the results support the overall patterns, with ${N}_{\rho }$ = 38; *ρ*_mean_ = 0.80; *ρ*_med_ = 0.84; *Q*_1_ = 0.75; *Ρ*_*0*_ = 97.37%; *Ρ*_*R*_ = 97.37% for words and ${N}_{\rho }$ = 38; *ρ*_mean_ = 0.60; *ρ*_med_ = 0.63; *Q*_1_ = 0.52; *Ρ*_*0*_ = 100.00%; *Ρ*_*R*_ = 97.37% for characters. In conclusion, there is no indication that the type of compressing algorithm influences our results.

Thirdly, modern language models are often not trained on either the level of words or the level of characters, but on the sub-word level^63^. To test this, we used the BibleOT corpus again and tokenized each text into sub-word units by byte pair encoding (BPE)^54,97^ which plays an important role in many state-of-the-art natural language model applications^98,99^ and provides strong baseline results on a multilingual corpus^100^. Applying BPE results in a sequence of sub-word units, e.g. “|he |may |give |me |a |kin|dly |re|cep|tion |”*.* We compressed each such sequence with CMIX and computed $r({\mathrm{BibleOT}}_{CMIX}^{BPE})$; see Methods: “Analyses using CMIX” for details. We then computed $\rho \left[h(\kappa ),r\left({\mathrm{BibleOT}}_{CMIX}^{BPE}\right)\right]$. Results indicate that a CMIX BPE model also supports our results, with ${N}_{\rho }$ = 38; *ρ*_mean_ = 0.67; *ρ*_med_ = 0.67; *Q*_1_ = 0.62; *Ρ*_*0*_ = 97.37%; *Ρ*_*R*_ = 97.37% for words and ${N}_{\rho }$ = 38; *ρ*_mean_ = 0.57; *ρ*_med_ = 0.59; *Q*_1_ = 0.50; *Ρ*_*0*_ = 100.00%; *Ρ*_*R*_ = 89.47% for characters. So, the level of analysis (words, characters, sub-word units) does not influence the results.

Fourthly, our study is confined to written language. To test a potential connection to spoken language, we use data from the VoxClamantis corpus^101^ that is derived from audio readings of the New Testament of the Bible. We prepared sequences of phonemes for 29 languages and compressed each such sequence with CMIX and computed $r\left({\mathrm{VoxClamantis}}_{CMIX}^{phoneme}\right)$ and computed Spearman correlations with $h(\kappa )$ based on the written corpora. On the word level, we find that there is only a comparatively weak association, with ${N}_{\rho }$ = 34; *ρ*_mean_ = 0.18; *ρ*_med_ = 0.11; *Q*_1_ = –0.03. While only *Ρ*_*R*_ = 11.76% of all correlation coefficients are above the random baseline, *Ρ*_*0*_ = 73.53% are above zero. The statistical associations are much more pronounced on the character level with ${N}_{\rho }$ = 34; *ρ*_mean_ = 0.37; *ρ*_med_ = 0.43; *Q*_1_ = 0.14. Here, *Ρ*_*0*_ = 85.29% of all coefficients are above zero and *Ρ*_*R*_ = 23.53% are above the random baseline. To further test the equi-complexity hypothesis in this context, we ran a total of 27 LMMs with the (log of) $r\left({\mathrm{VoxClamantis}}_{CMIX}^{phoneme}\right)$ as the outcome and combinations of random intercepts and slopes for language family, macro-area and country (see Methods: “Analyses using CMIX” for details). The model with the lowest AIC includes random intercepts for language family, macro-area and country and random slopes for language family and country. As above, the coefficient for speaker population size is positive with *β*_LMER_ = 0.025 (*s.e.* = 0.011; *N* = 28) and is significant at *p* < 0.05. We believe it is important to emphasize that these results are preliminary in nature. The limited sample size, both in terms of available languages and sequence length, as well as the fact that the corpus consists entirely of Bible readings of varying quantity and quality per language, and other caveats^101^, make it premature to draw definitive conclusions regarding the support or lack thereof for our findings in written language. Nevertheless, we believe that the results show that further investigation of spoken language in this context would be an interesting and important avenue for future research.

## Discussion

A central goal of linguistics is to understand the diverse ways in which human language can be organized. In this paper, we present the results of a large cross-linguistic analysis of written language that we conducted to test the equi-complexity hypothesis which assumes that all languages are (in some sense) equally complex. We operationalized our key quantity of interest, prediction complexity $F$, in information-theoretic terms as the minimum number of guesses that are needed on average to correctly predict subsequent linguistic material based on the preceding context. All other things being equal, we defined a language *A* to be more complex than another language *B*, if *F*_*A*_ > *F*_*B*_. In the limit, *F* converges to the average per-symbol information content or entropy rate *h* that both measures how much choice a writer has when selecting successive symbols and quantifies the amount of uncertainty when predicting upcoming symbols. We argued that computational language models can be used to estimate *h* since training such models aims to minimize *F*. Based on this logic, we presented a method that can be used to statistically infer the asymptotic value of *h* based on computing compression rates for strings of increasing lengths. Equipped with this information-theoretic estimation framework, we compiled a database consisting of a total of 41 different multilingual parallel and comparable corpora comprising a large variety of different text types. In total, we estimated *h* for more than 6000 texts (Fig. 1). To test the equi-complexity hypothesis, we evaluated the similarity of prediction complexity rankings by computing Spearman correlation coefficients between $h(\kappa )$ and $h(\iota )$ for all corpus pairs ($\kappa$*,* $\iota$). We argued that it would constitute evidence in favour of the equi-complexity hypothesis if the mean value of $\rho [h(\kappa ),h(\iota )]$ would be close to zero. In a series of quantitative analyses, we showed that this is not the case. By thoroughly evaluating the similarity of prediction complexity rankings, we arrived at our main empirical finding: a language with high/low entropy rate in one corpus also tends to be more/less complex in another corpus (Fig. 2 and Supplementary Table 6). As an additional test of the equi-complexity hypothesis, we then examined whether the estimated number of speakers predicts prediction complexity. Controlling for the potential non-independence of data points due to the phylogenetic and geographical relatedness of languages in a mixed effects modeling approach, we showed that both parametric *p*-values and information-theoretic differences in AIC support the idea that speaker population size is a significant predictor of *h* (see Supplementary Table 7). We argued that this association between population structure and prediction complexity also questions the equi-complexity hypothesis, because languages with more speakers—on average—seem to be more complex.

The extent to which one finds our results convincing certainly depends on the extent to which one considers our information-theoretical measure to be a suitable proxy for the overall complexity of a language. Given the close link to the surprisal theory of language comprehension discussed above and the success of contemporary language models, we are cautiously optimistic. Nevertheless, we admit that it would be highly beneficial to find out how well our information-theoretic operationalization of complexity relates to more traditional notions of language complexity. In the absence of a clear benchmark for evaluating this, a potential fruitful starting point would be to use Grambank, a recently published global database of grammatical features of unprecedented size^102^ and test, for example, if grammatical complexity in the sense of fusion and informativity as specified by Ref.^103^ predicts our measure of prediction complexity.

On the other hand, caution is needed when trying to compare a traditional linguistic notion of complexity with the measure of predictive complexity we use. To give an example from phonology, a language with a canonical syllable pattern of CV would typically be considered to have a simpler syllable structure than a language with a (C)(C)CV(C) pattern^104^. This judgment arises naturally from the fact that a description of the permitted phoneme sequences is shorter for the CV-type. To be more specific, we may compare a hypothetical CV language L1 with five consonants and five vowels with another hypothetical (C)(C)CV(C) language L2 featuring the same phoneme inventory. The syllable type inventory of L1 can be described by the regular expression [srptk][aeiou]. For L2, let us assume that it follows a typologically widespread pattern in that complex onsets are restricted to certain clusters, in our case to /pr/, /kr/, /tr/, /ps/, /ks/, /ts/, /spr/, /str/, /skr/. The optional coda can only be one of /s/ or /r/. The regular expression for L2 syllables is obviously much longer: (s[ptk]r|[ptk][sr]|[srptk])[aeiou][sr]? Also, the average number of phonemes per syllable, often taken as a proxy for syllable complexity^105^, is necessarily higher for L2 than for L1. Nevertheless, mainly due to the restrictions in consonantal patterns, the prediction complexity of L2 will, all other things being equal, be slightly lower than that of L1 (see Methods: “Syllable patterns” for a computer simulation). If we modify our assumptions to the effect that in multi-syllable words of L2, one of the vowels is dominant in non-first syllables (perhaps because L2 has fixed first-syllable stress and vowels are typically reduced to schwa in unstressed syllables), while in L1 all vowels are equiprobable in all word positions, then L2 will even have a notably smaller entropy rate *h* than L1 (see Methods: “Syllable patterns” for a computer simulation). This simple picture becomes severely more complicated as soon as interference with other factors is taken into consideration, such as marked prevalence of certain phonemes in the most frequent word types or in frequent affixes.

One of the reasons for the observed discrepancy between more traditional complexity measures, such as an intuitive qualitative ‘description length’ or phoneme counts, and prediction complexity is that the latter, as a stochastic measure, is based on a much richer set of frequency-related data. Informally speaking, to calculate the entropy rate of a stochastic process, the probabilities of all possible sequences produced by that process have to be taken into account. It goes without saying that this does not invalidate the utility of any established criteria of structural complexity, which may play important roles in linguistic theories of domains such as phonology or morphology. As our miniature example suggests, each such criterion has a specific influence on the global measure of prediction complexity, albeit not always in a very straightforward or intuitive way.

Furthermore, it would be worthwhile to conduct a more comprehensive examination to determine if our findings extend beyond written language and are applicable to spoken language as well. The preliminary results we have presented, which are based on the VoxClamantis corpus^101^, can serve only as a starting point in that direction. Similarly, while we have demonstrated that our findings extend beyond a simple compression algorithm like PPM to a more complex one like CMIX, we believe that conducting a more comprehensive examination using large language models based on deep neural networks, such as transformers^106^, would also be an important avenue for future research.

Against this background, our study offers some points of departure for future studies. For example, we showed that languages with more speakers tend to have higher prediction complexity. At first sight, this result stands in contrast to the 'linguistic niche hypothesis' that argues that languages spoken in larger communities tend to be less complex^3,107–110^. However, note that our ansatz function has three parameters (see Eqs. (7), (8), (9)): the limiting entropy rate *h*, a proportionality constant and an exponent *b.* While *h* quantifies how difficult it is to predict, *b* quantifies how difficult it is to learn to predict, as aptly put by Ref.^32^: lower *b*-values are indicative of slower convergence, i.e. learning is more difficult (see Supplementary Fig. 3 for an illustration). In Supplementary Figs. 23 and 24 we show that there tends to be a positive statistical association between *b* and *h* that indicates that languages that are harder to predict tend to be easier/faster to learn for PPM. This indirectly implies that languages with more speakers should—on average—be easier to learn. Systematically analyzing this relationship could be the subject of a future paper.

In sum, our study highlights the potential of large-scale cross-linguistic analyses in enhancing our understanding of different phenomena within the domains of human languages, cognition, and culture.

## Methods

### Corpora

In total, we analysed 41 different multilingual corpora by compressing ~ 30.2* M* (sub-)strings of varying lengths. Details regarding all corpora used in this paper, data preparation and compression can be found in Supplementary Information: Corpora. In Supplementary Fig. 1, we visualize several important aspects of our database (also see Supplementary Table 4). Supplementary Fig. 1a shows that most corpora only consist of a few tens of texts (*N*_median_ = 40). For some corpora, the reason for this is rather simple, e.g. the European constitution was only translated into the languages of the European Union. However, for other corpora, e.g. the 13 subtitle corpora, translations into further languages are not available. On the other side of the spectrum, we have 11 corpora that consist of more than 100 different documents. Supplementary Fig. 1b complements this observation by showing that our database is also unbalanced at the language level: while we have more than 100 languages with at least 10 available data points, i.e. documents, we only have less than four available data points for most languages (~ 84%). This reflects the fact that especially for languages that are spoken only by a small number of people, there exists only a very limited number of documents that are electronically available^73^. Correspondingly, Supplementary Fig. 1c shows that our database is biased towards languages with more speakers. For example, while the estimate for the median number of speakers for all documented languages is 8000, the median number for which we have available data is 30000. Finally, Supplementary Fig. 1d shows that many documents are rather short, e.g. 25% of the documents are below 14575 characters or 3181 words. However, 200 documents are longer than 1 million characters, 49 documents are longer than 10 million characters and the longest documents are several hundred million words or more than a billion characters long. We adapt our analysis strategy accordingly by both using state-of-the-art statistical methods that allow for unbalanced datasets and by statistically comparing the diversity structure found in smaller corpora (i.e. corpora consisting of shorter documents and/or corpora with only a limited number of available documents) with the underlying structure found in bigger corpora (i.e. longer documents and/or available data points for many languages). Here, the idea is that if the results in both smaller and bigger corpora point in the same direction, then this strengthens the claim that those results are more than just an artefact resulting from an unbalanced database. In addition, we use both parametric and non-parametric methods to evaluate the results. Supplementary Fig. 2 visualizes how we adapt Shannon’s information-theoretic view of communication to analyse our database.

### Estimating entropy

To estimate $h(\kappa )$ computationally, we use a data compression algorithm, since the true probability distribution for natural language is unknown^7,31^. The algorithm generates a language model, i.e. an estimate of the probability distribution of *κ* that can then be used for encoding via arithmetic coding^7,32,111^. We use PPM as implemented in the 7-zip software package, which is based on Dmitry Shkarin’s PPMd^94^. The algorithm makes an assumption of the Markov property: To encode/predict the next symbol, the algorithm uses the last *o* symbols that immediately precede the symbol of interest. If the order *o* context has not been seen before, the algorithm attempts to make a prediction based on the last *o*-1 symbols. This is repeated until a match is found, or, if no match is found until order 0, then a fixed prediction is made. In general, let $N(\kappa )$ denote the size (in symbols) of text $\kappa$ and let $R(\kappa )$ denote the size (in bits) of the compressed text $\kappa$. For brevity, we write $h$, $R$ and $N$ instead of $h\left(\kappa \right)$, $R\left(\kappa \right)$ and $N\left(\kappa \right)$ in what follows.

Then the compression rate $r=R/N$ is an upper bound on the underlying entropy rate $h\left(\kappa \right)$, i.e.:

5 $$r\ge h.$$

Importantly, $h$ is defined in the limit, i.e. for a text whose length $N$ tends to infinity^28,47^. Given stationarity and ergodicity^28^, the following equality holds for universal compressors^32^:

6 $$\underset{N\to \infty }{\mathrm{lim}}r=h.$$

Or put differently, the entropy rate measures how difficult it is to predict subsequent text based on the preceding input when the optimal compression scheme is known^32^. Equation (6) implies that convergence to the source entropy is only guaranteed in the limit^112^, i.e. when the text size approaches infinity. One way to take into account the dependence on $N$ is to use extrapolation when estimating $h$ via compression^32^. However, the (probabilistic) relationship between (the convergence of) *h* and *N* is unknown. To estimate *h*, we use a variant of the following ansatz suggested by ref.^31^:

7 $${r}_{n}=h+A\cdot \frac{\mathrm{log}n}{{n}^{b}},$$

where *A* > 0, *b* > 0 and—assuming that the entropy rate is positive^32^—$h>0$; ${r}_{n}=\left({R}_{1}^{n}\right)/n$ denotes the number of bits per symbol that are needed to compress the first *n* symbols of *κ*. In general, the idea of the ansatz is to calculate the compression rate for different sub-sequences of *κ* of increasing length. This gives us a measure of how well language learning succeeds^67,113^. For example, we can feed the compressor with the first *n* = 1⋅*m* symbols and calculate the compression rate for this subsequence where *m* is some pre-defined chunk size, e.g. 1000 symbols. After that, the compression rate is calculated for the first *n* = 2⋅*m* symbols and the compression rate is calculated again. This procedure is repeated until the end of *κ* is reached. The resulting series of compression rates for texts that consist of $1, 2, ..., \left\lfloor \frac{N}{m} \right\rfloor$ chunks can then be used to fit the three parameters to the data. We fit the following nonlinear ansatz function by log-least squares:

8 $${r}_{n}=\mathrm{exp}\left({h}^{*}+\mathrm{exp}({A}{^\prime})\cdot \frac{\mathrm{log}n}{{n}^{\mathrm{exp}({b}{^\prime})}}\right)+\acute{\text{o}}_n,$$

where $\acute{\text{o}}_n$ is an independent and identically distributed (i.i.d.) error term and exp() denotes the exponential function (see Supplementary Information: "Ansatz functions" where we discuss other ansatz functions and different error specifications that have been suggested in the literature^31,32,114^ and justify our choice). Since we want *A* and *b* to be positive, we set interval constraints that make sure that the optimization algorithm will not search in the negative subspace by fitting both parameters as exponentials, i.e. we estimate ${A}{^\prime}=\mathrm{log}(A)$ and ${b}{^\prime}=\mathrm{log}\left(b\right)$. The limiting entropy rate of Eq. (7) can be recovered from Eq. (8) as $h=\mathrm{exp}({h}^{*})$.

Since achieving convergence of the parameter estimates turned out to be difficult, we approximate initial values in linear space, i.e., for each value of *φ* = 0.01, 0.02, …, 10, we calculate $\Phi =\frac{\mathrm{log}n}{{n}^{\varphi }}$ and fit the following linear regression by *OLS*:

9 $$\mathrm{log}\left({r}_{n}\right)={\beta }_{h}+{\beta }_{A}\Phi +\acute{\text{o}}_n,$$

where $\acute{\text{o}}_n$ is an i.i.d. error term. To provide initial values to fit Eq. (8), we pick the solution of Eq. (9) where the root mean squared error is smallest and where ${\beta }_{A}>0$, then ${h}^{*}$ is initialized as ${\beta }_{h}$,${A}{^\prime}$ is initialized as $\mathrm{exp}{(\beta }_{A})$ and $b{^\prime}$ is initialized as $\mathrm{exp}({\varphi }_{m})$ where *φ*_*m*_ denotes the value of *φ* corresponding to the selected *Φ*.

As written above, the model is fit by log-least squares, i.e.${\left(\mathrm{log}\left(\widehat{{r}_{n}}\right)-\mathrm{log}\left({r}_{n}\right)\right)}^{2}=\mathrm{ log}{\left(\widehat{{r}_{n}}/{r}_{n}\right)}^{2}$ where ${r}_{n}$ and $\widehat{{r}_{l}}$ denote the observed and the predicted compression rate, respectively. To assess the model fit, we fit both Eqs. (8) and (9) to only the first 90% of the data points and use the last 10% as test data. Let *τ* = 1, 2, …, *Τ* denote the holdout data points. On this basis, we calculate the model fit as a measure of prediction accuracy^115^:

10 $${\rm M}=\frac{1}{\rm T}\sqrt{\sum_{\tau =1}^{\rm T}{\mathrm{log}\left(\widehat{{r}_{\tau }}/{r}_{\tau }\right)}^{2}}.$$

*Μ*-values are reported as percentages by multiplying the above equation by 100. Note that as long as the difference between ${r}_{\tau }$ and $\widehat{{r}_{\tau }}$ is relatively small, $\mathrm{log}(\widehat{{r}_{\tau }}/{r}_{\tau })\approx (\widehat{{r}_{\tau }}-{r}_{\tau })/{r}_{\tau }$. Thus, we can interpret *Μ* as measuring the approximate (absolute) average percentage difference between ${r}_{\tau }$ and $\widehat{{r}_{\tau }}$.

In order to avoid relying too much on the ansatz whose appropriateness can only be verified numerically, we additionally use the compression rate (denoted as *r* in what follows) at $\left\lfloor \frac{N}{m} \right\rfloor$ as an observed unbiased upper-bound-estimate for the underlying entropy rate.

### Synthetic dataset

The source emits two different symbols, “a” or “b”; for the first 2 *M* symbol tokens, symbols are emitted randomly. Thus, *h* = − (0.5 $\cdot$ log_2_(0.5) + 0.5 $\cdot$ log_2_(0.5)) = 1. For the second 2 M tokens, we generated a Hidden Markov Model sequence with memory 10 as described in Ref.^68^: if the last symbol is equal to the symbol observed 10 tokens before, the next symbol in the sequence will be “a” with probability *p* = 0.1 and “b” with *p* = 0.9. If the last symbol is not equal to the symbol observed 10 tokens before, the next symbol type will be “a” with probability *p* = 0.9 and “b” with *p* = 0.1. Thus, *h* = − (0.1 $\cdot$ log_2_(0.1) + 0.9 $\cdot$ log_2_(0.9)) ≈ 0.469. For the third 2 M tokens, we generated a pseudorandom sequence^68^: with probability *p* = 1, the next symbol in the sequence will be “b” if (i) the last symbol is both equal to the symbol observed 10 tokens before and also equal to the symbol observed 20 tokens before or (ii) the last symbol is both not equal to the symbol observed 10 tokens before and also not equal to the symbol observed 20 tokens before. In all other cases, the next symbol will be “a” with probability *p* = 1. Since prediction based on context is deterministic for this type of source, *h* = 0. We used PPM to compress successively larger chunks of the resulting corpus, proceeding in steps of 1000 symbols and calculated the local compression rate as the number of bits that PPM needs to compress the last 1000 symbols divided by 1000, i.e. ${r}_{l}^{loc}=R\left({X}_{l-{10}^{3}}^{l}\right)/{10}^{3}$.

### Evaluating the similarity of complexity rankings

For the LMM analyses, we regressed ${V}^{{c}_{1}}$ on a fixed effect for ${V}^{{c}_{2}}$ where $V$ denotes one of the following variables *r*, *h*, *H* and *H*_*Crúbadán*_. Both outcome and predictor were logged and computations were restricted to corpus pairs with at least 5 available shared languages. We fitted the following crossed-effects models^86,116^:

11 $${V}_{imag}^{{c}_{1}}={\beta }_{0}+{\beta }_{1}{V}_{imag}^{{c}_{2}}+{\mu }_{m}+{\alpha }_{a}+{\varsigma }_{g}+{\varepsilon }_{imag},$$

for *i* = 1,…, *I* different languages (identified by their ISO codes),* m* = 1,…, *M* macro-areas (Africa, Australia, Eurasia, North America, Papunesia or South America), *a* = 1,…, *A* countries and *g* = 1,…, *G* language families with ${\mu }_{m}\sim Gaussian(0,{\sigma }_{m}^{2})$; ${\alpha }_{a}\sim Gaussian(0,{\sigma }_{a}^{2})$; ${\varsigma }_{g}\sim Gaussian(0,{\sigma }_{g}^{2})$; ${\varepsilon }_{imag}\sim Gaussian(0,{\sigma }_{\varepsilon }^{2})$ all independently and where ${\sigma }_{m}^{2}$, ${\sigma }_{a}^{2}$, ${\sigma }_{g}^{2}$ and ${\sigma }_{\varepsilon }^{2}$ are the variances of ${\mu }_{m}$, ${\alpha }_{a}$, ${\varsigma }_{g}$ and ${\varepsilon }_{imag}$.The fixed portion of the model, ${\beta }_{0}+{\beta }_{1}{V}_{imag}^{{c}_{2}}$ is analogous to the linear predictor from a standard OLS regression and the random portion of the model, i.e. ${\mu }_{m}+{\alpha }_{a}+{\varsigma }_{g}+{\varepsilon }_{imag}$, incorporates group-specific shifts for language family, country and macro-area to account for genealogical and geographic relatedness of languages^86^, i.e. ${\sigma }_{m}^{2}$, ${\sigma }_{a}^{2}$ and ${\sigma }_{g}^{2}$ (languages were excluded from the analyses if information for one or more of the grouping factors was missing). All LMMs were fitted by restricted maximum likelihood (REML)^89^. Note that for some corpus pairs not all groups did vary, for example because all languages are located in one macro-area (e.g., in case of the European Constitution data, all languages are located in the Eurasian macro-area). In a similar vein, fitting an LMM does not make much sense if each group of each random factor consists of exactly one member. To solve this problem, our model automatically checks the composition of each grouping factor for each corpus pair and only included it if it consisted of at least two different groups and if at least one of those groups consisted of more than one member. Models were fitted with gradient-based maximization first. If gradient-based maximization did not converge, models were re-fitted with expectation–maximization (EM) only and we accepted any solution after a maximal number of EM iterations of 1000.

As shown in Ref.^89^, Eq. (27), the variance of the fixed component of the model, can be estimated as:

12 $${\sigma }_{f}^{2}=var\left({\beta }_{1}{V}_{imag}^{{c}_{2}}\right).$$

This can be computed by predicting values based on the estimated fixed effects of the model followed by a calculation of the variance of these fitted values. The variance of the full model can then be decomposed as:

13 $${\sigma }_{f}^{2}+{\sigma }_{m}^{2}+{\sigma }_{a}^{2}+{\sigma }_{g}^{2}+{\sigma }_{\varepsilon }^{2}.$$

On this basis, Ref.^89^, Eq. (26), define an *R*^2^ as measure of explained variance of the fixed portion (*m* indicates marginal *R*^2^) of the LMM as follows:

14 $${R}_{LMM(m)}^{2}=\frac{{\sigma }_{f}^{2}}{{\sigma }_{f}^{2}+{\sigma }_{m}^{2}+{\sigma }_{a}^{2}+{\sigma }_{g}^{2}+{\sigma }_{\varepsilon }^{2}}.$$

We calculated ${R}_{LMM(m)}^{2}$ for each model. To generate random baselines, we randomly permuted the values of ${V}^{{c}_{1}}$ and re-calculated ${R}_{LMM(m)}^{2}$ and proceeded as for the Spearman version described above. Our correlation measure is computed as follows:

15 $${\rho }^{LMM}=sign\left({\beta }_{1}\right)\sqrt{{R}_{LMM\left(m\right)}^{2}},$$

where sign(${\beta }_{1}$) returns the sign of ${\beta }_{1}$.

### Differences across populations

Above, we present results for *h* as outcome; in Supplementary Table 7 we present additional results for *r*, *H* and *H*_*Crubadan*_ as outcomes. To enhance convergence, the outcome was standardized per corpus, i.e. the corpus-specific mean was subtracted from each observed value and the result was divided by the corpus-specific standard deviation for the models with *h*, *r* and *H* as the outcome. As written in the main part of the paper, our covariate candidate set of models contains random intercepts for corpus, language family, language, macro-area, country and writing script and random slopes for corpus, language family, macro-area, country and writing script. All effects are assumed to be crossed. Note, however, that—in the terminology of Ref.^117^—countries are explicitly nested within macro-areas, i.e. each country occurs in exactly one macro-area. In the same sense, languages are explicitly nested within language families.

To compute differences in AIC, ΔAIC, we additionally fit LMMs without a fixed effect for speaker population size. Note that in models without a fixed effect for speaker population size, we also exclude potential random slopes. We then compute ΔAIC between the full model that includes a fixed effect and potential random slopes for speaker population size with a reduced model that does not include a fixed effect or random slopes for speaker population size but otherwise has the same fixed and random effect structure. We model all intercepts and slopes as i.i.d. and to be independently from each other. Models were fitted with gradient-based maximization and—since our primary focus in this set of analyses is on estimating and comparing different fixed effects structures—via maximum likelihood (ML)^118–120^. We accepted any solution after a maximal number of 100 iterations. Models with *H*_*Crubadan*_ as outcome do not contain a random effect for corpus (correspondingly *H*_*Crubadan*_ was not standardized), but models additionally contain fixed effects for text length, available number of documents (both logged) and a binary variable indicating whether the word frequency list is truncated (no/yes; see Supplementary Information: 3.7 for details). Of all converged model, we then selected the model with the lowest AIC and extract the corresponding estimate for *β*_1_ and its parametric (two-sided) *p-*value that is based on the absolute value of the *z*-statistic, defined as $z={\widehat{\beta }}_{1}/{\widehat{\sigma }}_{{\widehat{\beta }}_{1}}$ where ${\widehat{\sigma }}_{{\widehat{\beta }}_{1}}$ is the standard error of ${\widehat{\beta }}_{1}$.

### Analyses using CMIX

We downloaded the most current version (v19) of CMIX from https://byronknoll.com/cmix.html. For this analysis, each Bible translation^70^ was split into 66 separate books of the Biblical canon. We only kept translations with available information for all 39 books of the Old Testament (OT) of the Christian biblical canon. For languages with more than one available OT translation, we randomly sampled one translation. In total, we have available translations for 147 different languages. We used CMIX without further pre-processing and without an additional dictionary in each case. To compute $r({\mathrm{BibleOT}}_{CMIX}^{BPE})$, byte pair encoding^97^ was applied to each translation before compression. Following Ref.^54^, the number of BPE merges was set to 0.4· *V* where *V* is the number of different word types observed in a given translation. After tokenization into sub-word units, we replaced each distinct sub-word unit by a unique symbol and CMIX is then used to compress both the resulting symbol sequence and the mapping of sub-word units to 1–4 byte symbols in order to the calculate compression ratio for each available language *i* as follows:

16 $${r}_{i}\left({\mathrm{BibleOT}}_{CMIX}^{BPE}\right)=\frac{\left({R}_{i}^{seq}\left({\mathrm{BibleOT}}_{CMIX}^{BPE}\right)+{R}_{i}^{dic}\left({\mathrm{BibleOT}}_{CMIX}^{BPE}\right)\right)}{{N}_{i}^{chars}},$$

where ${R}_{i}^{seq}\left({\mathrm{BibleOT}}_{CMIX}^{BPE}\right)$ refers to the compressed length of the BP*-*encoded symbol sequence, ${R}_{i}^{dic}\left({\mathrm{BibleOT}}_{CMIX}^{BPE}\right)$ refers to the compressed length of the mapping of sub-word units to byte symbols and ${N}_{i}^{chars}$ denotes the length of text *i* in characters.

The Vox Clamantis data was pre-processed as follows. We use high quality phoneme level alignments for 29 languages that are based on the multilingual grapheme-to-phoneme (G2P) system Epitran available from https://osf.io/bc2ns/?view_only=ff23dd6bf3324b11b834ea4bd8d7e6c9^121^. Since the Vox Clamantis files are not aligned on the verse level, we processed the time-marked conversation (CTM) files to create a sequence of phonemes for each combination of bible chapter and ISO code. To map the Wilderness language codes to ISO codes, we used the information also provided by the Vox Clamantis team. If there were multiple phoneme sequences for an ISO code, we selected the longest available sequence. Our pre-processing R script for the Vox Clamantis data is also available from the repository accompanying the present article.

For each language, we then extracted a consecutive sequence of ${N}_{phoneme}$ =156832 phoneme tokens where ${N}_{phoneme}$ is equal to the length of the shortest available sequence (Tajiki). We then prepared representations of each sequence where each phoneme type is mapped to one 2 byte Unicode symbol. The compression ratio for each available language *i* is computed as follows:

17 $${r}_{i}\left({\mathrm{VoxClamantis}}_{CMIX}^{phoneme}\right)=\frac{\left({R}_{i}^{seq}\left({\mathrm{VoxClamantis}}_{CMIX}^{phoneme}\right)+{R}_{i}^{dic}\left({\mathrm{VoxClamantis}}_{CMIX}^{phoneme}\right)\right)}{{N}_{phoneme}},$$

where $({R}_{i}^{seq}\left({\mathrm{VoxClamantis}}_{CMIX}^{phoneme}\right)$ refers to the compressed length of the BP-encoded symbol sequence, ${R}_{i}^{dic}\left({\mathrm{VoxClamantis}}_{CMIX}^{phoneme}\right)$ refers to the compressed length of the mapping of sub-word units to byte symbols.

For the LMM models, we use the log of ${r}_{i}\left({\mathrm{VoxClamantis}}_{CMIX}^{phoneme}\right)$ as the outcome. Our covariate candidate set of models contains random intercepts and slopes for language family, macro-area and country. We accepted any solution after a maximal number of 1000 iterations. The remaining details are analogous to the other LMMs described above (see Methods: “Differences across populations”).

### Syllable patterns

In a computer simulation, we assumed that, in both languages L1 and L2, word tokens in a text have 1, 2, 3 syllables with probabilities 0.3, 0.5, 0.2. In L1, each syllable token in a randomly generated pseudo-text of 300 million phonemes length was taken to consist of one of five equiprobable consonants, followed by one of five equiprobable vowels. In L2, syllable tokens were set to have an onset of 1, 2, 3 consonants with probabilities 0.4, 0.3, 0.3 and a one-consonant coda with probability 0.5; for each of the three onset patterns, the permitted consonants resp. consonant clusters were assumed equiprobable, while syllables with a coda were set to have final /s/ and /r/ with probabilities 0.7 and 0.3. When the five vowels are taken to be equiprobable in both L1 and L2 for all syllable tokens, then the PPM-based compression rate of random L2 texts converged toward a minimally smaller number than for L1, with ${r}_{{L}_{1}}\approx 0.289$ and ${r}_{{L}_{2}}\approx 0.288$. After we changed the L2 vowel probabilities for non-word-initial syllables to 0.01, 0.9, 0.04, 0.01, 0.04 for /a/, /e/, /i/, /o/, /u/, keeping all other parameters, then, for L2, the average compression rate dropped to ${r}_{{L}_{2}}\approx 0.262$.

### Supplementary Information

Supplementary Information.

## Data Availability

All parallel text data were taken from the sources mentioned in the supplementary information. Code and data are described in Supplementary Information: “Code and Data” and are available at https://osf.io/f5mke/.
