# Supplementary material for: A large quantitative analysis of written language challenges the idea that all languages are equally complex
Source: Sci Rep. 2023 Sep 16;13:15351. doi: 10.1038/s41598-023-42327-3 (PMC10505229; doi:10.1038/s41598-023-42327-3)
Supplement: Supplementary file 1 — Supplementary Information. [file 41598_2023_42327_MOESM1_ESM.pdf]

Supplementary Information for

A large quantitative analysis of written language challenges the idea that all languages are equally complex

Alexander Koplenig, Sascha Wolfer, Peter Meyer
Correspondence to: koplenig@ids-mannheim.de

Contents

S1. *PPM* as a language learner ... 4

S2. *Ansatz* functions ... 6

S3. Corpora ... 10

S3.1 Dataset description ... 12

S3.2 Data preparation and compression ... 13

S3.3 Religious texts ... 17

S3.3.1 BibleNT (parallel) ... 17

S3.3.2 BibleOT (parallel) ... 19

S3.3.3 WatchtowerV1 (parallel) ... 20

S3.3.4 WatchtowerV2 (parallel) ... 21

S3.3.5 Quran (parallel) ... 22

S3.4 News/Web/Wikipedia crawls ... 23

S3.4.1 GlobalVoices (comparable) ... 23

S3.4.2 LCCnews/ LCCweb/ LCCwiki (comparable) ... 24

S3.5 Legalese texts ... 25

S3.5.1 UDHR (parallel) ... 25

S3.5.2 EUconst (parallel) ... 26

S3.5.3 Europarl (parallel) ... 27

S3.5.4 EUmed (parallel) ... 28

S3.5.5 UNPC (parallel) ... 30

S3.6 Subtitles ... 32

S3.6.1 MSub01 – MSub13 (parallel) ... 32

S3.6.2 TEDt01 – TEDt09 (parallel) ... 34

S3.7 Other ... 36

S3.7.1 Ubuntu (comparable) ... 36

S3.7.2 GoogleT (parallel) ... 38

S3.7.3 TatoebaV1 (comparable) ... 40

S3.7.4 TatoebaV2 (comparable) ... 41

S3.8 Wordlist: Crúbadán 42
S4. Code and data 44
S5. Coverage 47
S6. Correlation matrix 48
S7. Relative standard deviations for *h* and *r* 49
S8. Correlations between entropy rates and learning difficulty 50
S9. Evaluating the similarity of complexity variables 53
S10. Overview of the Linear Mixed-effects models 55
S11. Testing different compression algorithms 56
References 58

S1. *PPM* as a language learner

In Supplementary Table 1, we illustrate that every time *PPM* encounters new text, it updates its language-model (i.e. its probability distribution) and with growing input, it gets better in compressing/predicting subsequent linguistic data (Takahira, Tanaka-Ishii & Dębowski 2016).

Supplementary Table 1 | Illustration of *PPM* in action. 1st column: on the level of words, we trained a *PPM* model of order 5 to predict the next 5 words; on the level of characters, we trained a *PPM* model of order 10 to predict the next 30 characters [prediction also ends when the end of a sentence is reached; both models were smoothed by linearly interpolating all probabilities, i.e. predictions of *n*-grams of all orders are blended together (Knoll & Freitas 2012; Koplenig, Kupietz & Wolfer 2022). Higher weights are assigned to *n*-grams of higher order, where individual weights are determined by calculating the number of different words observed after a specific *n*-gram]. 2nd column: models were trained on the entire Project Gutenberg works of Mark Twain (2004). Size refers to the number of sentences used for training. 3rd column: the context is presented in bold face, predictions are in regular font. Characters were mapped to lower-case and punctuation marks were treated as words. Output was manually corrected for capitalization to improve readability.

Symbol	Size	**Context** Prediction
Word	10^1	**I was ashamed of** all the swarms that come ...
	10^2	**I was ashamed of** himself he did bet half ...
	10^3	**I was ashamed of** the country to part with ...
	10^4	**I was ashamed of** myself and felt unspeakably ridiculous ...
	10^5	**I was ashamed of** my performance at the time ...
	10^1	**It reminds** to the will of god.
	10^2	**It reminds**, and then wilted to the ...
	10^3	**It reminds**, and the rest of the ...
	10^4	**It reminds** one that it was not ...
	10^5	**It reminds** me of a tale that ...
Character	10^1	**He died a**nd made him feel good.
	10^2	**He died a**nd registered their names on t…
	10^3	**He died a**nd took the crown of thorns.
	10^4	**He died a**nd was buried in a couple of f…
	10^5	**He died a**t the same time the ship was t…
	10^1	**So we spe**riences of my life, this secre ...
	10^2	**So we spe**ed of two or three hours the s ...
	10^3	**So we spe**nt part of a book of mine on t ...
	10^4	**So we spe**nt a week there, at the very s ...
	10^5	**So we spe**nt a moment or two and a half ...

Or put differently, *PPM learns* to exploit the statistical structure of the input, paralleling human language learning (Newport 2016; Chater & Vitányi 2007) with interesting applications in natural language processing (Teahan et al. 2000; Teahan & Harper 2003), language production (Ward &

MacKay 2002; Knoll & Freitas 2012) and – more generally – machine learning of patterns to predict (into) the future (Mahoney 2013; Mahoney 2005; Knoll & Freitas 2012).

Further details and – given that the examples in Supplementary Table 1 are cherry-picked – training data, trained (unsmoothed and smoothed) *n*-gram models and an open source Java program to interactively test *PPM* are available at https://osf.io/f5mke/. In addition, the provided Jupyter notebook *smoothLM.ipynb* can be used to generate examples for any text file.[1]

[1] To demonstrate that *PPM* learns to predict with growing input, the notebook produces examples (both on the level of characters and on the level of words) based on 5%, 10%, 20%, 40%, 80% and 100% of the input text.

S2. *Ansatz* functions

Since, as written in the manuscript, the (probabilistic) relationship between (the convergence of) h and L is unknown (Takahira, Tanaka-Ishii & Dębowski 2016), we tested several *ansatzes* that have been discussed in the literature (Schürmann & Grassberger 1996; Ebeling & Nicolis 1991; Takahira, Tanaka-Ishii & Dębowski 2016; Moscoso del Prado Martín, 2011) as summarized in Supplementary Table 2.

Each *ansatz* was fitted both with least-squares, i.e. by minimizing the sum of $\acute{o}_n^2 = (r_n - \widehat{r_n})^2$, and with log least-squares, i.e. by minimizing the sum of $\acute{o}_n^2 = (\log r_n - \log \widehat{r_n})^2 = [\log(r_n/\widehat{r_n})]^2$.

To evaluate the performance of the different *ansatzes* with regard to our project, we used several criteria:

(i) The model fit, i.e. the prediction accuracy error between the held-out observed compression rates and the predicted ones (cf. Methods: equations (8)).

(ii) The number of iterations that are needed for convergence, with the maximum number of possible iterations set to 100.

(iii) The convergence rate, i.e. how many times (in %) convergence was achieved.

(iv) The Spearman correlation between the extrapolated entropy rate *h* and the unigram entropy H (cf. Methods: equations (1)).

(v) The Spearman correlation between the extrapolated entropy rate h and the compression rate *r* at $\lfloor N/m \rfloor$.

(vi) The lower quartile (25th percentile) for h.

Those criteria were tested for the following four corpora (cf. section S3):

- BibleOT (all five rounds, cf. section S3.3.2)
- Europarl
- UDHR
- Watchtower (Version 1)

Supplementary Table 3 summarizes the results. Criterion (i) shows that all three *ansatzes* have excellent model fits with an average accuracy ratio below 1% for all corpora but one. In general, using log least-squares turns out to be preferable compared to least-squares for all three *ansatzes*. In most cases, *a2* has the best accuracy ratio followed by *a3*. Since our aim is to fit a lot of different

texts with widely varying characteristics (especially in terms of text length) on both the level of characters and the level of words, convergence properties are highly relevant.

Supplementary Table 2 | Tested *ansatzes*. *a3* is our own synthesis of *a1* and *a2*.

Ansatz	Form	Fitted form	Source
a1	$r_n = h + A * \log n / n^b$,<br>$h > 0, A > 0, b > 0$	$r_n = \exp(h') + \exp(A') * \log n / n^{\exp(b')} + ó_n$	(Schürmann & Grassberger 1996; Eq. 28)
a2	$r_n = \exp(h^* + A * 1/n^b)$,<br>$A > 0$, $b > 0$, h=$\exp(h^*)$	$r_n = \exp\left(h^* + \exp(A') * 1/n^{\exp(b')}\right) + ó_n$	(Takahira, Tanaka-Ishii & Dębowski 2016; Eq. 8)
a3	$r_n = \exp(h^* + A * \log n / n^b)$,<br>$A > 0$, $b > 0$, h=$\exp(h^*)$	$r_n = \exp\left(h^* + \exp(A') * \log n / n^{\exp(b')}\right) + ó_n$	-

In this context, criteria (ii) and (iii) show that *a2* needs both more iterations to converge and does not converge at all in a substantial amount of the cases. Both *a1* and *a3* clearly outperform *a2* here, needing fewer iterations to converge and converging in all cases. The idea of (iv) is based on a result of Bentz et al. (2017) who report a strong linear relationship between unigram entropies and entropy rates on the level of words and put this into perspective to the results of Montemurro and Zanette (2011). Using both *a1* and *a3*, this positive relationship is replicated for all test datasets and on both symbolic levels. For *a2*, there is no emerging trend; on the contrary: while there are some datasets for which the statistical relationship between *h* and *H* is clearly positive, there are several cases for which the relationship is strongly negative. This is rather counterintuitive. For example, it would indicate that the harder it is to predict a word without knowledge of the context (i.e. higher *H*), the easier it is to predict when the preceding context is known (i.e. lower *h*). Criterion (v) further corroborates this result: while there is a (moderate to strong) positive relationship between *h* and *r* for both *a1* and *a3*, the results again are very mixed for *a2* with both strongly positive and strongly negative relationships. This gives rise to the impression that there might be a problem that occurs when estimating *h* based on *a2*. Therefore, we calculated the lower quartile for *h* for the four test datasets on both symbolic levels. Again, *a2* clearly stands out, e.g. for 25% of the texts of the Bible and the Watchtower corpora, the limiting entropy rate based on *a2* is effectively zero. This would indicate that once the optimal prediction scheme has been learned, it would be possible to predict subsequent words based on the preceding context with zero uncertainty. From a theoretical point of view, this result seems rather implausible. On the other hand, for both *a1* and *a3*, all calculated lower quartiles have $h \gg 0$. In addition, the results seem

to be much more plausible, e.g. between three and eleven guesses are needed on average to correctly predict the next word based on the context for 25% of the analysed texts.

In view of these considerations, we chose *ansatz a3* with a log-normal error structure for all analyses presented in our paper. But, as written above, to avoid relying too strongly on an *ansatz* whose validity can only be verified numerically, we also use *r* as an unbiased upper bound estimate for *h*.

Supplementary Table 3 | Results for the different *ansatzes*. Values are rounded for illustration purposes only throughout this document.

Criterion	Corpus	Symbol	N	*Ansatz a1*		*Ansatz a2*		*Ansatz a3*	
				Least squares	Log least squares	Least squares	Log least squares	Least squares	Log least squares
**(i) Accuracy ratio**	BibleOT	Character	1,270	0.075	0.059	0.048	0.040	0.071	0.058
	Europarl		21	0.019	0.013	0.006	0.005	0.013	0.010
	UDHR		452	0.998	0.593	0.435	0.320	0.503	0.378
	Watchtower V1		142	0.162	0.105	0.111	0.076	0.165	0.111
	BibleOT	Word	1,270	0.074	0.053	0.046	0.038	0.081	0.060
	Europarl		21	0.005	0.003	0.007	0.005	0.004	0.002
	UDHR		452	1.289	0.605	0.352	0.265	0.852	0.449
	Watchtower V1		142	0.197	0.110	0.130	0.084	0.222	0.134
**(ii) Number of iterations**	BibleOT	Character	1,270	3.81	4.28	7.79	6.72	4.23	3.77
	Europarl		21	4.24	4.86	3.33	3.19	3.95	3.90
	UDHR		452	3.79	5.06	4.63	3.47	3.87	3.27
	Watchtower V1		142	4.65	5.38	11.32	7.39	5.48	3.94
	BibleOT	Word	1,270	3.39	4.24	33.71	30.42	4.64	3.61
	Europarl		21	2.95	3.29	3.86	3.43	3.90	3.14
	UDHR		452	4.59	6.38	3.86	3.13	4.33	3.49
	Watchtower V1		142	4.14	5.76	44.29	34.82	6.85	4.15
**(iii) Convergence rate (%)**	BibleOT	Character	1,270	100.0	100.0	97.9	98.3	100.0	100.0
	Europarl		21	100.0	100.0	100.0	100.0	100.0	100.0
	UDHR		452	100.0	100.0	99.8	100.0	100.0	100.0
	Watchtower V1		142	100.0	100.0	97.9	98.6	100.0	100.0
	BibleOT	Word	1,270	100.0	100.0	75.4	78.0	100.0	100.0
	Europarl		21	100.0	100.0	100.0	100.0	100.0	100.0

	UDHR		452	100.0	100.0	100.0	100.0	100.0	100.0
	Watchtower V1		142	100.0	100.0	67.6	76.1	100.0	100.0
**(iv) Spearman correlation between *h* and *H***	BibleOT	Character	1,270	0.25	0.23	-0.14	-0.14	0.17	0.15
	Europarl		21	0.48	0.50	0.31	0.34	0.41	0.39
	UDHR		452	0.67	0.63	0.30	0.25	0.55	0.49
	Watchtower V1		142	0.28	0.23	-0.35	-0.39	0.21	0.16
	BibleOT	Word	1,270	0.48	0.35	-0.69	-0.71	0.62	0.54
	Europarl		21	0.67	0.67	-0.30	-0.42	0.62	0.60
	UDHR		452	0.86	0.83	0.85	0.78	0.88	0.84
	Watchtower V1		142	0.88	0.71	-0.52	-0.68	0.91	0.87
**(v) Spearman correlation between *h* and *r***	BibleOT	Character	1,270	0.58	0.54	-0.13	-0.15	0.52	0.48
	Europarl		21	0.92	0.92	0.76	0.76	0.88	0.86
	UDHR		452	0.94	0.92	0.63	0.58	0.85	0.81
	Watchtower V1		142	0.58	0.45	-0.36	-0.49	0.55	0.43
	BibleOT	Word	1,270	0.59	0.46	-0.61	-0.64	0.74	0.65
	Europarl		21	0.86	0.87	-0.04	-0.18	0.83	0.82
	UDHR		452	0.98	0.97	0.91	0.88	0.97	0.96
	Watchtower V1		142	0.94	0.79	-0.42	-0.61	0.96	0.93
**(vi) Lower quartile for *h***	BibleOT	Character	1,270	0.85	0.82	0.33	0.29	0.74	0.71
	Europarl		21	0.99	0.96	0.79	0.78	0.90	0.89
	UDHR		452	1.82	1.69	1.02	1.07	1.36	1.34
	Watchtower V1		142	0.93	0.83	0.29	0.18	0.79	0.71
	BibleOT	Word	1,270	4.26	3.98	0.22	0.09	3.85	3.63
	Europarl		21	5.22	5.14	2.70	2.84	4.73	4.67
	UDHR		452	11.52	10.41	5.84	6.18	9.83	9.00
	Watchtower V1		142	5.12	4.17	0.17	0.01	4.55	3.82

S3. Corpora

Supplementary Table 4 presents an overview of each corpus used in this study. Further details on each corpus are given in the subsequent sections.

Supplementary Table 4 | Overview of all corpora used in the study. 1st column: Name of the corpus. 2nd column: Text category (R – religious texts, NW – news/web/Wikipedia crawls, L – legalese texts, S – subtitles, O – other, W – word frequency list). 3rd column: Type of corpus (P – parallel, C – comparable, W – word frequency list). 4th column: source. 5th column: number of available translations. 6th column: number of different languages (*ISO*-639-3 codes). 7th/8th column: compression chunk size in words/characters. 9th/10th column: median text length in words/characters. 11th/12th column: median accuracy ratio for words/characters. 13th column: section cross reference for further details.

Corpus	Corpus category	Corpus type	Source	Translations	Languages	Chunk size		Length		Accuracy (%)		Cross reference
						Words	Characters	Words	Characters	Words	Characters	
**BibleNT**	R	P	(Mayer & Cysouw 2014)	1,459	1,093	100	1,000	227,391	1,190,294	0.08	0.08	S3.3.1
**BibleOT**	R	P		254	147	1,000	10,000	642,772	3,259,354	0.08	0.08	S3.3.2
**WatchtowerV1**	R	P		142	140	100	1,000	129,008	659,563	0.14	0.11	S3.3.3
**WatchtowerV2**	R	P		265	260	5	50	7,194	35,608	0.24	0.16	S3.3.4
**Quran**	R	P	http://tanzil.net/trans/; accessed 4/30/20	43	43	100	1,000	182,950	860,590	0.09	0.09	S3.3.5
**GlobalVoices**	NW	C	http://casmacat.eu/corpus/global-voices.html; accessed 4/30/20	40	39	10	100	20,021	112,541	0.17	0.17	S3.4.1
**LCCnews**	NW	C	(Goldhahn, Eckart & Quasthoff 2012)	112	85	1,000	10,000	196,899	1,119,551	0.05	0.03	S3.4.2
**LCCweb**	NW	C		87	85			195,953	1,127,076	0.06	0.03	
**LCCwiki**	NW	C		171	171			185,770	1,038,774	0.07	0.04	
**UDHR**	L	P	https://unicode.org/udhr/; accessed 4/30/20	452	399	10	100	1,978	10,822	0.44	0.34	S3.5.1
**EUconst**	L	P	(Tiedemann 2012)	21	21	100	1,000	92,607	620,502	0.15	0.10	0
**Europarl**	L	P		21	21	1,000	10,000	5,362,935	31,959,314	0.00	0.01	S3.5.3
**EUmed**	L	P		22	22	1,000	10,000	3,241,844	18,714,208	0.09	0.10	S3.5.4
**UNPC**	L	P		6	6	50,000 for the 1st 1*M* symbols, then every 500,000 symbols		341,723,872	879,903,168	0.01	0.00	S3.5.5
**MSub01**	S	P	(Levshina 2016)	29	29	10	100	7,349	34,207	0.25	0.26	S3.6.1
**MSub02**	S	P		26	26			9,695	43,988	0.24	0.27	
**MSub03**	S	P		37	37			4,661	20,444	0.39	0.29	
**MSub04**	S	P		16	16			12,690	60,718	0.21	0.23	
**MSub05**	S	P		15	15			8,908	42,103	0.25	0.24	
**MSub06**	S	P		27	27			7,832	34,262	0.30	0.28	

**MSub07**	S	P		12	12			17,317	79,075	0.16	0.20	
**MSub08**	S	P		9	9			9,598	46,148	0.26	0.25	
**MSub09**	S	P		15	15			10,303	49,983	0.22	0.25	
**MSub10**	S	P		28	28			10,937	53,059	0.22	0.21	
**MSub11**	S	P		14	14			9,679	45,133	0.26	0.29	
**MSub12**	S	P		30	30			5,516	25,804	0.35	0.27	
**MSub13**	S	P		14	14			7,533	34,984	0.33	0.34	
**TEDt01**	S	P		50	50			3,075	14,455	0.49	0.42	
**TEDt02**	S	P		60	59			3,555	17,360	0.33	0.25	
**TEDt03**	S	P		61	60			3,763	18,468	0.38	0.30	
**TEDt04**	S	P	https://amara.org/en/teams/ted/videos/; accessed 4/30/20	31	31			2,099	10,409	0.43	0.33	
**TEDt05**	S	P		31	31			1,853	8,658	0.54	0.37	S3.6.2
**TEDt06**	S	P		28	28			1,369	6,318	0.62	0.48	
**TEDt07**	S	P		74	71	5	50	1,376	7,017	0.61	0.45	
**TEDt08**	S	P		49	48			770	3,669	0.45	0.25	
**TEDt09**	S	P		52	51			562	2,746	0.67	0.65	
**Ubuntu**	O	C	(Tiedemann 2012)	86	86			5,780	35,888	0.33	0.25	S3.7.1
**GoogleT**	O	P	https://translate.google.com; accessed 4/30/20	102	102			716	3,875	0.43	0.44	S3.7.2
**TatoebaV1**	O	C	https://tatoeba.org/eng/downloads; accessed 4/30/20	183	183			656	3,034	0.59	0.30	S3.7.3
**TatoebaV2**	O	C		123	123			3,438	15,710	0.35	0.26	S3.7.4
**Crúbadán**	W	W	(Scannell 2007)	2,216	1,943			101,079				S3.8

S3.1 Dataset description

Supplementary Figure 1 visualizes several important aspects of our dataset, see Methods: Corpora for details.

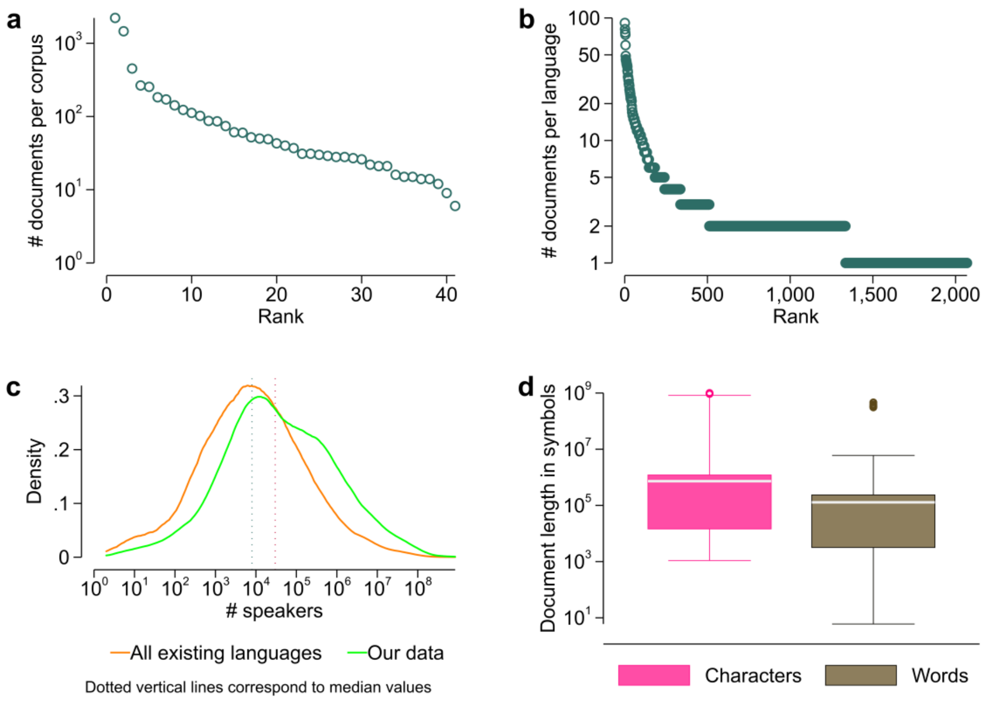

Supplementary Figure 1 | Dataset description (a) Number of documents per corpus. Corpora are ranked by the number of available documents. (b) Number of documents per language. Languages are ranked by the number of available documents. (c) Kernel density estimations of the number of speakers per language. Orange line – all existing languages. Lime line – languages for which we have available documents in our database. Dotted vertical lines correspond to median values. (d) Distribution of document length in characters/words.

S3.2 Data preparation and compression

Each text analyzed in this study is tokenized and Unicode normalized where necessary (see https://en.wikipedia.org/wiki/Unicode_equivalence#Normal_forms). All uppercase characters were lowered based on the closest language-specific *ISO*-639-3 code (henceforth: *ISO*). Unless otherwise specified, the word-break algorithm of the *International Components for Unicode* library (Unicode Consortium 2019; Annex #29) was used to detect word boundaries (in texts without spaces or marks between words, a dictionary lookup method is used by the algorithm (Koplenig 2018))[2]. To use the *PPM*d algorithm on the level of words as symbols, each unique word type is replaced by one unique Unicode symbol.[3] To determine the order of the *7-zip PPM*d

[2] Dash-separated words (e.g. “presidency-in-office”) are treated as one word. Note that Stata's Unicode word-splitting routine seems to not consistently split words with apostrophes. This could be a problem in languages such as French or Italian, e.g. "l'article" in French should evidently be split into two words "l'" and "article". However, since Stata did not seem to include the corresponding rule *WB5a* (Unicode Consortium 2019; Annex #29), "l'article" is not split into two words. To check if this problem could affect our results, we use the 16 Tree-tagged translations of the European Constitution (see section "Limitations/extensions" in the main part of the paper). Here splitting works correctly. We then calculated Spearman correlations between the original and the Tree-tagged version for h, r and L on both symbolic levels. All correlations are higher than 0.96, except for the correlation for h on the level of characters, here $\rho = 0.83$. This indicates that the inconsistency in Stata's Unicode word-splitting routine does not systematically bias our results.

[3] Note that 63,487 of the 1,112,063 (5.71%) available Unicode symbols are less than 4 bytes long. This is due to historical reasons, for example, all 127 characters with a length of 1 byte belong to the 128 characters of the American Standard Code for Information Interchange (*ASCII*) character encoding standard that is based on the “English” alphabet. Since we randomly assign unique Unicode symbols to word types, this variation-in-length should not systematically affect the results. To test this, we used the Quran corpus (see S3.3.5) and only used the 1,048,576 Unicode symbols with a length of 4 bytes for the replacement procedure and then re-ran the analyses on the level of words. As expected, the Spearman correlation between the original version and the 4-bytes-only version is 0.9998 for r and 0.9974 for h. Further note that we show in Figure 4a,b that our results also hold for lemmatized texts.

Perhaps more importantly, the fact that *ASCII* characters have shorter byte lengths could imply that our results could systematically be biased for languages that predominantly use *ASCII* characters, especially English. To test this, we replicated the compression for the $N = 43$ languages of the Quran corpus on the level of characters and replaced each unique original character by a unique 4-byte Unicode symbol. We find that the Spearman correlation between the original version and the 4-bytes-only version is ~0.95 for r and ~0.93 for h. If we only include the $N = 21$ languages that use *ASCII* characters (defined as corresponding documents where at least one of the first 1,000 characters is any character from a, b, …, z), the Spearman correlation for r is ~0.99. The correlation coefficient is still high but a little

model, the full string is first compressed with several available options in the range of [2, 32].[4] As suggested by Takahira et al. (2016), the value that resulted in the smallest encoding rate is used for all further compressions, i.e. on both levels (words and characters) the resulting strings are compressed after every *m* symbols as described above and the compression rate in bits (minus the header) is stored, resulting in the series of compression rates to which the *ansatz* function can be fit.

In general, languages are identified by their *ISO-639-3* codes. Some corpora use *ISO*-639-2 codes. In those cases, we used a Wikipedia list to map the *ISO*-639-2 codes to *ISO*-639-3 codes available at https://en.wikipedia.org/wiki/List_of_ISO_639-2_codes.

In total, we analysed 41 different multilingual corpora by compressing ~30.2*M* (sub-)strings of varying lengths. Supplementary Figure 2 visualizes how we adapt Shannon's information-theoretic view of communication to analyse parallel corpora: [1] a source message is [2] encoded into a signal, i.e. translated into different languages (Weaver 1949). [3] To estimate the average per-symbol information content, we use *Prediction by partial matching* (*PPM*), a prominent computational language model originally developed for data compression (Cleary & Witten 1984; Chen & Goodman 1996) to calculate the compression rate *r* (compressed size/message length *N* in symbols) as an index of (un)predictability/complexity for both words and characters as information encoding units (Weaver 1949). [4] Since *r* only provides upper bounds for *h*, we calculate *r* for subsets of increasing length (red circles) and fit a nonlinear *ansatz* (Takahira, Tanaka-Ishii & Dębowski 2016; Schürmann & Grassberger 1996) (yellow line) with interval constraints by log-least squares where initial values are approximated in linear space to estimate the asymptotic value of *h* (blue line; see Methods: Estimating entropy and S2: Ansatz functions for details).

---

lower for *h* with $\rho_s = 0.86$. However, the difference between the correlation coefficient for this restricted dataset and the correlation coefficient for all available documents is not significant ($z = -1.236$; $p = 0.2163$).

[4] With $o = 2$ being the minimum and $o = 32$ the respective maximum order. We tested the following possible values: 2, 3, 4, 5, 6, 7, 8, 9, 10, 12, 14, 16, 18, 20, 24, 28, 32. The following command line syntax was used: 7z a -t7z -m0=PPMd -mx9 -mo=*o* -ms=off -mmem=2000m -mmt=off "*directory**output_archive*.7z" "*directory**input_text*.txt".

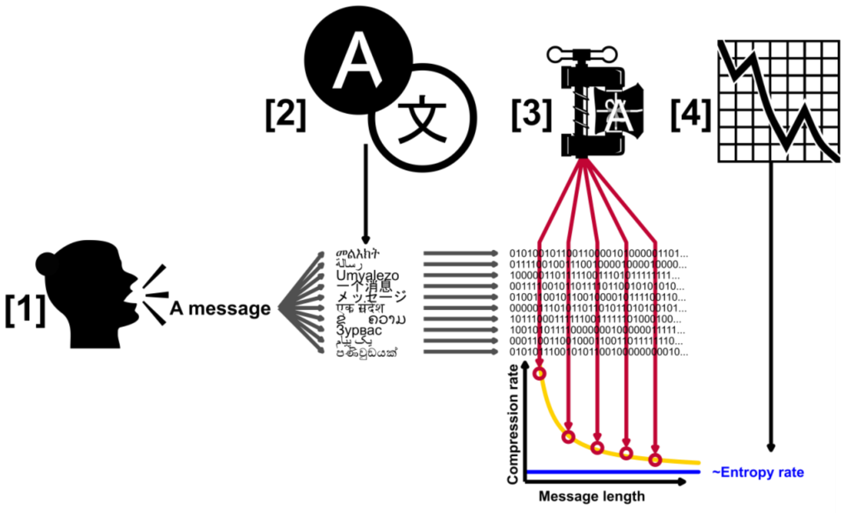

Supplementary Figure 2 | Adaptation of Shannon's information-theoretic view of communication for the analysis of parallel corpora.

As described in the main part of the paper, our *ansatz* has three parameters: the limiting entropy rate *h*, a proportionality constant *A* and an exponent *b*. While *h* quantifies how difficult it is to *predict*, *b* quantifies how difficult it is to *learn to predict* (Takahira, Tanaka-Ishii & Dębowski 2016). Supplementary Figure 3 illustrates that lower *b*-values are indicative of slower convergence, i.e. learning is more difficult.

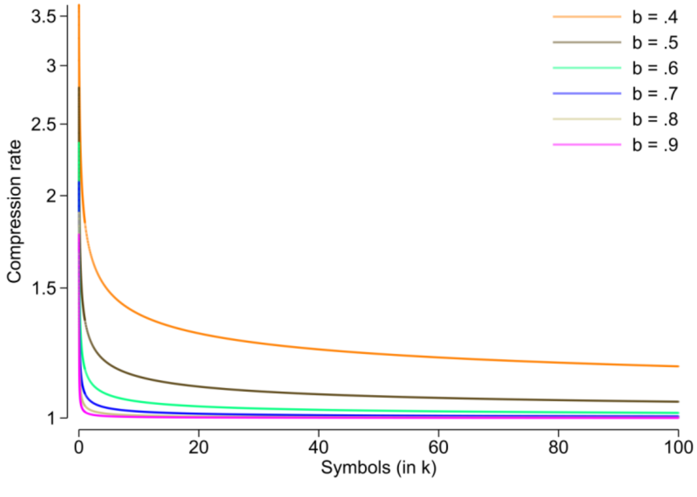

Supplementary Figure 3 | Compression rate *r* as a function of the number of symbols (*l*) for different values of *b*. Compression rates are calculated as $r = \exp\left(h' + 1.40 * \log n / n^b\right)$ where $h' = log(1.00)$.

As can be seen, higher values of *b* cause a faster decay indicating that it is *less* difficult to learn to *predict* (Takahira, Tanaka-Ishii & Dębowski 2016).

S3.3 Religious texts

S3.3.1 BibleNT (parallel)

The data are part of the *Parallel Bible corpus* (henceforth: *PBC*) made available by Mayer and Cysouw (2014) containing a total of 1,568 unique translations of the Bible in a very fine-grained parallel structure (regarding book, chapter and verse).[5] Each translation is already tokenized and Unicode normalized and spaces are inserted between words as well as punctuation marks and non-alphabetic symbols by Mayer and Cysouw (2014). In addition, all texts were manually checked and corrected by Mayer and Cysouw (2014) where necessary (also see Bentz et al. 2017: Appendix A). In some texts without spaces or marks between words (e.g. for Khmer, Burmese, or Mandarin Chinese), we used the dictionary lookup method mentioned above to detect word boundaries with detected word tokens then being space-separated. Afterwards, we split each Bible translation into different books of the biblical canon and aggregated all books of the New Testament (only translations with available data for each New Testament (*NT*) book were included). Beside the actual text, each element of each line of each Bible translation document contains information about the book, chapter and verse number (Mayer & Cysouw 2014); we used that information to create five random verse orders that are used to randomize the verse order for every translation in a fully parallel way and thus effectively discard all supra-verse information.

For 1,459 translations, data for all 27 books of the *NT* was available, so we compressed a total of 1,459 x 5 different verse shuffles x 2 different symbolic levels = 14,590 texts. With a chunk size of m = 100 on the level of words and m = 1,000 on the level of characters, we compressed a total of 2,6315,705 different substrings.[6]

Plot a of Supplementary Figure 4 visualizes the distribution of accuracy ratios on both symbolic levels (words/characters). In addition, plot b depicts the document with the worst fit (i.e. the

---

[5] The question whether different translations of the Bible can be considered *translational equivalents* is discussed in Koplenig (2021).

[6] The *ansatz* function is separately fitted to each of the five different versions and estimated parameters are averaged per translation. Note that in the final dataset, both for the number of iterations needed for convergence and for the accuracy ratio the maximum across the five different versions is reported.

document with the highest accuracy ratio) and the document whose accuracy ratio is closest to the upper quartile (75th percentile), i.e. 75% of the documents have a better accuracy ratio (i.e. a lower accuracy ratio). Separate plots are given for both symbol levels (words/characters). As can be seen, the fit is very good.

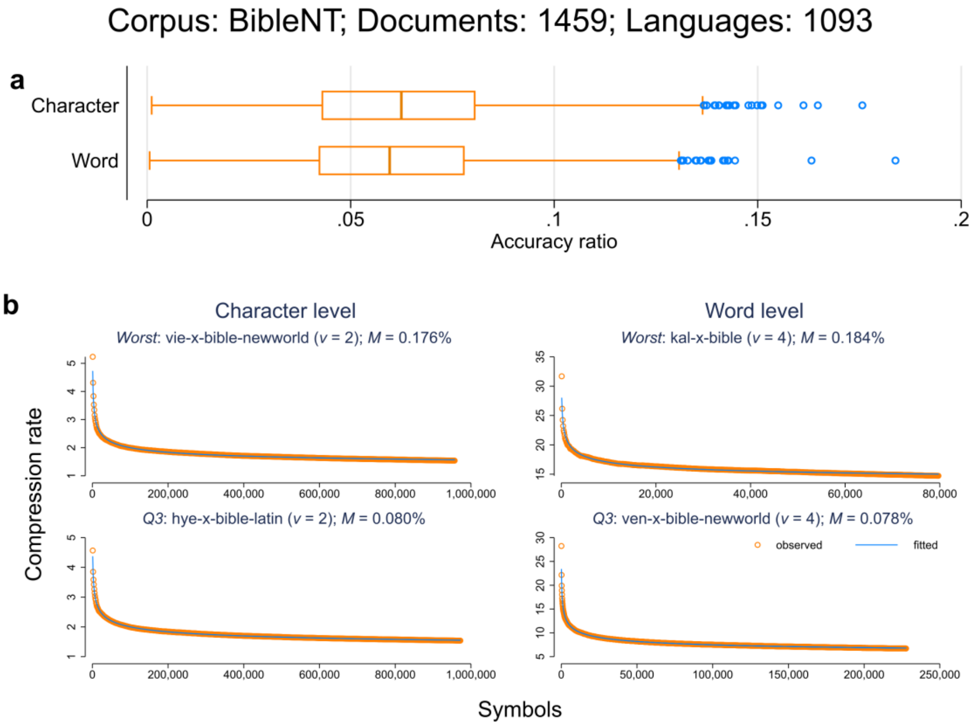

Supplementary Figure 4 | Corpus – BibleNT. Plot a – Distribution of accuracy ratios on both symbolic levels (words/characters). Plot b – First row: visualization for the document with the worst fit (i.e. maximal accuracy ratio). Second row: visualization for the document whose accuracy ratio is closest to the upper quartile (Q3, i.e. 75% of the documents have a better accuracy ratio). Left column: character level. Right column: word level.

S3.3.2 BibleOT (parallel)

Source and setup in analogy to the BibleNT data (cf. section S3.3.1). Here, only translations where all 39 books of the Old Testament (*OT*) of the Christian biblical canon are available are used. In total, we compressed 254 x 5 different verse shuffles x 2 different levels = 2,540 texts. With a chunk size of 1,000 on the level of words and 10,000 on the level of characters, we compressed a total of 1,222,165 different substrings. Supplementary Figure 5 visualizes the model fits.

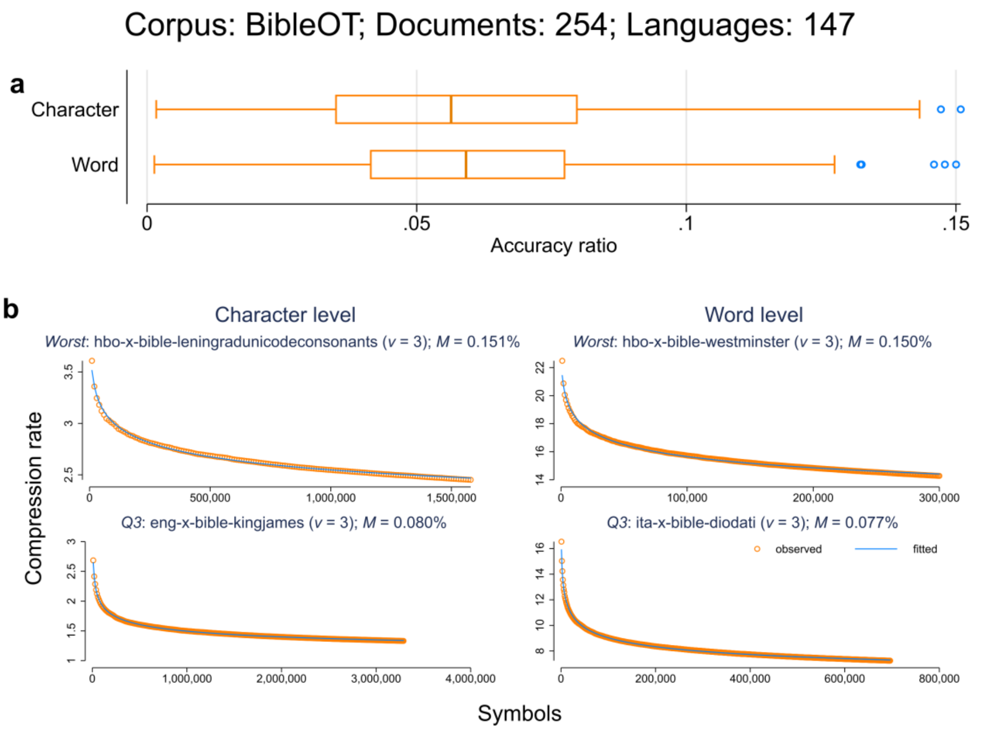

Supplementary Figure 5 | Corpus – BibleOT. Cf. Supplementary Figure 4 for a description of the content.

S3.3.3 WatchtowerV1 (parallel)

The data are also part of the *PBC* (2014). This part contains a total of 311 unique translations of some introductory texts of the Jehovah's Witnesses' official web site (Cysouw & Wälchli 2007). The texts are provided in a similar format as the BibleNT data (cf. section S3.3.1). We extracted 3,230 verses that are available in 142 different translations: 960 from the *Bible Teach* ("bh") section (872 from the articles section and 88 from the appendix section) and 2,270 from the *My Book of Bible Stories* ("my") section. A list of the extracted verses is available as part of the published code (cf. section S4). The verse order for every translation is randomly shuffled in a fully parallel way. With a chunk size of 100 on the level of words and 1,000 on the level of characters, we compressed a total of 279,549 different substrings. Supplementary Figure 6 visualizes the model fits.

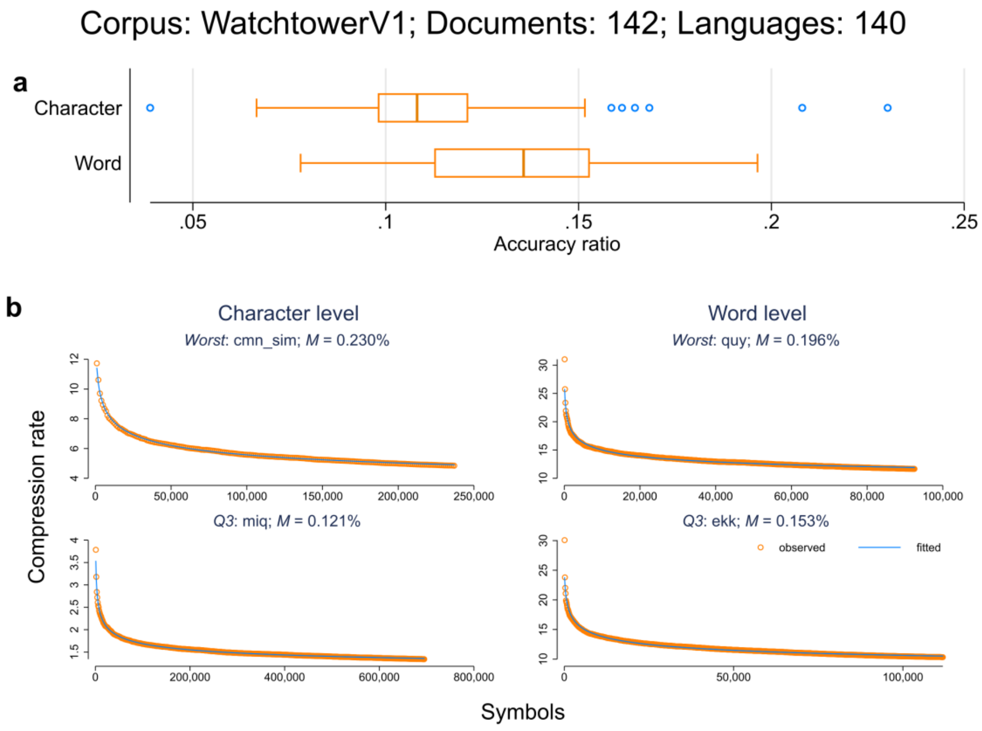

Supplementary Figure 6 | Corpus – WatchtowerV1. Cf. Supplementary Figure 4 for a description of the content.

S3.3.4 WatchtowerV2 (parallel)

In addition to the WatchtowerV1 version (cf. S3.3.3), we discovered that there are 93 verses that are available for 265 translations in 260 unique languages. All verses belong to a brochure entitled "*What Does God Require of Us?*" As for the WatchtowerV1 version, the verse order for every translation is randomly shuffled in a fully parallel way. With a chunk size of 5 on the level of words and 50 on the level of characters, we compressed a total of 570,764 different substrings. Supplementary Figure 7 visualizes the model fits.

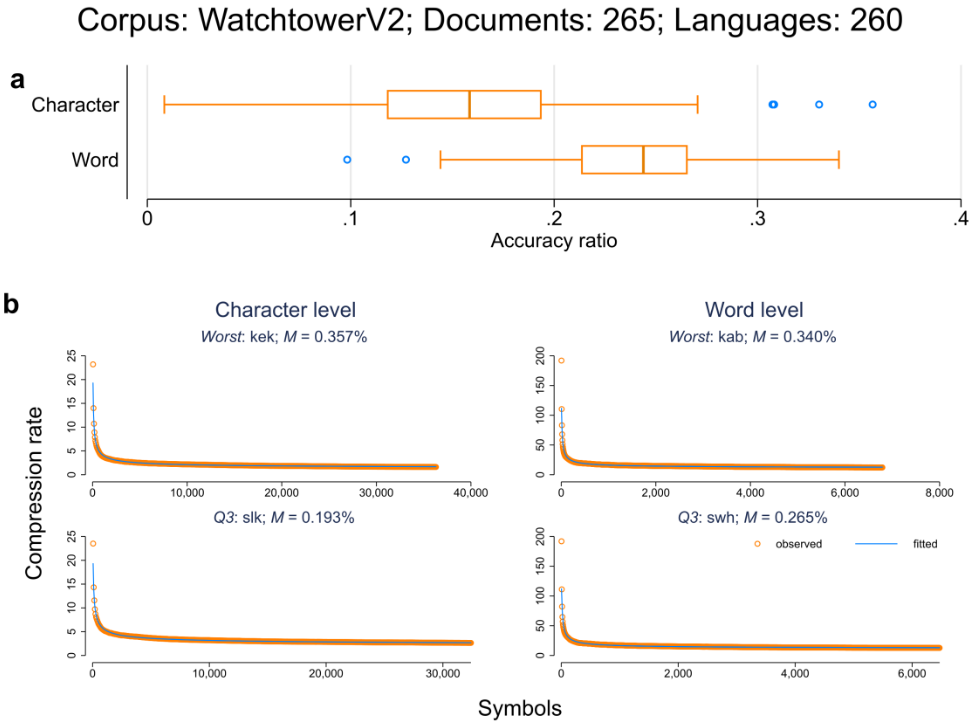

Supplementary Figure 7 | Corpus – WatchtowerV2. Cf. Supplementary Figure 4 for a description of the content.

S3.3.5 Quran (parallel)

We downloaded 43 different Quran translations from http://tanzil.net/trans/. The verse order for every translation is randomly shuffled in a fully parallel way. With a chunk size of 100 on the level of words and 1,000 on the level of characters, we compressed a total of 119,947 different substrings. Supplementary Figure 8 visualizes the model fits.

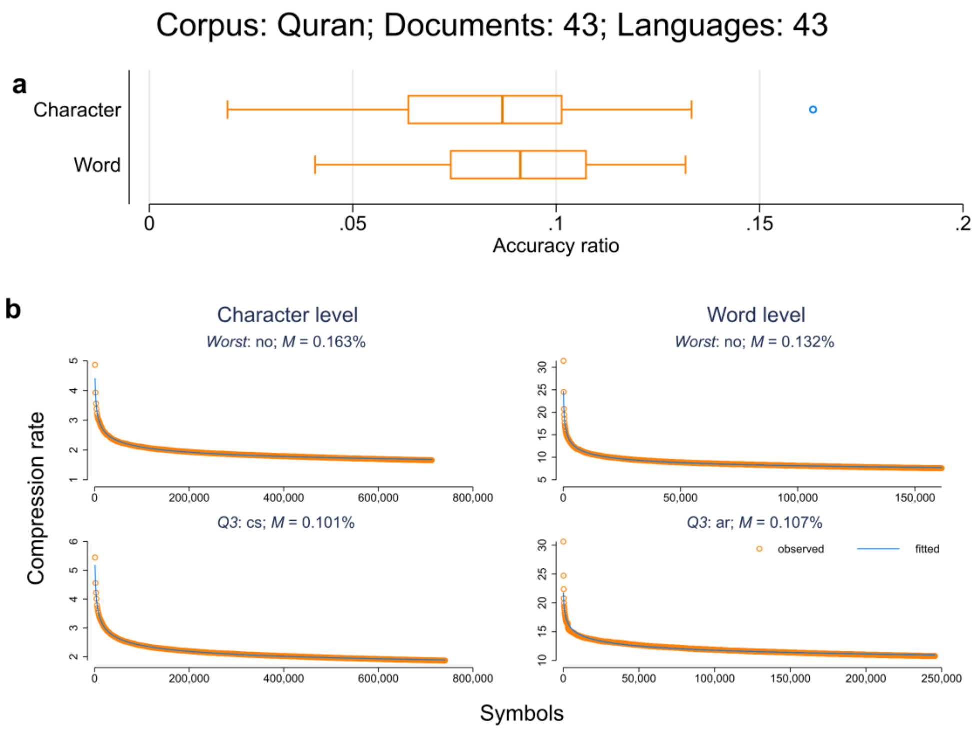

Supplementary Figure 8 | Corpus – Quran. Cf. Supplementary Figure 4 for a description of the content.

S3.4 News/Web/Wikipedia crawls

S3.4.1 GlobalVoices (comparable)

According to Wikipedia, the NGO Global Voices "is an international community of writers, bloggers and digital activists that aim to translate and report on what is being said in citizen media worldwide." (https://en.wikipedia.org/wiki/Global_Voices_(NGO)). Raw text files of all articles in 46 different languages were downloaded from http://casmacat.eu/corpus/global-voices.html (version: 2018Q4). Of those 46 languages, only languages with at least 1,000 lines were kept. For the resulting 40 languages, we randomly sampled 1,000 sentences per language.

With a chunk size of 10 on the level of words and 100 on the level of characters, we compressed a total of 123,792 different substrings. Supplementary Figure 9 visualizes the model fits.

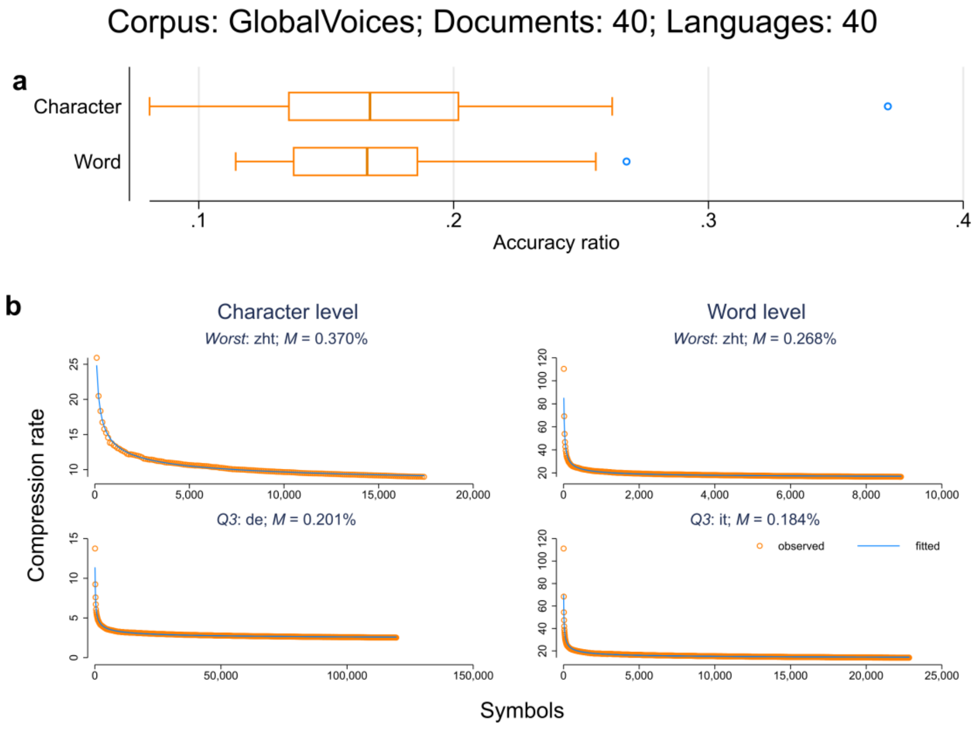

Supplementary Figure 9 | Corpus – GlobalVoices. Cf. Supplementary Figure 4 for a description of the content.

S3.4.2 LCCnews/ LCCweb/ LCCwiki (comparable)

Plain text files were downloaded from https://wortschatz.uni-leipzig.de/en/download. The Leipzig Corpora Collection (*LCC*) presents corpora in the same format from comparable sources. Here we focus on three collections that we name as follows (i) LCC_{news}, i.e. text material of crawled newspapers available online, (ii) LCC_{web}, i.e. text material crawled from randomly chosen web pages, and (iii) $LCC_{wikipedia}$, i.e. text material from Wikipedia dumps. For each corpus and for each available language we downloaded all texts files ending with "_10k-sentences" each consisting of 10,000 randomly shuffled sentences.[7] In total LCC_{news} consists of 112 different texts, LCC_{web} consists of 87 texts and $LCC_{wikipedia}$ consists of 171 texts.

With a chunk size of 1,000 on the level of words and 10,000 on the level of characters, we compressed a total of 112,618 different substrings. Supplementary Figure 10 visualizes the model fits.

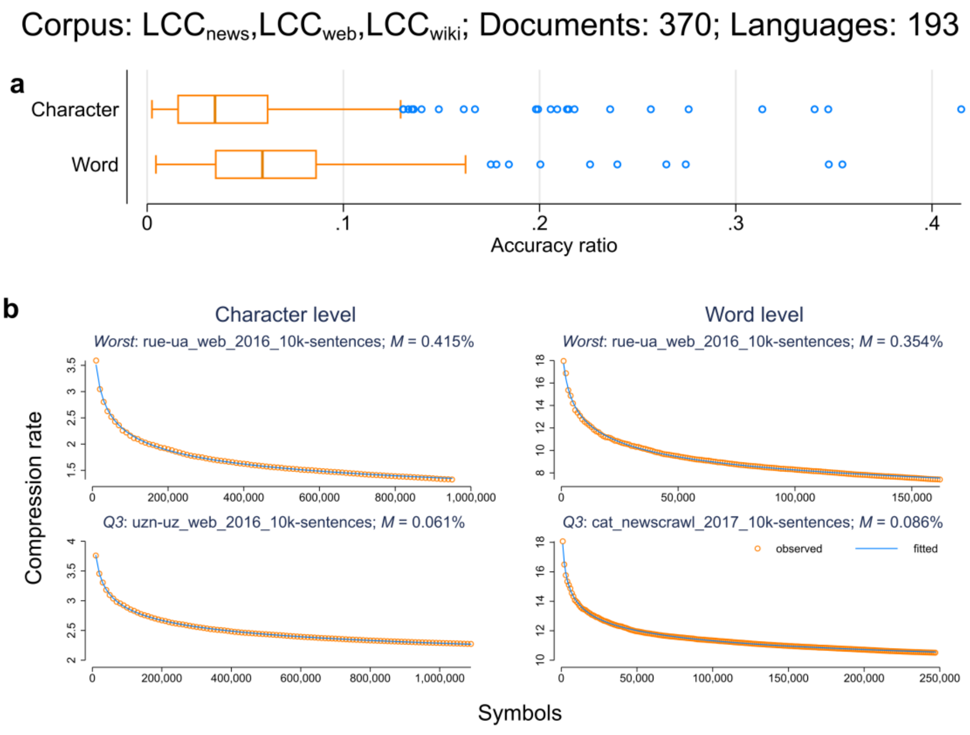

Supplementary Figure 10 | Corpus – LCC. Cf. Supplementary Figure 4 for a description of the content.

[7] When there was more than one text file available for a given language, we downloaded the newest one. Around 50% of the texts come from the 2016 version.

S3.5 Legalese texts

S3.5.1 UDHR (parallel)

We downloaded a total of 452 parallel translations of the Universal Declaration of Human Rights (*UDHR*) from https://unicode.org/udhr/. With a chunk size of 10 on the level of words and 100 on the level of characters, we compressed a total of 142,027 different substrings. Supplementary Figure 11 visualizes the model fits.

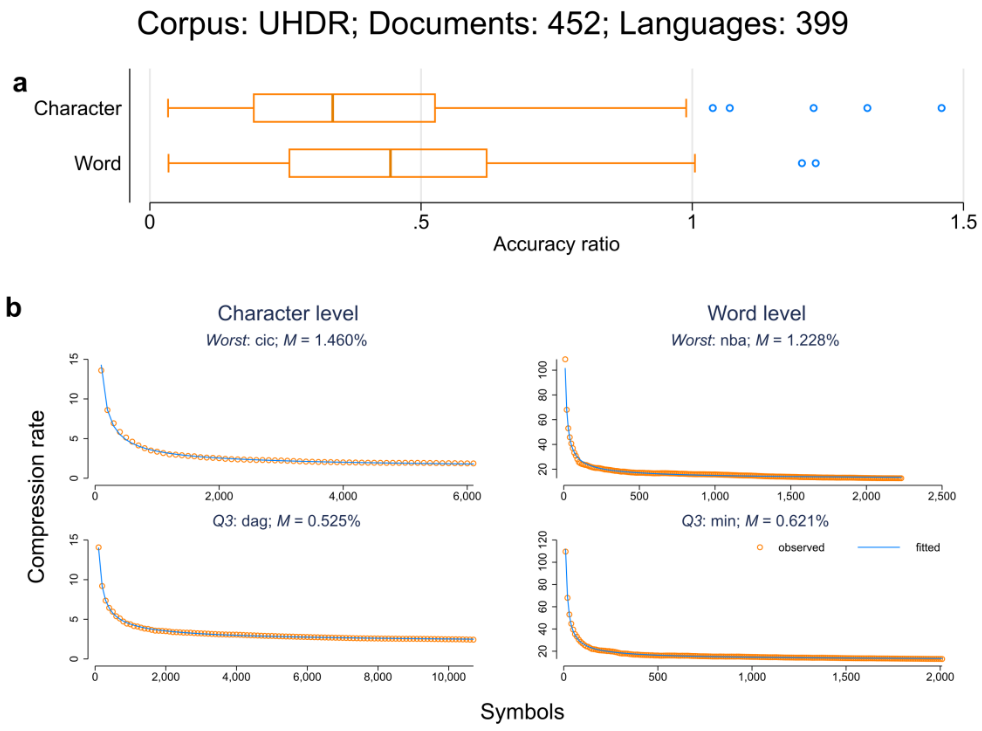

Supplementary Figure 11 | Corpus – UDHR. Cf. Supplementary Figure 4 for a description of the content.

S3.5.2 EUconst (parallel)

Parallel data in Unicode plain text format was downloaded from http://opus.nlpl.eu/EUconst.php, i.e. a parallel corpus collected from the European Constitution. We downloaded all parallelly aligned sentence-pairs for two languages (Tiedemann 2012) where the second language is Czech ("cs" *ISO*-639-2). We then used the Czech sentences as the key variable to align all 21 available languages, i.e. we pivoted via Czech (Aulamo et al. 2020). In total, we extracted 4,944 sentences that are available in all translations.[8] The sentence order for every translation is randomly shuffled in a fully parallel way. With a chunk size of 100 on the level of words and 1,000 on the level of characters, we compressed a total of 33,004 different substrings. Supplementary Figure 12 visualizes the model fits.

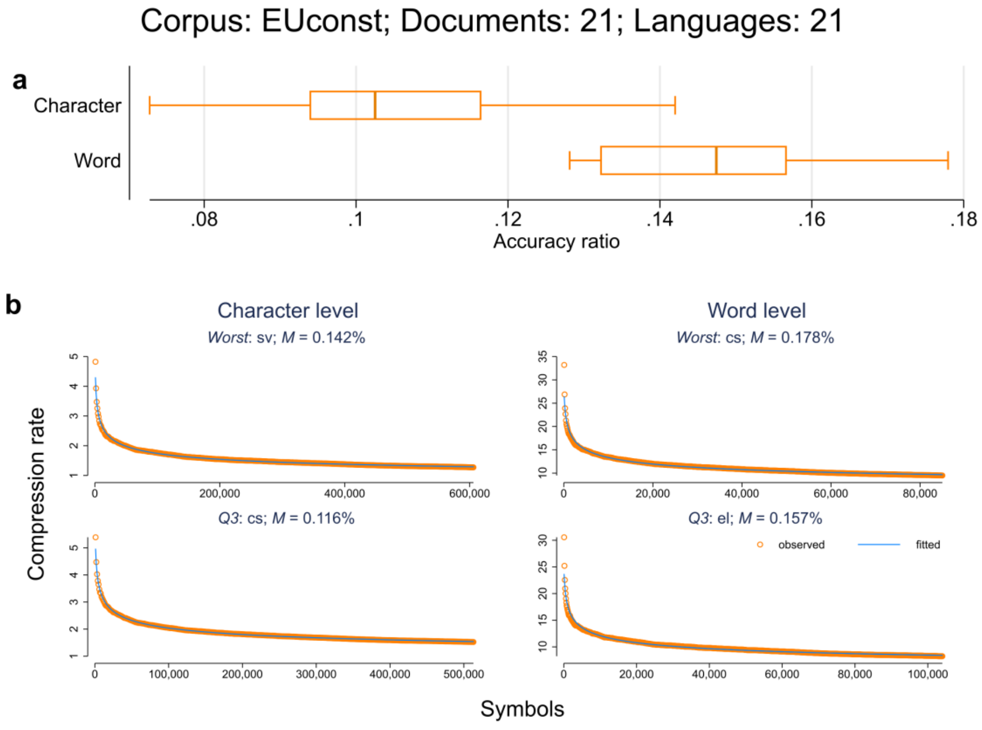

Supplementary Figure 12 | Corpus – EUconst. Cf. Supplementary Figure 4 for a description of the content.

[8] Duplicate lines were removed.

S3.5.3 Europarl (parallel)

Parallel data in Unicode plain text format was downloaded from http://opus.nlpl.eu/Europarl.php, i.e. a parallel corpus extracted from the European Parliament web site by Philipp Koehn (http://www.statmt.org/europarl/). We downloaded all parallelly aligned sentence-pairs for two languages (Tiedemann 2012) where the second language is Bulgarian ("bg" *ISO*-639-2).We then used the Bulgarian sentences as the key variable to align all 21 available languages. In total, we extracted 192,735 sentences that are available in all translations.[9] The sentence order for every translation is randomly shuffled in a fully parallel way. With a chunk size of 1,000 on the level of words and 10,000 on the level of characters, we compressed a total of 175,852 different substrings. Supplementary Figure 13 visualizes the model fits.

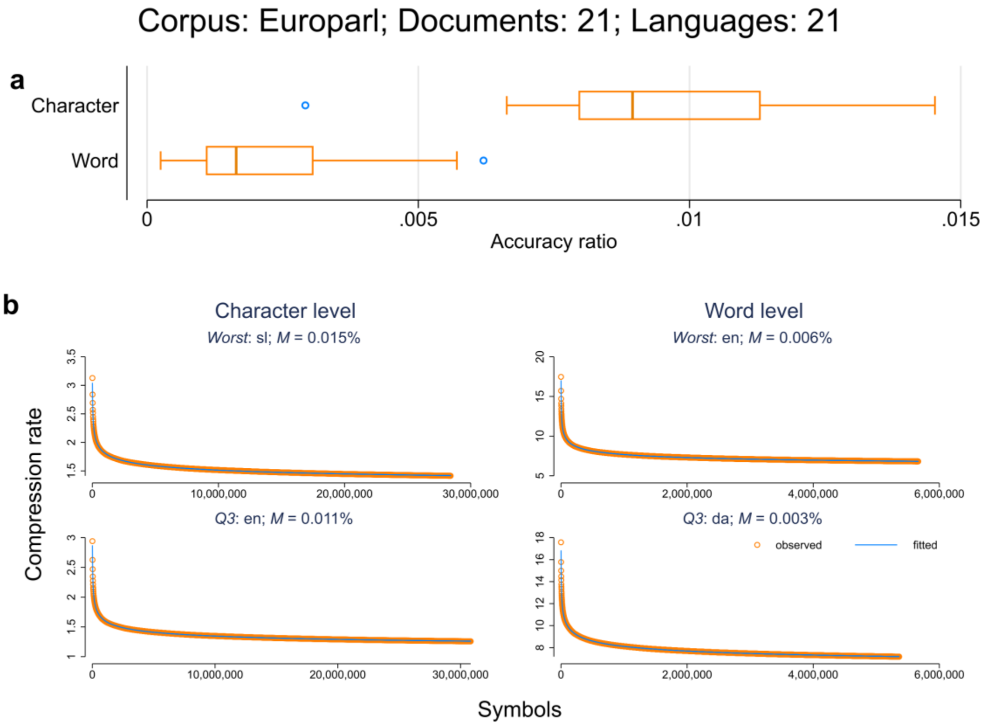

Supplementary Figure 13 | Corpus – Europarl. Cf. Supplementary Figure 4 for a description of the content.

[9] Duplicate lines were removed.

S3.5.4 EUmed (parallel)

Parallel data in Unicode plain text format was downloaded from http://opus.nlpl.eu/EMEA.php, i.e. a parallel corpus made out of PDF documents from the European Medicines Agency that "is an agency of the European Union in charge of the evaluation and supervision of medicinal products" (https://en.wikipedia.org/wiki/European_Medicines_Agency). The texts contain a lot of information on the prescription and (therapeutic) use of drugs, e.g. "if serum ferritin falls consistently below 500 µg/ l, an interruption of treatment should be considered (see section 4.4)".[10] We downloaded all parallelly aligned sentence-pairs for two languages (Tiedemann 2012) where the second language is Bulgarian ("bg" *ISO*-639-2).We then used the Bulgarian sentences as the key variable to align all 22 available languages. In total, we extracted 174,227 sentences that are available in all translations.[11] The sentence order for every translation is randomly shuffled in a fully parallel way. With a chunk size of 1,000 on the level of words and 10,000 on the level of characters, we compressed a total of 112,669 different substrings. Supplementary Figure 14 visualizes the model fits.[12]

---

[10] A sentence from the English data.

[11] Duplicate lines were removed.

[12] Regarding *h*, the results for this corpus are a clear outlier with a median value of 0.093 on the level of characters and 0.610 on the level of words compared to 1.266 and 7.688 for all other corpora. However, the texts contain a lot of standardized formulaic and repetitive phrases, e.g. "read the package leaflet" occurs 165 times and "can be taken with or without food" occurs 90 times in the English version. Therefore, such a small value for an extrapolation to infinity seems to make perfect sense.

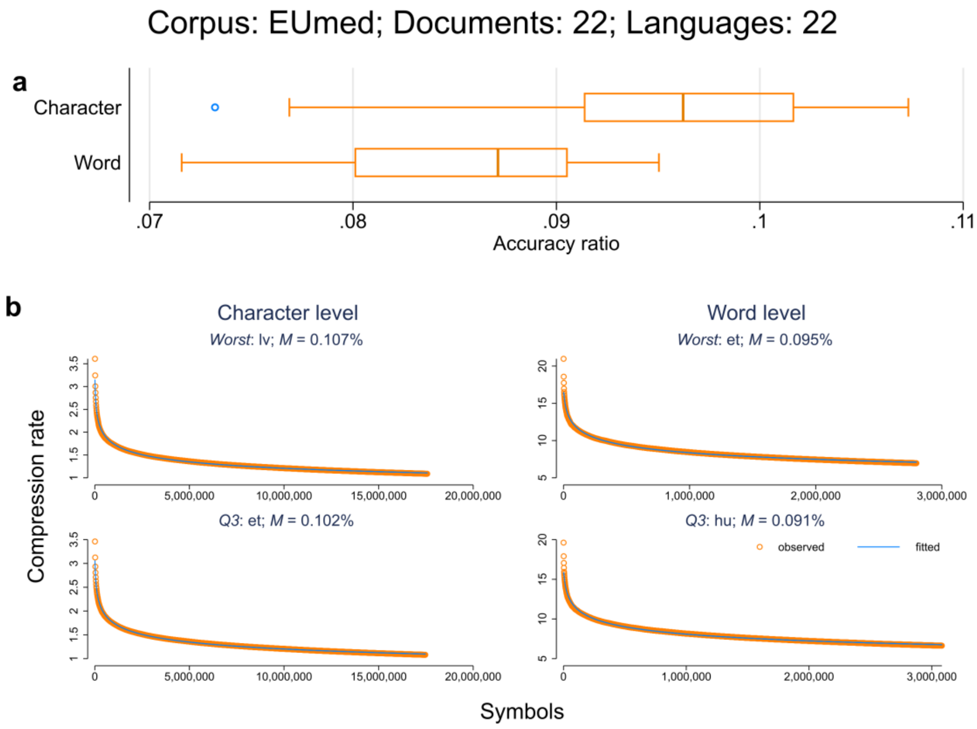

Supplementary Figure 14 | Corpus – EUmed. Cf. Supplementary Figure 4 for a description of the content.

S3.5.5 UNPC (parallel)

Parallel data in Unicode plain text format was downloaded from http://opus.nlpl.eu/UNPC.php (Source: https://cms.unov.org/UNCorpus), i.e. a parallel corpus compiled by (Ziemski, Junczys-Dowmunt & Pouliquen 2016) that is "composed from United Nations documents published by the original data creator. The parallel corpus presented consists of manually translated UN documents from the last 25 years (1990 to 2014)" (Ziemski, Junczys-Dowmunt & Pouliquen 2016). We downloaded all parallelly aligned sentence-pairs for two languages (Tiedemann 2012) where the second language is Arabic ("ar" *ISO*-639-2). We then used the Arabic sentences as the key variable to align all 6 available languages. We initially extracted 12,303,784 sentences that are available in all translations.[13] The sentence order for every translation is randomly shuffled in a fully parallel way. Since every word type is replaced by one unique Unicode symbol (cf. S3.1) and because there are only 1,112,064 valid Unicode symbols, preliminary analyses indicated that we could only keep the first 11,073,405 sentences on the level of words. Since the software package that we used to prepare the textual data (Stata 14.2) can only store strings up to 2,045 bytes and because that limited was reached after the first 4,723,010 sentences for the Russian data on the level of characters, we truncated the texts of the other five available languages at this point, too.

Further preliminary analyses revealed that due to the text length, the memory limit of the *PPM*d model (2 gigabytes) was reached at some point for all but one text. At this point, the tree in which the contextual statistics are stored is discarded and rebuilt from scratch (Mahoney 2013). To avoid this, we manually selected the maximum possible 7-zip *PPM*d order (cf. S3.1) for which the model memory limit was not reached per document and symbol type.

Again due to the text length, we compressed the texts every 50,000 symbols for the first 1,000,000 symbols, and then compressed the rest of the texts every 500,000 symbols on both the character and the word level. In total, we compressed a total of 13,919 different substrings. Supplementary Figure 15 visualizes the model fits.[14]

---

[13] Duplicate lines were removed.

[14] *H* was only calculated on the level of words for this corpus.

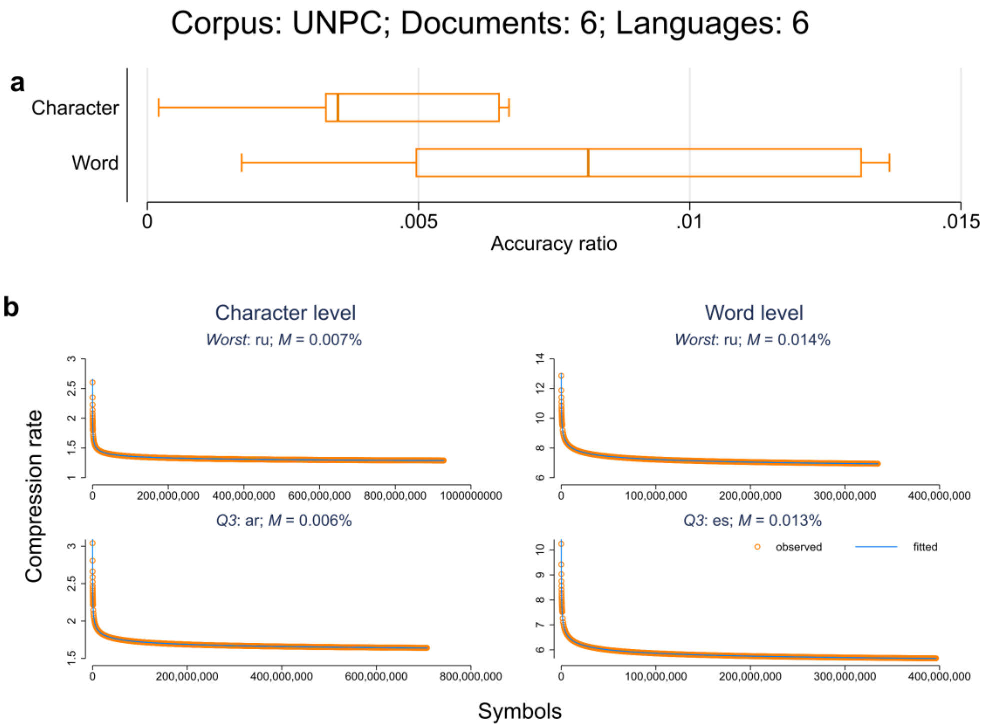

Supplementary Figure 15 | Corpus – UNPC. Cf. Supplementary Figure 4 for a description of the content.

S3.6 Subtitles

S3.6.1 MSub01 – MSub13 (parallel)

The data were downloaded from https://github.com/levshina/ParTy-1.0. The ParTy corpus (Levshina 2016) contains subtitles for the following thirteen movies:

- MSub01: Amelie
- MSub02: Avatar
- MSub03: Black Swan
- MSub04: Bridge of Spies
- MSub05: Das Leben der Anderen
- MSub06: Frozen
- MSub07: Gone Girl
- MSub08: Grand Budapest Hotel
- MSub09: Imitation game
- MSub10: Inception
- MSub11: Ironlady
- MSub12: Noah
- MSub13: Spectre

According to http://www.natalialevshina.com/corpus.html, all files were downloaded from the online repositories opensubtitles.org, subscene.com and ted2srt.org and aligned automatically. Each sentence for each document was randomly shuffled. In total, we used 269 documents in 43 different languages. With a chunk size of 10 on the level of words and 100 on the level of characters, we compressed a total of 336,250 different substrings. Supplementary Figure 16 visualizes the model fits.

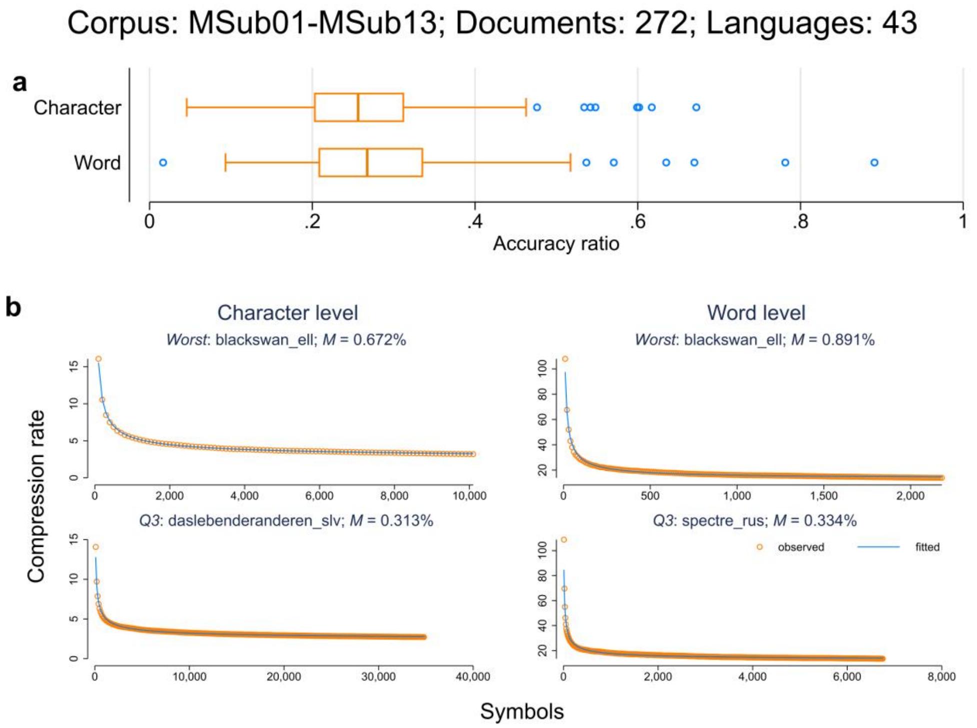

Supplementary Figure 16 | Corpus – MSub01 – MSub13. Cf. Supplementary Figure 4 for a description of the content.

S3.6.2 TEDt01 – TEDt09 (parallel)

TED is a media organization that posts short talks "online for free distribution under the slogan 'ideas worth spreading'" (https://en.wikipedia.org/wiki/TED_(conference)). In a collaborative effort, “TED talks” also offers subtitles by using the subtitling platform https://amara.org/en/. We downloaded plain text data from https://amara.org/en/teams/ted/videos/ for talks that have (i) the largest number of completed subtitles in different languages and (ii) are reasonably long. We selected the following nine talks:

- TEDt01: Bring on the learning revolution
- TEDt02: Do schools kill creativity
- TEDt03: Doing the impossible cutting through fear Dan Meyer TEDxMaastricht
- TEDt04: Modern Warrior Damien Mander at TEDxSydney
- TEDt05: My philosophy for a happy life Sam Berns at TEDxMidAtlantic
- TEDt06: Secondary sugar kills Laurent Adamowicz TEDxYouthBeaconStreet
- TEDt07: Speak to the heart Marleen Laschet TEDxTrondheim
- TEDt08: Success is a continuous journey
- TEDt09: Why is x the unknown

In total, we used 436 documents in 80 different languages. With a chunk size of 5 on the level of words and 50 on the level of characters, we compressed a total of 271,913 different substrings. Supplementary Figure 17 visualizes the model fits.

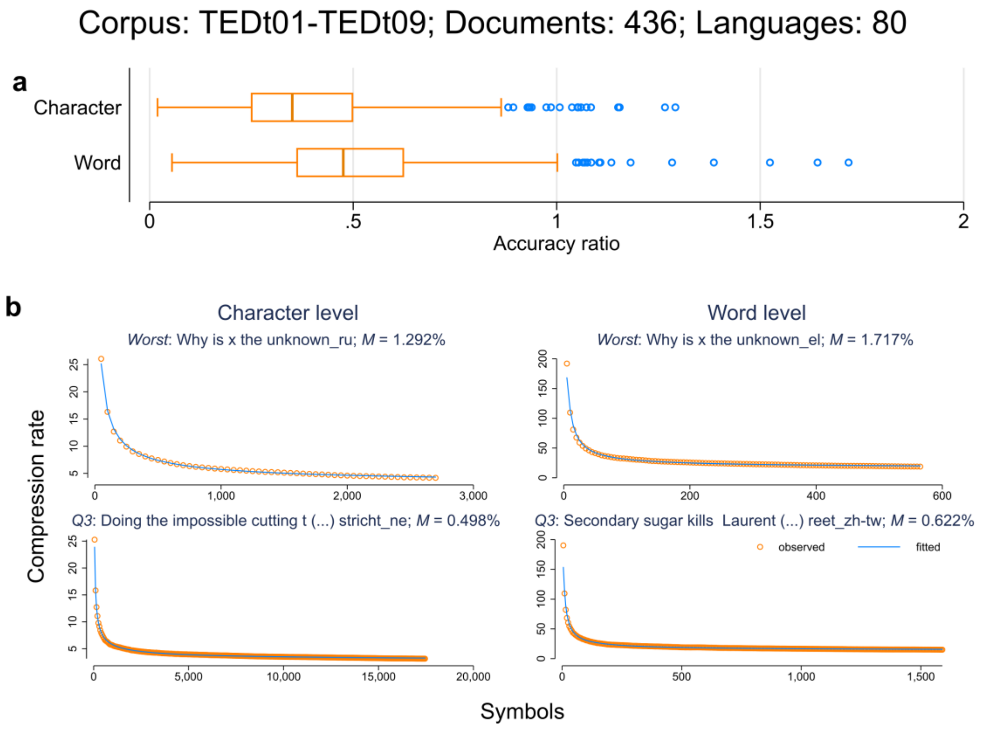

Supplementary Figure 17 | Corpus – TEDt01 – TED09. Cf. Supplementary Figure 4 for a description of the content.

S3.7 Other

S3.7.1 Ubuntu (comparable)

Parallel data in Unicode plain text format was downloaded from http://opus.nlpl.eu/Ubuntu.php, a corpus of Ubuntu localization files (source: https://translations.launchpad.net), containing translations for sentences and phrases displayed by computer applications, such as "allow packages from unauthenticated sources".[15] We downloaded all parallelly aligned sentence-pairs for two languages (Tiedemann 2012) where the second language is English ("en" *ISO*-639-2).[16]
Of those 207 languages, only languages with at least 1,000 lines were kept. For the resulting 86 languages, we randomly sampled 1,000 sentences per language. The sentence order for every translation is randomly shuffled. In total, we used 86 documents in 86 different languages. With a chunk size of 5 on the level of words and 50 on the level of characters, we compressed a total of 162,127 different substrings. Supplementary Figure 17 visualizes the model fits.

[15] A sentence from the English data.

[16] We removed sentences that contained English terminology and sentences that contained the following symbols: -f -s -h -u -t -a -i -o : > / url } ubuntu checksum _ << % "nothing selected" string launchpad @ hhtp fonts insertinsert u+0025 palette script gnome server. In addition, "sentences" that consist of only one word and duplicate lines were removed.

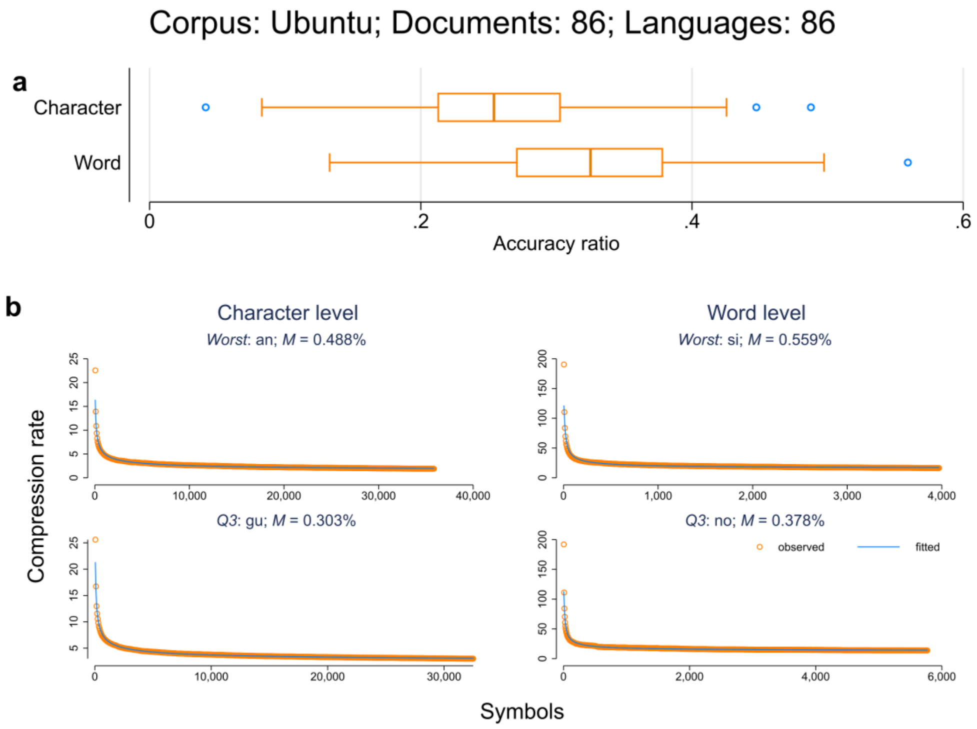

Supplementary Figure 18 | Corpus – Ubuntu. Cf. Supplementary Figure 4 for a description of the content.

S3.7.2 GoogleT (parallel)

We downloaded the following excerpt from the book "Aldono al la Dua Libro de l' Lingvo Internacia" in the constructed language Esperanto, written by its inventor L.L. Zamenhof (downloaded from http://esperanto.davidgsimpson.com/librejo/index.html on 09/04/2019):

"En mia unua libro mi petis ĉiujn amikojn de l' lingvo internacia esprimi ilian juĝon pri la lingvo, kiun mi proponis, montri al mi ĉiujn erarojn, kiujn ili trovis en ĝi, kaj ĉiujn plibonigojn, kiujn ili povas proponi, kaj helpi min tiel doni al la lingvo la plej bonan formon, ĉar la finan formon mi intencis doni al la "Lingvo Internacia" ne pli frue ol en la fino de l' jaro 1888, pripensinte kaj provinte antaŭe ĉiujn juĝojn kaj proponojn, kiuj estus senditaj al mi ĝis tiu tempo. En la "Dua Libro" mi diris, ke por fari la lingvon libera de ĉiuj personaj eraroj, estus dezirata, ke ia instruita societo prenu en siajn manojn la sorton de l' lingvo kaj, aŭskultinte la konsilojn de kompetentaj personoj, ĝi donu al la lingvo la finan formon, kiu estus egale ordona por mi, kiel por ĉiu alia amiko de l' lingvo internacia. Nun mi kun la plej granda ĝojo povas sciigi ĉiujn amikojn de l' lingvo internacia, ke mia deziro ne restis vana. Ankoraŭ en la fino de l' jaro 1887, t.e. ankoraŭ antaŭ la ricevo de mia libreto, la Amerika Filozofia Societo en Filadelfio (The American Philosophical Society) elektis komitaton por pripensi kaj decidi la demandon, ĉu lingvo internacia estas necesa, ĉu ĝi estas kreebla, kaj kiel ĝi devas esti. La frukto de l' laboroj de la komitato estis jena decido: ke lingvo internacia estas kreebla, ke ĝi estas necesa, ke ĝi devas havi gramatikon la plej simplan kaj naturan, kun la plej simpla ortografio, kaj fonologio, kaj la vortoj devas esti agrablaj por la orelo; ke la vortaro devas esti kreita el vortoj pli malpli rekoneblaj por la plej gravaj civilizitaj popoloj; ke la fina formo de tia lingvo devas esti la frukto de l' laboroj ne de unu persono, sed de la tuta instruita mondo. Sur la fondo de ĉio supre dirita, la "Amerika Filozofia Societo" decidis dissendi al ĉiuj instruitaj societoj la proponon fari internacian kongreson de instruituloj por decidi la finan formon de lingvo tutmonda. Tiel la leganto vidas, ke ne sciante ankoraŭ pri mia laboro, la "Amerika Filozofia Societo" venis al tiuj samaj decidoj pri lingvo tutmonda, al kiuj mi venis, kaj ke la principoj, kiujn la "Amer. Fil. Societo" ellaboris por la lingvo teorie, estas pli malpli egalaj al tiuj, kiujn mi efektivigis praktike. Tial ĝi estas tute natura, ke ricevinte mian libreton jam en la fino de siaj laboroj, la komitato trovis, ke mia lingvo estas sufiĉe proksima al la idealo, kiun ĝi ellaboris teorie. Jen kion diras pri la "Lingvo internacia" sinjoro Henry Phillips, Jr (unu el la tri personoj, el kiuj estis farita la komitato por decidi la demandon pri lingvo tutmonda): "La plej nova propono al la publiko kaj ĝis nun la plej simpla kaj la plej racionala, estas la "Lingvo internacia," kreita de d-ro S* el Varsovio. La principoj, sur kiuj ĝi estas fondita, estas en la tuto maleraraj; ĝia vortaro ne estas kreita laŭ la persona volo kaj juĝo de l' aŭtoro, sed prenita el la lingvoj franca, germana kaj angla kaj en parto el la latina, kaj ĝi enhavas la vortojn, kiuj estas similaj en tiuj lingvoj; estas faritaj kelkaj ŝanĝoj pro la bonsoneco. Pro tio kaj pro ĝia gramatiko la lingvo estas mirinde facila por lerni, prezentante neniajn el la kalejdoskopaj rompaĵoj kaj ŝiraĵoj de la Volapük'. La gramatiko de tiu lingvo estas el la plej simplaj, tiel simpla, kiel en nia propra lingvo, kaj la reguloj por la kreado de vortoj estas tiel klaraj kaj tiel facilaj, ke la vortaro el radikvortoj povas esti farita tre malgranda ..." Rakontinte mallonge la tutan konstruon de l' "Lingvo internacia" kaj ĝian gramatikon, kaj montrinte kelkajn punktojn, kiuj laŭ lia juĝo devus esti ŝanĝitaj, sinjoro H. Ph. finas: "D-ro S*, kiu skribas sub la nomo de d-ro Esperanto, estas tre modesta en siaj postuloj kaj proponas sian lingvon al la publika kritiko tra la tempo de unu jaro, antaŭ ol li donos al ĝi la finan formon. Post tiu fina trarigardo kaj ŝanĝo li volas prezenti ĝin por la publika uzado. Li petas siajn legantojn promesi lerni la lingvon nur tiam, se 10,000,000 personoj estos donintaj tian saman promeson."

We then machine-translated this excerpt using https://translate.google.com on 09/04/2019 into all available languages.[17] In total, we translated the excerpt into 102 different languages. With a chunk size of 5 on the level of words and 50 on the level of characters, we compressed a total of 22,643 different substrings. Supplementary Figure 19 visualizes the model fits.

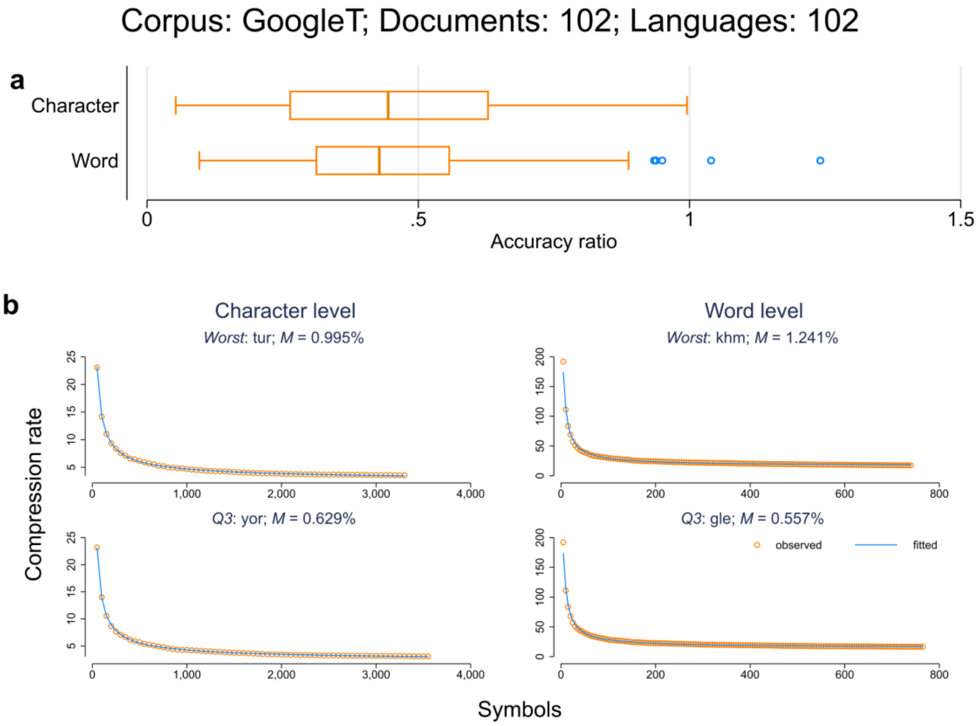

Supplementary Figure 19 | Corpus – GoogleT. Cf. Supplementary Figure 4 for a description of the content.

[17]We classified "Arabic" as *ISO* "arb", "Chinese" as "cmn", "Yiddish" as "ydd" and "Punjabi" as "pan".

S3.7.3 TatoebaV1 (comparable)

According to Wikipedia, "Tatoeba is a free collaborative online database of example sentences geared towards foreign language learners" (https://en.wikipedia.org/wiki/Tatoeba). Raw data for 340 different languages were downloaded from https://tatoeba.org/deu/downloads. An example sentence in the constructed language Toki Pona is "kulupu ante pi soweli Kane li mute."[18] For this version (V1), we only kept languages with at least 100 available sentences. For the resulting 183 languages, we randomly sampled 100 sentences per language. With a chunk size of 5 on the level of words and 50 on the level of characters, we compressed a total of 35,116 different substrings. Supplementary Figure 20 visualizes the model fits.

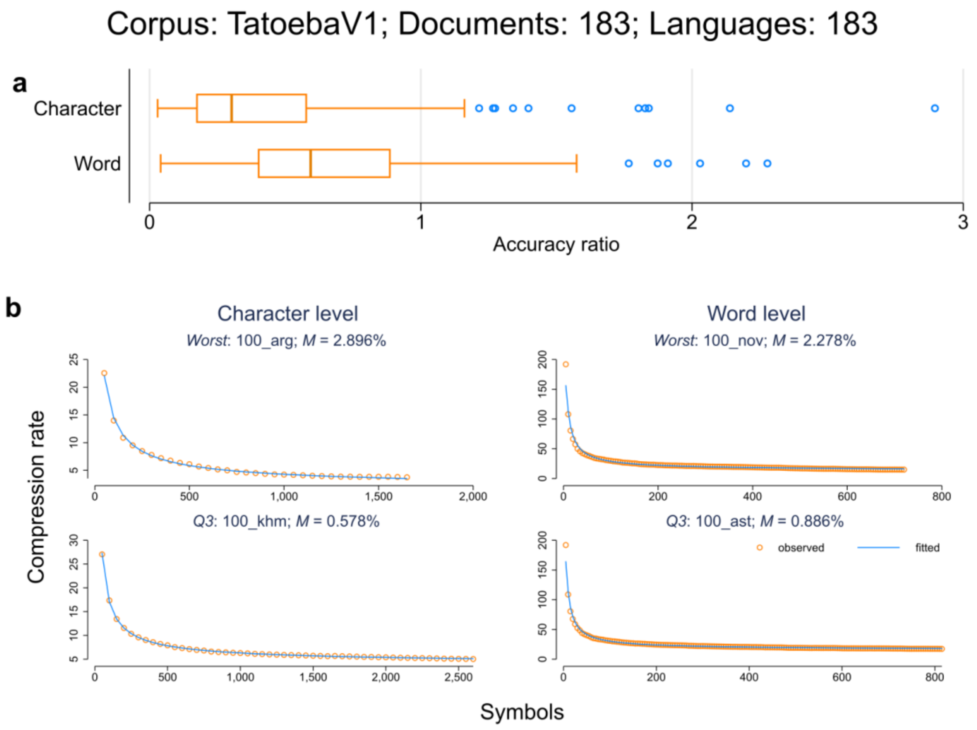

Supplementary Figure 20 | Corpus – TatoebaV1. Cf. Supplementary Figure 4 for a description of the content.

---

[18] The English translation in Tatoeba is as follows: "There are lots of different types of dogs." Since Toki Pona has no *ISO* code, we chose the (unassigned) ISO code "tok" to label it in our data.

S3.7.4 TatoebaV2 (comparable)

After generating the first version described in the last section, we used the remaining sentences to generate a second version.[19] Here we only kept languages with a least 500 available sentences. For the resulting 123 languages, we randomly sampled 500 sentences per language. With a chunk size of 5 on the level of words and 50 on the level of characters, we compressed a total of 124,128 different substrings. Supplementary Figure 21 visualizes the model fits.

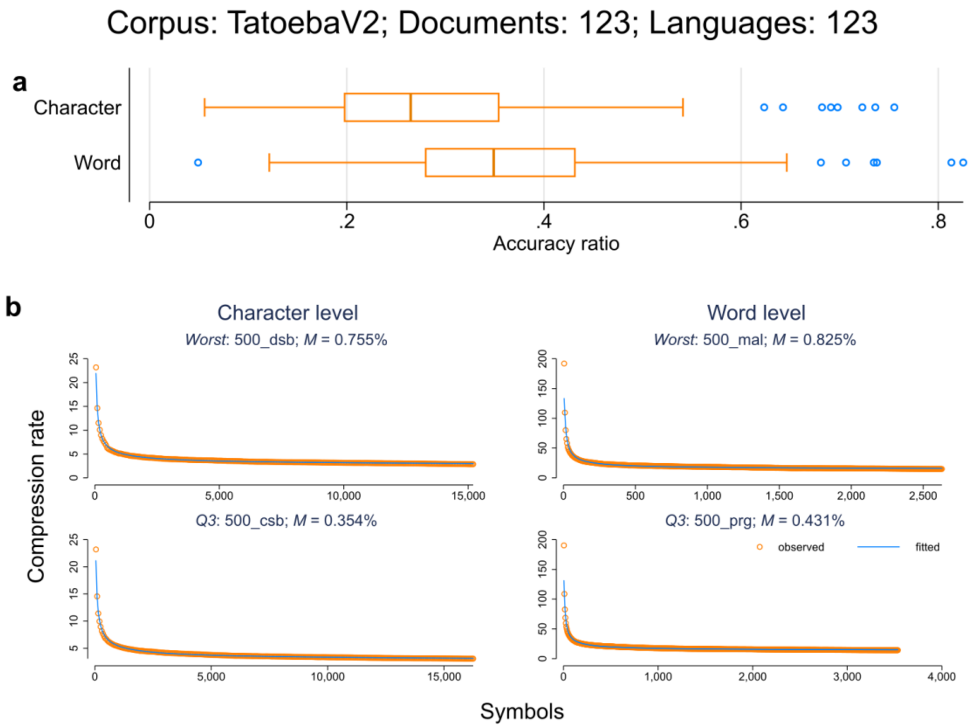

Supplementary Figure 21 | Corpus – TatoebaV2. Cf. Supplementary Figure 4 for a description of the content.

[19] Put differently, the sentences sampled for the V1 version were removed from the raw data.

S3.8 Wordlist: Crúbadán

The Crúbadán project aims at creating text corpora for a large number of (especially under-resourced) languages via web-crawling (Scannell 2007), often based on Bible translations or Wikipedia entries. Since no full text is provided, we downloaded all zip files by first generating lists of all available documents by extracting the document "writingsystems.csv" for all available scripts via http://crubadan.org/writingsystems.csv?sEcho=1&iSortingCols=1&iSortCol_0=4&sSortDir_0=asc&sSearch_4=`script' where "`script'" is replaced by one of the following scripts: Arab, Armn, Beng, Cans, Cher, Copt, Cyrl, Cyrs, Deva, Ethi, Geor, Glag, Grek, Gujr, Guru, Hans, Hant, Hebr, Jpan, Kali, Khmr, Knda, Kore, Laoo, Latg, Latn, Limb, Lisu, Mlym, Mong, Mymr, Nkoo, Ogam, Orya, Saur, Shaw, Sinh, Syrc, Taml, Telu, Tfng, Tglg, Thaa, Thai, Tibt, Vaii, Yiii. After aggregating the lists, we downloaded all zip files from http://crubadan.org/files/. We then kept all word frequency lists for which there is at least one available line of information in the document `bcp'-words.txt where "`bcp'" stands for an adjusted BCP-47 language code, e.g. "omw-x-aat" stands for the language "Aatasara" spoken in Papua New Guinea. In total, we arrived at 2,216 word frequency lists for a total of 1,943 different languages. Using the available word frequency information, we calculated the Gibbs-Shannon entropy (cf. Methods: equation (1)) on the word level. Since most frequency lists are only based on a very limited number of documents (simply due to the fact that for such languages those documents "may very well represent the totality of all electronic documents in existence" (Scannell 2007)[20]), we use both the text length and the number of documents as control variables in what follows. In addition, we noted that in 103 cases (4.65%), the available word frequency lists are truncated, i.e. word types with a frequency lower than some threshold level are excluded from the list.[21] Therefore, we generated an additional variable that

[20] For example, 37.5% of all available frequency lists are generated on the basis of one or two documents.

[21] For example, the word frequency list for English is generated based on 7,780 documents. The list is truncated at 2, so word types with a frequency of one are excluded. In total, 68 lists are truncated at 2, 30 lists are truncated at 3 and 5 lists are truncated at 4.

indicates whether the corresponding word frequency list is truncated (yes/no) and that variable then serves as a control in what follows.[22]

[22] Since the total number of words is listed on the An Crúbadán page, another possibility would have been to estimate the frequency mass of the excluded word types using basic properties of word frequency distributions (Baayen 2001). For example, in the case of a word list truncated at 2: if the total number of words listed on the An Crúbadán page were 2,000 and the word frequency sum in the corresponding list were 1,000, then we would immediately know that 1,000 word types with a frequency of 1 need to be added to the word frequency list. However, we noted that in 41 cases the word frequency sum for the available lists is greater than the frequency sum mentioned on the An Crúbadán page. Take for example the language Moose Cree: http://crubadan.org/languages/crm. Here, the listed word frequency sum ("available words") is 114,304, however, in the crm.zip data file, the sum amounts to 126,524. Therefore, we ultimately decided against this estimation strategy.

S4. Code and data

Code and data are available at https://osf.io/f5mke/. Speaker community size estimates and genealogical/geographical classifications are taken from Bentz et al. (2018) whose data are in turn based on data from *Ethnologue*, 17th edition (Simons & Fennig 2013) and *Glottolog* (Hammarström, Forkel & Haspelmath 2019). We thank Christian Bentz for additionally providing us with country information from *Ethnologue*, 17th edition (Simons & Fennig 2013), the last openly available version of the *Ethnologue* (Bentz et al. 2018).

Regarding potential caveats and problems of assigning environmental variables to individual languages in order to reflect local grouping structure, see Bentz et al. (2018) or Jaeger et al. (2011).

Writing system information was taken from the Crúbadán project (cf. S3.8, variable "script").

For the Crúbadán word frequency lists, the information as listed in the file writingsystems.csv was used (cf. S3.8). For all other documents, we used the variable "ISO" to prepare a list of all different writing systems per language. For languages with only one available writing system in the csv file, the corresponding writing system was directly assigned to each document in the corresponding language. For languages with more than one available writing system, we manually selected the correct writing system for each document. In addition, we manually selected writing systems for documents without available information.

Data preparation, management and analysis were done in Stata 14.2 (StataCorp 2015) and Stata 18 (StataCorp 2023). Commented code is available at https://osf.io/f5mke/, folder: Stata Code.[23]

A replication of the key results was done in R (R Core Team 2022), the corresponding scripts are available at https://osf.io/f5mke/, folder: R Code. Supplementary Table 5 lists and describes the variables included in the final dataset that is available as a csv file ("textcomp_data.csv") at https://osf.io/f5mke/, folder: Data.

---

[23] Individual do-files are organized in a separate do-file called "_master.do". This do-file can be used to re-create the whole project from scratch.

Supplementary Table 5: Description of the dataset that is available as a csv file as "textcomp_data.csv" at https://osf.io/f5mke/, folder: Data. 1st column: Variable name. 2nd column: Variable type (string/numeric). 3rd column: Description. 4th column: Value labels (if applicable).

Name	Type	Description	Values
corpus	string	Name of the corpus	cf. Supplementary Table 4; column 1
corpus_type	string	Type of source corpus	parallel, comparable, word frequency list
version	string	version for which the quantities *r*, *A*, *h*, *b* and *H* were calculated (cf. section S4)	full, truncated
type	string	Symbolic level: "char_level" for characters, "word_level" for words, "BPE" for byte pair encoding (only for BibleOT)	char_level, word_level, BPE
script	string	Writing system	4-letter code
ISO	string	Language specific *ISO 639-3* code	3-letter code
docname	string	Name of the document for which the quantities are calculated	-
language_name	string	Name of the language	-
family	string	Name of the language family	-
macroarea	string	Name of the macro-area	-
country	string	Name of the country	-
r_c	numeric	Compression rate (denoted as *r* in the text)	-
h_unigram	numeric	Gibbs-Shannon entropy (cf. Methods: equation (1), denoted as *H* in the text)	-
h_extrapol	numeric	Estimated extrapolated entropy rate (cf. Methods: equation (5) and equation (6), denoted as *h* in the text)	-
A	numeric	Proportionality constant from the *ansatz* function (cf. Methods: equation (5))	-
b	numeric	Learning difficulty (cf. Methods: equation (5), denoted as *b* in the text).	-
length	numeric	Length of the document in symbols	-
N_chunks	numeric	Number of compressed substrings of the document	-
N_docs	numeric	Number of crawled documents for which the corresponding word list in the Crúbadán data was generated (cf. S3.8)	-
trunc_yes	numeric	Binary indicator indicating if the corresponding word list in the Crúbadán data is truncated (1) or not (0) (cf. section S3.8)	0, 1
ppmd_order	numeric	Order of the 7-zip *PPM*d model	-
q3_r_c	numeric	Compression rate for the first ≈ 25% of the document (see section S8)	-
q2_r_c	numeric	Compression rate for the first ≈ 50% of the document (see section S8)	-
q1_r_c	numeric	Compression rate for the first ≈ 75% of the document (see section S8)	-
iterations	numeric	Number of iterations needed for Methods: equation (6) to converge	-
acc_ratio	numeric	Accuracy ratio (cf. Methods: equation (8)) in %	-
Pop	numeric	Speaker population size (cf. section S4)	-
latitude	numeric	Latitude (cf. section S4)	-
longitude	numeric	Longitude (cf. section S4)	-
log_pop	numeric	Speaker population size (logged)	-

log_docs	numeric	Number of crawled documents (logged) for which the corresponding word list in the Crúbadán data was generated (cf. S3.8)	-
log_length	numeric	Text length (logged) based on which the corresponding word list in the Crúbadán data was generated (cf. section S3.8)	-

Regarding the variable "version": there are two versions, (i) a full version and (ii) a truncated version for all corpora expect for the Crúbadán word frequency lists.[24] For version (i), quantities *r*, *A*, *h*, *b* and *H* were calculated for the whole corresponding text. As written in the main part of the paper, most, if not all, quantities in the context of word frequency distributions vary systematically with the text length (Baayen 2001; Tweedie & Baayen 1998; Koplenig, Wolfer & Müller-Spitzer 2019). As a check, we therefore prepared a truncated version (ii) by first computing $ƛ_c$ that denotes the minimum length in symbols per corpus and per type. We then calculated the aforementioned quantities based on the first $ƛ_c$ symbols for each available text.

[24] Corresponding cells are empty.

S5. Coverage

Ethnologue (Simons & Fennig 2017) list 7,099 different living languages. Aggregating all native speakers (variable "L1_Users") for all languages for which we have available data (excluding macro-languages) and dividing the resulting quantity by the number of native speakers for all languages (calculated as the sum of the "L1_Users" variable) results in a coverage of 90.72%. Around 54% of all languages (3,866) have a standardized written representation. Excluding macro-languages again, we have available information for 45.86% of those languages.

S6. Correlation matrix

To generate the correlation matrix for Figure 3 all quantities were first averaged by corpus and symbol type for languages with more than one available document per corresponding corpus. We considered the three following variables: h, r and N, i.e. the length in symbols. In addition, we also used information from the Crúbadán project that is available for a large number of (especially under-resourced) languages (cf. S3.8). Because no full texts are available, we estimated Gibbs-Shannon entropy (cf. Methods: equation (1)) on the level of words based on the available word frequency information and call the resulting variable $H_{Crubadan}$.

For all corpus pairs and both within and across both symbolic levels, we calculated Spearman correlation coefficients between all combinations of variables, corpora and symbol types where each observation is one language as identified by the corresponding *ISO* code. All correlations were then stored in a matrix.[25]

[25] In total, we have 2 symbolic levels (words/characters) × 3 variables (h, r and L) × 40 corpora = 240 variables + $H_{Crubadan}$ = 241 variables.

S7. Relative standard deviations for *h* and *r*

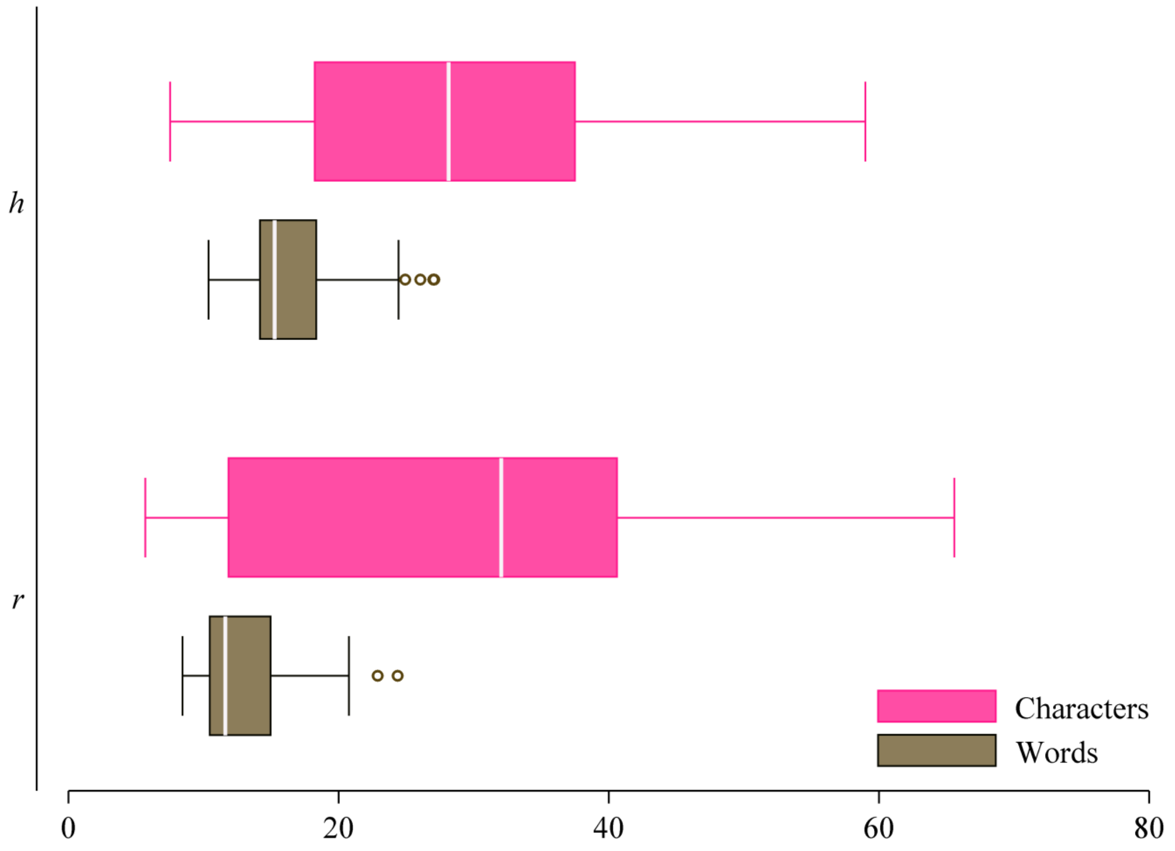

Supplementary Figure 22 | Relative standard deviations (*SD*) for entropy rates ("*h*") and compression rates ("*r*") are calculated per corpus and symbol type as the ratio of the standard deviation to the mean (in %). The figure shows that the variability in *h* is smaller for words than for characters. In 65% of all 40 corpora, the relative *SD* for *h* is lower for words than for characters. If we only look at the 50% of corpora with the most available documents (at least 38 individual documents per corpus), then 75% of those 20 corpora display a lower relative *SD* for words. For *r* the corresponding percentages are 70% of all corpora and 90% for the 20 largest ones (in terms of available documents).

S8. Correlations between entropy rates and learning difficulty

Our ansatz function has three parameters (see equations (5) – (7)): the limiting entropy rate *h*, a proportionality constant and an exponent *b*. While *h* quantifies how difficult it is to predict, *b* quantifies how difficult it is to learn to predict, as aptly put by Takahira et al. (2016): lower *b*-values are indicative of slower convergence, i.e. learning is more difficult (see Supplementary Figure 3 for an illustration).

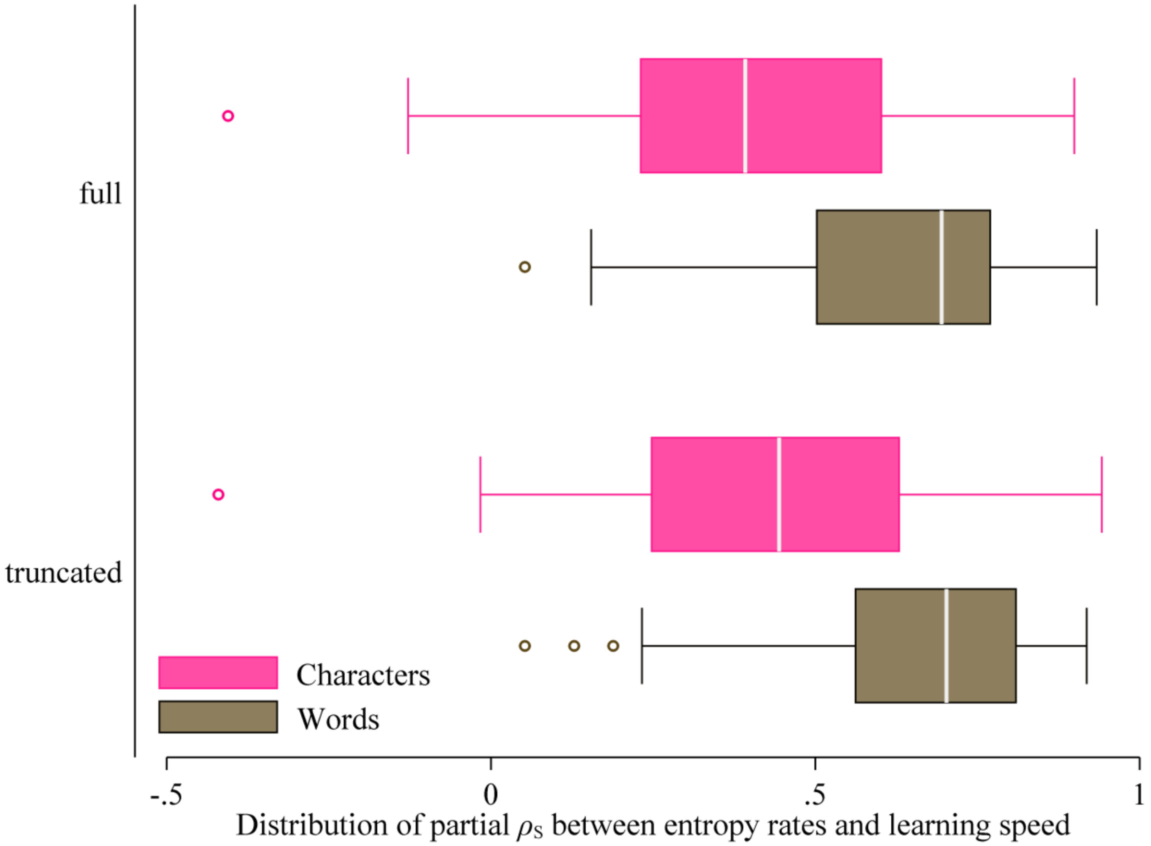

Supplementary Figure 23 | Relationship between *h* and *b*. For each corpus, on both symbolic levels (characters/words) and for both the full and the truncated variant (see section S4), we calculated the partial Spearman correlation[26] between *h* and *b*, after removing the effect of the message length *N*. Since lower *b*-values are indicative of slower convergence, a positive correlation between *h* and *b* supports the idea that documents that are harder to predict (i.e. higher *h*) are easier to learn to predict (i.e. higher *b*).

[26] Partial Spearman correlations are computed by first ranking *b*, *h* and *N*. The ranked variables are then used to calculate partial Pearson correlations (Greene 2003: 37).

By correlating b, i.e. learning difficulty, with h, i.e. predictability per corpus (partialling out the influence of the text length N), we arrive at a rather unexpected conclusion (Supplementary Figure 23): languages that are harder to predict tend to be easier/faster to learn for PPM ($\rho_{\text{med}} = 0.392$ for characters and $\rho_{\text{med}} = 0.694$ for words). Given statistical theories of language learnability (Gibson et al. 2019; Chater & Vitányi 2007; Chater & Vitányi 2003), we would have expected the opposite. To further evaluate the positive correlation between the entropy rate h and the learning difficulty b (cf. Supplementary Figure 3), we additionally calculated the compression rate r for the first half of each document t, i.e. for a document that is split into $\lfloor L/m \rfloor$ chunks, we calculate r at $\lfloor \lfloor L/m \rfloor / 2 \rfloor$ and call the resulting quantity r_{Q2}. Correspondingly, let r_{full} denote the compression rate at $\lfloor L/m \rfloor$.[27] For each t, we then computed the ratio between those two quantities, i.e. $₦_{Q2} = r_{full}/r_{Q2}$. $₦_{Q2}$ can be seen as a descriptive measure of learning speed where higher values are indicative of faster learning: for example, a value of $₦_{Q2} \approx .5$ implies that the second half of the text improves *PPM*'s ability to predict by a factor of ≈ 2, whereas a value of $₦_{Q2} \approx .66$ implies that *PPM*'s prediction accuracy is only improved by a factor of ≈ 1.5. If the second half of the text does not improve *PPM*'s ability to predict *at all*, $r_{full} \approx r_{Q2}$ and thus $₦_{Q2} \approx 1$. In addition, we calculated r at both the first (≈ 25% of t) and the third quartile (≈ 75% of t) and call the resulting quantities $₦_{Q1}$ and $₦_{Q3}$.

If the results presented in Figure 2b hold, we expect to observe a positive statistical association between h and both $₦_{Q1}$, $₦_{Q2}$ and $₦_{Q3}$, because for documents that are *harder* to *predict* in the limit (higher h), learning should be faster, i.e., after *PPM* has been trained on part of the input, it is already able to make predictions with accuracy more similar to the prediction accuracy of *PPM* trained on the full input (higher ₦). Supplementary Figure 24 shows that this is indeed the case. Consistently, the correlation strength tends to become weaker as the amount of training data increases (e.g. correlations tend to be weaker for $₦_{Q3}$ compared to $₦_{Q1}$) as learning speed is most evident in the initial part of the document (see, e.g., Figure 1c, Figure 1d, Supplementary Figure 3 – Supplementary Figure 21).

[27] In the main part of the paper, this quantity is denoted as just r.

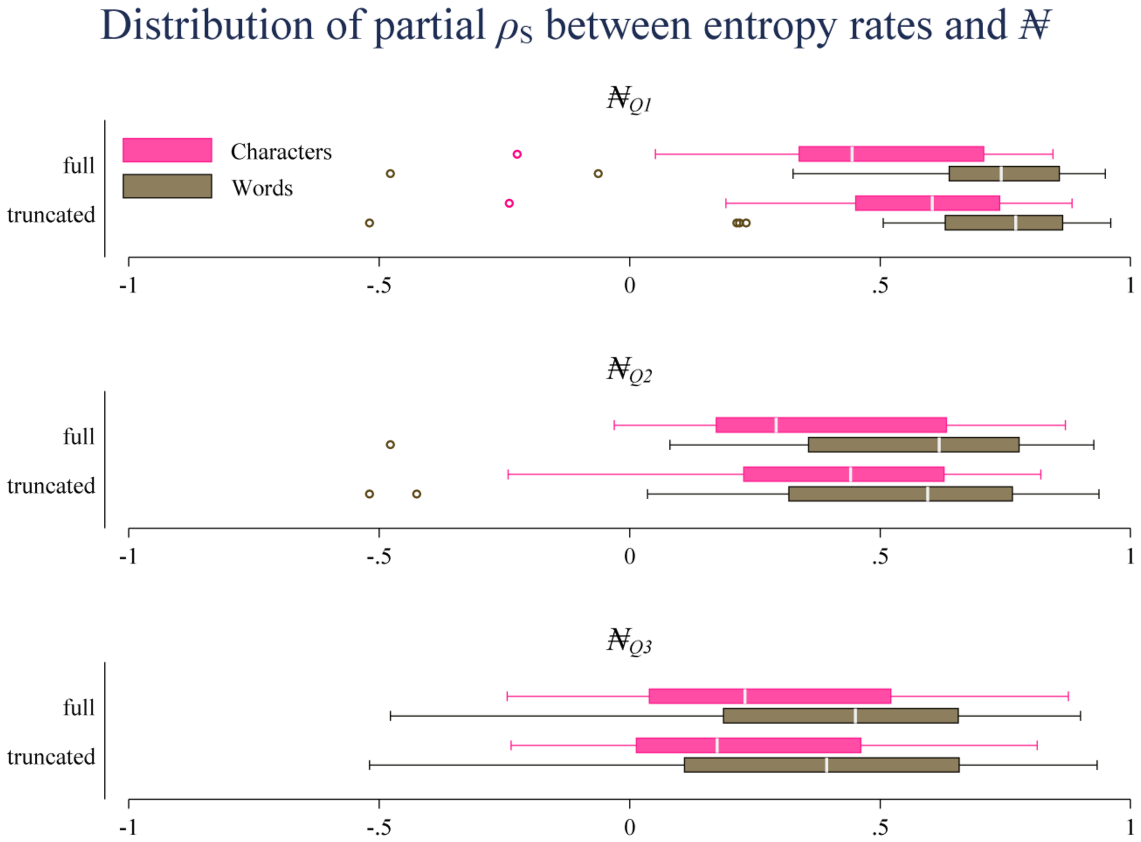

Supplementary Figure 24 | Relationship between *h* and ₦. For each corpus, on both symbolic levels (characters/words) and for both the full and the truncated variant (see section S4), we calculated the partial Spearman correlation between $₦_{Q1}$, $₦_{Q2}$ and $₦_{Q3}$ (described above) and the entropy rate *h*, after removing the effect of the message length. Since, as written above, higher values for ₦ are indicative of faster convergence, a positive correlation between *h* and ₦ supports the idea that documents that are harder to predict (i.e. higher *h*) are faster to learn to predict (i.e. higher ₦).

S9. Evaluating the similarity of complexity variables

Supplementary Table 6: Evaluation of the similarity of complexity variables. 1st column: Method, Spearman vs LMM as described in the main part of the paper. 2nd column: version, full vs. truncated variant (see section S4). 3rd column: variables that are correlated with each other, as described in the main part of the paper. 4th column: Variant, All – all corpora are included, Latin – only documents that use Latin Script are included, Parallel – only fully parallel corpora are included. 5th column: Symbol type, Words vs Characters. 6th column: Percentage of coefficients that are above zero. 7th column: Percentage of coefficients that are above the random baseline. 8th column: Average correlation coefficient. 9th column: Median correlation coefficient. 9th column: First quartile, i.e. 75% of the correlation coefficients are above Q_1. 10th column: Number of cases.

Method	Version	Variables	Variant	Symbol	P_0	P_R	ρ_{mean}	ρ_{med}	Q_1	N_ρ
Spearman	full	[h(κ),h(ι)]	All	Words	96.07%	92.80%	0.67	0.74	0.57	764
		[h(κ),h(ι)]	All	Characters	96.86%	79.97%	0.55	0.59	0.35	764
		[h(κ),h(ι)]	All	Across	92.93%	56.74%	0.38	0.37	0.20	1,528
		[h(κ),h(ι)]	Latin	Words	96.35%	91.22%	0.67	0.74	0.59	740
		[h(κ),h(ι)]	Latin	Characters	92.70%	64.32%	0.46	0.50	0.24	740
		[h(κ),h(ι)]	Latin	Across	93.78%	66.28%	0.43	0.48	0.26	1,480
		[h(κ),h(ι)]	Parallel	Words	95.12%	90.62%	0.69	0.78	0.60	512
		[h(κ),h(ι)]	Parallel	Characters	98.05%	82.42%	0.61	0.68	0.45	512
		[h(κ),h(ι)]	Parallel	Across	94.24%	64.26%	0.44	0.46	0.28	1,024
		[h(κ),H_Crúbadán]	All	Words	97.50%	95.00%	0.54	0.51	0.42	40
		[h(κ),H_Crúbadán]	All	Across	97.50%	45.00%	0.36	0.30	0.20	40
		[h(κ),H_Crúbadán]	Latin	Words	100.00%	92.31%	0.69	0.75	0.64	39
		[h(κ),H_Crúbadán]	Latin	Across	92.31%	79.49%	0.42	0.46	0.27	39
		[r(κ),r(ι)]	All	Words	100.00%	97.38%	0.78	0.82	0.73	764
		[r(κ),r(ι)]	All	Characters	100.00%	99.74%	0.80	0.82	0.73	764
		[r(κ),r(ι)]	All	Across	98.82%	82.20%	0.50	0.51	0.37	1,528
		[r(κ),r(ι)]	Latin	Words	100.00%	99.19%	0.78	0.82	0.71	740
		[r(κ),r(ι)]	Latin	Characters	99.46%	94.46%	0.70	0.74	0.61	740
		[r(κ),r(ι)]	Latin	Across	99.73%	88.04%	0.57	0.60	0.47	1,480
		[r(κ),r(ι)]	Parallel	Words	100.00%	97.27%	0.79	0.83	0.73	512
		[r(κ),r(ι)]	Parallel	Characters	100.00%	99.61%	0.81	0.83	0.74	512
		[r(κ),r(ι)]	Parallel	Across	99.02%	86.33%	0.54	0.56	0.41	1,024
		[H(κ),H(ι)]	All	Words	99.63%	94.53%	0.69	0.73	0.59	804
		[H(κ),H(ι)]	All	Characters	100.00%	100.00%	0.92	0.94	0.90	741
		[H(κ),H(ι)]	All	Across	97.34%	73.38%	0.39	0.40	0.29	1,544
		[H(κ),H(ι)]	Latin	Words	99.74%	94.22%	0.73	0.79	0.65	779
		[H(κ),H(ι)]	Latin	Characters	100.00%	99.86%	0.89	0.92	0.85	740
		[H(κ),H(ι)]	Latin	Across	97.96%	57.01%	0.38	0.38	0.25	1,519
		[H(κ),H(ι)]	Parallel	Words	100.00%	98.24%	0.73	0.76	0.65	512
		[H(κ),H(ι)]	Parallel	Characters	100.00%	100.00%	0.93	0.95	0.90	496
		[H(κ),H(ι)]	Parallel	Across	98.51%	74.50%	0.41	0.41	0.31	1,008
	truncated	[h(κ),h(ι)]	All	Words	94.90%	92.15%	0.63	0.69	0.54	764

		[h(κ),h(ι)]	All	Characters	94.76%	73.82%	0.48	0.50	0.30	764
		[h(κ),h(ι)]	All	Across	90.90%	54.84%	0.34	0.35	0.17	1,528
		[h(κ),h(ι)]	Latin	Words	94.73%	90.54%	0.63	0.70	0.55	740
		[h(κ),h(ι)]	Latin	Characters	87.30%	51.76%	0.36	0.37	0.16	740
		[h(κ),h(ι)]	Latin	Across	89.59%	60.27%	0.38	0.42	0.23	1,480
		[h(κ),h(ι)]	Parallel	Words	93.75%	91.02%	0.66	0.75	0.60	512
		[h(κ),h(ι)]	Parallel	Characters	97.07%	78.71%	0.53	0.57	0.37	512
		[h(κ),h(ι)]	Parallel	Across	93.07%	64.26%	0.40	0.41	0.24	1,024
		[h(κ),H_Crúbadán]	All	Words	97.50%	97.50%	0.52	0.50	0.41	40
		[h(κ),H_Crúbadán]	All	Across	97.50%	80.00%	0.34	0.34	0.19	40
		[h(κ),H_Crúbadán]	Latin	Words	97.44%	97.44%	0.67	0.71	0.62	39
		[h(κ),H_Crúbadán]	Latin	Across	94.87%	69.23%	0.39	0.37	0.18	39
		[r(κ),r(ι)]	All	Words	99.87%	99.35%	0.76	0.80	0.69	764
		[r(κ),r(ι)]	All	Characters	100.00%	100.00%	0.81	0.83	0.73	764
		[r(κ),r(ι)]	All	Across	98.76%	79.32%	0.50	0.52	0.36	1,528
		[r(κ),r(ι)]	Latin	Words	99.73%	98.78%	0.77	0.80	0.70	740
		[r(κ),r(ι)]	Latin	Characters	99.86%	95.00%	0.71	0.75	0.63	740
		[r(κ),r(ι)]	Latin	Across	99.32%	91.55%	0.59	0.60	0.49	1,480
		[r(κ),r(ι)]	Parallel	Words	100.00%	99.41%	0.79	0.82	0.72	512
		[r(κ),r(ι)]	Parallel	Characters	100.00%	100.00%	0.82	0.85	0.76	512
		[r(κ),r(ι)]	Parallel	Across	99.22%	83.89%	0.55	0.57	0.42	1,024
		[H(κ),H(ι)]	All	Words	99.87%	97.69%	0.70	0.72	0.59	780
		[H(κ),H(ι)]	All	Characters	100.00%	100.00%	0.92	0.93	0.90	741
		[H(κ),H(ι)]	All	Across	96.71%	71.20%	0.36	0.38	0.27	1,521
		[H(κ),H(ι)]	Latin	Words	99.74%	95.25%	0.73	0.77	0.63	779
		[H(κ),H(ι)]	Latin	Characters	100.00%	100.00%	0.89	0.91	0.85	740
		[H(κ),H(ι)]	Latin	Across	96.31%	56.29%	0.35	0.36	0.23	1,519
		[H(κ),H(ι)]	Parallel	Words	99.80%	98.59%	0.72	0.75	0.64	496
		[H(κ),H(ι)]	Parallel	Characters	100.00%	100.00%	0.93	0.94	0.90	496
		[H(κ),H(ι)]	Parallel	Across	97.38%	65.62%	0.37	0.37	0.27	992
ρ_{LMM}	full	[h(κ),h(ι)]	All	Words	95.95%	91.09%	0.60	0.66	0.45	1,482
		[h(κ),h(ι)]		Characters	96.90%	81.98%	0.52	0.56	0.30	1,482
		[h(κ),h(ι)]		Across	91.29%	61.14%	0.30	0.27	0.13	3,042
		[h(κ),H_Crúbadán]		Words	97.44%	82.05%	0.36	0.35	0.19	39
		[h(κ),H_Crúbadán]		Across	94.87%	41.03%	0.23	0.14	0.06	39
		[r(κ),r(ι)]		Words	100.00%	99.46%	0.72	0.77	0.62	1,482
		[r(κ),r(ι)]		Characters	99.93%	99.66%	0.79	0.85	0.70	1,482
		[r(κ),r(ι)]		Across	98.59%	74.95%	0.37	0.36	0.19	3,042
		[H(κ),H(ι)]		Words	99.23%	92.82%	0.58	0.61	0.44	1,560
		[H(κ),H(ι)]		Characters	100.00%	100.00%	0.93	0.97	0.91	1,482
		[H(κ),H(ι)]		Across	94.65%	59.29%	0.28	0.27	0.13	3,120

S10. Overview of the Linear Mixed-effects models

Supplementary Table 7: Results of LMMs that use speaker population size (logged) as fixed effect and (crossed) random intercepts for language, language family, macro-area, country, writing system and corpus. 1st column: outcome variable (standardized per corpus). 2nd column: version, full vs. truncated variant (see section S4). 3rd column: symbol type, W – words, C – characters. 4th column: Included corpora, all vs. fully parallel only. Each row lists the model with the lowest AIC for the variables specified in column 1 – 4. 5th column: difference in AIC, i.e. $AIC_r - AIC_f$, where AIC_f refers to the model with the lowest AIC where a fixed effect for speaker population size is included, whereas AIC_r refers to the model with the lowest AIC where no fixed effect for speaker population size included. Note that in models without a fixed effect for speaker population size, we also exclude potential random slopes. 6th column: estimated β_1. 7th column: z-statistic, i.e. $\hat{\beta}_1/\hat{\sigma}_{\hat{\beta}_1}$. 8th column: p-value based on two-sided parametric significance tests. 9th column: number of cases. 10th column: random slope specification. Note that all models contain random effects for corpus, macro-area, script, country, family and language except for the model with $H_{Crubadan}$ as the outcome that does not contain a random effect for corpus, but additional fixed effects for text length, available number of documents (both logged) and a binary variable indicating if the word frequency list is truncated (no/yes; see Methods: Differences across populations and SI: S.3.8 for details).

Outcome	Version	Corpora	Symbol	ΔAIC	$\hat{\beta}_1$	z	p_P	N	Random slopes
h	full	all	W	30.709	0.054	4.071	0.000	3853	corpus country family
h	full	parallel	W	33.272	0.044	2.902	0.004	3224	corpus script country family
h	truncated	all	W	44.405	0.062	4.728	0.000	3853	corpus macroarea country family
h	truncated	parallel	W	44.353	0.053	3.622	0.000	3224	corpus country family
h	full	all	C	42.561	0.058	2.127	0.033	3853	corpus script family
h	full	parallel	C	36.750	0.050	1.823	0.068	3224	corpus script country family
h	truncated	all	C	54.462	0.060	2.297	0.022	3853	corpus macroarea script country family
h	truncated	parallel	C	46.423	0.050	2.256	0.024	3224	corpus macroarea script country family
H	full	all	W	46.573	0.066	4.269	0.000	3853	corpus macroarea family
H	full	parallel	W	37.911	0.064	4.344	0.000	3224	corpus macroarea family
H	truncated	all	W	47.301	0.066	3.910	0.000	3849	corpus macroarea country family
H	truncated	parallel	W	41.018	0.065	3.862	0.000	3220	corpus macroarea family
H	full	all	C	54.135	0.011	1.047	0.295	3849	corpus macroarea country family
H	full	parallel	C	42.168	0.012	1.068	0.285	3220	corpus macroarea country family
H	truncated	all	C	53.939	0.010	1.006	0.314	3849	corpus macroarea country family
H	truncated	parallel	C	42.068	0.012	1.042	0.297	3220	corpus macroarea country family
$H_{Crubadan}$	full	all	W	130.648	0.160	5.441	0.000	1914	macroarea script country family
r	full	all	W	46.432	0.067	5.319	0.000	3853	macroarea country family
r	full	parallel	W	42.397	0.062	4.437	0.000	3224	corpus country family
r	truncated	all	W	58.042	0.070	4.444	0.000	3853	corpus macroarea script family
r	truncated	parallel	W	54.138	0.064	4.102	0.000	3224	corpus macroarea family
r	full	all	C	213.467	0.066	2.472	0.013	3853	corpus macroarea script country family
r	full	parallel	C	201.967	0.050	1.776	0.076	3224	corpus macroarea script country family
r	truncated	all	C	189.179	0.071	2.718	0.007	3853	corpus macroarea script country family
r	truncated	parallel	C	176.588	0.052	1.912	0.056	3224	corpus macroarea script country family

S11. Testing different compression algorithms

Supplementary Table 8 | Evaluating three different compression algorithms. 1st column: symbol type. 2nd column: document name (corpus base: EUconst, cf. S3.5.2). 3rd – 5th column: compressed length *R* in bytes for *PPMd*, *PAQ8l* and *CMIX*. 7th – 8th column: time *T* needed for compression in seconds for *PPMd*, *PAQ8l* and *CMIX*. Values in parentheses show the percentage ratio of the value to the corresponding *PPMd* value, e.g. R_{PAQ8l}/ R_{PPMd} | Values are rounded for illustration purpose only.

	translation	R_{PPMd}	R_{PAQ8l}	R_{CMIX}	T_{PPMd}	T_{PAQ8l}	T_{CMIX}
Characters	cs	97,889 (100%)	93,812 (96%)	88,810 (91%)	0.56 (100%)	55.65 (9,867%)	2,417.27 (428,595%)
	da	91,485 (100%)	87,881 (96%)	83,103 (91%)	0.58 (100%)	62.03 (10,732%)	2,402.39 (415,638%)
	de	107,288 (100%)	102,820 (96%)	96,541 (90%)	0.45 (100%)	72.98 (16,111%)	2,971.08 (655,867%)
	el	110,621 (100%)	102,887 (93%)	94,513 (85%)	0.80 (100%)	111.30 (13,929%)	5,658.78 (708,233%)
	en	92,920 (100%)	88,594 (95%)	83,669 (90%)	0.45 (100%)	63.14 (13,938%)	2,470.39 (545,340%)
	es	96,594 (100%)	92,113 (95%)	86,075 (89%)	0.47 (100%)	85.55 (18,279%)	3,297.09 (704,507%)
	et	96,832 (100%)	91,992 (95%)	87,479 (90%)	0.34 (100%)	53.95 (15,684%)	2,338.24 (679,719%)
	fi	99,180 (100%)	94,056 (95%)	89,210 (90%)	0.58 (100%)	75.92 (13,135%)	2,643.03 (457,272%)
	fr	94,596 (100%)	89,863 (95%)	84,156 (89%)	0.56 (100%)	62.03 (11,018%)	2,956.86 (525,197%)
	ga	101,003 (100%)	96,610 (96%)	91,022 (90%)	0.45 (100%)	72.97 (16,108%)	2,718.98 (600,217%)
	hu	110,552 (100%)	105,105 (95%)	99,183 (90%)	0.58 (100%)	81.08 (14,027%)	3,410.19 (589,998%)
	it	95,917 (100%)	91,083 (95%)	85,772 (89%)	0.45 (100%)	62.36 (13,766%)	2,904.77 (641,229%)
	lt	97,239 (100%)	92,780 (95%)	87,805 (90%)	0.56 (100%)	60.06 (10,668%)	2,663.88 (473,158%)
	lv	94,495 (100%)	89,866 (95%)	85,050 (90%)	0.58 (100%)	50.44 (8,726%)	2,210.50 (382,439%)
	mt	97,260 (100%)	92,173 (95%)	86,769 (89%)	0.58 (100%)	61.17 (10,583%)	2,994.38 (518,058%)
	nl	100,079 (100%)	95,825 (96%)	90,704 (91%)	0.56 (100%)	65.97 (11,717%)	2,857.31 (507,515%)
	pl	102,597 (100%)	98,132 (96%)	92,411 (90%)	0.56 (100%)	61.94 (11,001%)	2,412.88 (428,575%)
	pt	83,482 (100%)	80,058 (96%)	75,087 (90%)	0.45 (100%)	48.36 (10,675%)	2,070.78 (457,126%)
	sk	100,880 (100%)	96,708 (96%)	91,288 (90%)	0.56 (100%)	57.77 (10,279%)	2,254.45 (401,148%)
	sl	104,722 (100%)	100,175 (96%)	94,067 (90%)	0.58 (100%)	53.19 (9,202%)	2,775.20 (480,139%)
	sv	96,634 (100%)	92,490 (96%)	87,785 (91%)	0.58 (100%)	60.84 (10,508%)	3,228.81 (557,653%)
Words	cs	100,953 (100%)	92,526 (92%)	89,724 (89%)	0.48 (100%)	32.54 (6,723%)	1,405.97 (290,491%)
	da	92,555 (100%)	85,186 (92%)	82,340 (89%)	0.56 (100%)	41.23 (7,362%)	1,471.57 (262,780%)
	de	108,209 (100%)	99,787 (92%)	96,422 (89%)	0.45 (100%)	43.89 (9,689%)	1,736.75 (383,389%)
	el	107,355 (100%)	98,182 (91%)	94,910 (88%)	0.45 (100%)	43.11 (9,516%)	1,721.97 (380,125%)
	en	95,103 (100%)	88,137 (93%)	84,635 (89%)	0.45 (100%)	45.95 (10,122%)	1,749.44 (385,339%)
	es	99,833 (100%)	92,069 (92%)	88,456 (89%)	0.34 (100%)	44.53 (12,945%)	1,927.91 (560,438%)
	et	100,516 (100%)	91,432 (91%)	88,943 (88%)	0.36 (100%)	35.45 (9,848%)	1,387.56 (385,434%)
	fi	101,887 (100%)	92,257 (91%)	89,819 (88%)	0.34 (100%)	31.41 (9,130%)	1,163.80 (338,313%)
	fr	98,342 (100%)	90,502 (92%)	87,071 (89%)	0.45 (100%)	43.55 (9,613%)	1,877.67 (414,497%)
	ga	105,001 (100%)	96,953 (92%)	93,323 (89%)	0.34 (100%)	44.42 (12,913%)	2,082.86 (605,482%)
	hu	116,367 (100%)	106,342 (91%)	102,902 (88%)	0.45 (100%)	44.22 (9,761%)	1,785.98 (394,257%)
	it	101,032 (100%)	92,921 (92%)	89,628 (89%)	0.34 (100%)	37.09 (10,783%)	1,743.98 (506,972%)
	lt	102,814 (100%)	93,612 (91%)	90,961 (88%)	0.36 (100%)	37.42 (10,424%)	1,343.33 (374,187%)
	lv	99,626 (100%)	91,306 (92%)	88,409 (89%)	0.45 (100%)	41.36 (9,130%)	1,501.20 (331,391%)
	mt	103,441 (100%)	94,928 (92%)	91,867 (89%)	0.34 (100%)	37.76 (11,039%)	1,530.66 (447,563%)
	nl	100,515 (100%)	93,070 (93%)	89,459 (89%)	0.45 (100%)	47.81 (10,555%)	1,875.30 (413,973%)
	pl	105,732 (100%)	96,907 (92%)	93,853 (89%)	0.34 (100%)	43.88 (12,754%)	1,862.20 (541,338%)
	pt	85,521 (100%)	78,714 (92%)	75,903 (89%)	0.34 (100%)	33.16 (9,638%)	1,487.17 (432,318%)
	sk	104,137 (100%)	95,509 (92%)	92,478 (89%)	0.34 (100%)	39.17 (11,387%)	1,614.19 (469,240%)
	sl	110,066 (100%)	101,039 (92%)	97,793 (89%)	0.45 (100%)	42.56 (9,396%)	1,798.20 (396,955%)
	sv	97,810 (100%)	89,981 (92%)	86,971 (89%)	0.47 (100%)	41.05 (8,752%)	1,733.39 (369,593%)

We used the EUconst corpus (see S3.5.2) to evaluate the performance of two different compression algorithms in comparison to *7-zip PPMd*. We used two state-of-the-art algorithms, *PAQ8l* (Mahoney 2005) and *CMIX* v19 (Knoll 2021). The order of the *PPMd* model was determined as described above (see S3.1). For *PAQ8l*, option "-8" was selected to achieve the best compression level. For *CMIX*, option "n" was selected to compress without preprocessing. In addition, no dictionary was used for pretraining. Supplementary Table 8 shows that both *PAQ8l* and *CMIX* achieve better compression lengths than *PPMd* at the expense of being much slower: on average, compression lengths achieved by *PAQ8l* on the level of characters are 95.32% the compression lengths achieved by *PPMd* and 91.80% on the level of characters. For *CMIX*, corresponding quantities are 89.81% on the level of characters and 88.73% on the level of words. Compared to *PPMd*, *PAQ8l* needs – on average – 12,378.88% more computation time on the level of characters and 10,070.53% on the level of words to achieve this level of compression. For *CMIX*, corresponding quantities are 531,315.50% on the level of characters and 413,527.30% on the level of words.

It is worth pointing out that the Spearman correlations between all three compression lengths are very high on both symbolic levels (all $\rho_s > 0.979$). This supports the results presented in the main part of the paper (cf. Figure 4).

References

Aulamo, Mikko, Umut Sulubacak, Sami Virpioja & Jörg Tiedemann. 2020. OpusTools and Parallel Corpus Diagnostics. In *Proceedings of the 12th Language Resources and Evaluation Conference*, 3782–3789. Marseille, France: European Language Resources Association. https://www.aclweb.org/anthology/2020.lrec-1.467.

Baayen, R. Harald. 2001. *Word Frequency Distributions*. Dordrecht: Kluwer Academic Publishers.

Bentz, Christian, Dimitrios Alikaniotis, Michael Cysouw & Ramon Ferrer-i-Cancho. 2017. The Entropy of Words—Learnability and Expressivity across More than 1000 Languages. *Entropy* 19(6). 275. https://doi.org/10.3390/e19060275.

Bentz, Christian, Dan Dediu, Annemarie Verkerk & Gerhard Jäger. 2018. The evolution of language families is shaped by the environment beyond neutral drift. *Nature Human Behaviour* 2(11). 816–821. https://doi.org/10.1038/s41562-018-0457-6.

Chater, Nick & Paul Vitányi. 2003. Simplicity: a unifying principle in cognitive science? *TRENDS in Cognitive Science* 7(1). 19–22. https://doi.org/10.1016/S1364-6613(02)00005-0.

Chater, Nick & Paul Vitányi. 2007. 'Ideal learning' of natural language: Positive results about learning from positive evidence. *Journal of Mathematical Psychology* 51(3). 135–163. https://doi.org/10.1016/j.jmp.2006.10.002.

Chen, Stanley F. & Joshua Goodman. 1996. An Empirical Study of Smoothing Techniques for Language Modeling. In *34th Annual Meeting of the Association for Computational Linguistics*, 310–318. Santa Cruz, California, USA: Association for Computational Linguistics. https://doi.org/10.3115/981863.981904.

Cleary, J. & I. Witten. 1984. Data Compression Using Adaptive Coding and Partial String Matching. *IEEE Transactions on Communications* 32(4). 396–402. https://doi.org/10.1109/TCOM.1984.1096090.

Cysouw, Michael & Bernhard Wälchli. 2007. Parallel texts: using translational equivalents in linguistic typology. *Language Typology and Universals* 60(2). 95–99. https://doi.org/10.1524/stuf.2007.60.2.95.

Ebeling, W. & G. Nicolis. 1991. Entropy of Symbolic Sequences: The Role of Correlations. *Europhysics Letters (EPL)*. IOP Publishing 14(3). 191–196. https://doi.org/10.1209/0295-5075/14/3/001.

Gibson, Edward, Richard Futrell, Steven T. Piandadosi, Isabelle Dautriche, Kyle Mahowald, Leon Bergen & Roger Levy. 2019. How Efficiency Shapes Human Language. *TRENDS in Cognitive Science* 23(5). 389–407. https://doi.org/10.1016/j.tics.2019.02.003.

Goldhahn, Dirk, Thomas Eckart & Uwe Quasthoff. 2012. Building Large Monolingual Dictionaries at the Leipzig Corpora Collection: From 100 to 200 Languages. In *Proceedings of the Eighth International Conference on Language Resources and Evaluation (LREC'12)*, 759–765. Istanbul, Turkey: European Language Resources Association (ELRA). http://www.lrec-conf.org/proceedings/lrec2012/pdf/327_Paper.pdf.

Greene, William H. 2003. *Econometric analysis*. 5. ed., internat. ed. Upper Saddle River, NJ: Prentice-Hall.

Hammarström, Harald, Robert Forkel & Martin Haspelmath. 2019. *Glottolog 3.2*. Jena. https://glottolog.org/ accessed 2020-01-20.

Jaeger, T. Florian, Peter Graff, William Croft & Daniel Pontillo. 2011. Mixed effect models for genetic and areal dependencies in linguistic typology. *Linguistic Typology* 15(2). (27 June, 2018).

Knoll, Byron. 2021. CMIX. https://www.byronknoll.com/cmix.html. (3 February, 2022).

Knoll, Byron & Nando de Freitas. 2012. A Machine Learning Perspective on Predictive Coding with PAQ8. In *2012 Data Compression Conference*, 377–386. Snowbird, UT, USA: IEEE. https://doi.org/10.1109/DCC.2012.44.

Koplenig, Alexander. 2018. Stata tip 129: Efficiently processing textual data with Stata's new Unicode features. *Stata Journal* 18(1). 287–289.

Koplenig, Alexander. 2021. Quantifying the efficiency of written language. *Linguistics Vanguard* 7(s3). 20190057. https://doi.org/10.1515/lingvan-2019-0057.

Koplenig, Alexander, Marc Kupietz & Sascha Wolfer. 2022. Testing the Relationship between Word Length, Frequency, and Predictability Based on the German Reference Corpus. *Cognitive Science* 46(6). https://doi.org/10.1111/cogs.13090.

Koplenig, Alexander, Sascha Wolfer & Carolin Müller-Spitzer. 2019. Studying Lexical Dynamics and Language Change via Generalized Entropies: The Problem of Sample Size. *Entropy* 21(5). https://doi.org/10.3390/e21050464.

Levshina, Natalia. 2016. Verbs of letting in Germanic and Romance languages: A quantitative investigation based on a parallel corpus of film subtitles. *Languages in Contrast* 16(1). 84–117. https://doi.org/10.1075/lic.16.1.04lev.

Mahoney, M. 2005. Adaptive weighing of context models for lossless data compression. Florida Tech. http://hdl.handle.net/11141/154.

Mahoney, Matt. 2013. *Data Compression Explained*. Dell Inc. http://mattmahoney.net/dc/dce.html. (20 September, 2018).

Mayer, Thomas & Michael Cysouw. 2014. Creating a Massively Parallel Bible Corpus. In Nicoletta Calzolari (Conference Chair), Khalid Choukri, Thierry Declerck, Hrafn Loftsson, Bente Maegaard, Joseph Mariani, Asuncion Moreno, Jan Odijk & Stelios Piperidis (eds.), *Proceedings of the Ninth International Conference on Language Resources and Evaluation (LREC'14)*. Reykjavik, Iceland: European Language Resources Association (ELRA).

Montemurro, Marcelo A. & Damián H. Zanette. 2011. Universal Entropy of Word Ordering Across Linguistic Families. (Ed.) Michael Breakspear. *PLoS ONE* 6(5). e19875. https://doi.org/10.1371/journal.pone.0019875.

Moscoso del Prado Martín, Fermín. 2011. The mirage of morphological complexity. In *Proceedings of Quantitative Measures in Morphology and Morphological Development*. Center for Human Development, UC San Diego. http://csjarchive.cogsci.rpi.edu/proceedings/2011/papers/0836/paper0836.pdf.

Newport, Elissa L. 2016. Statistical language learning: computational, maturational, and linguistic constraints. *Language and Cognition* 8(03). 447–461. https://doi.org/10.1017/langcog.2016.20.

R Core Team. 2022. R: A Language and Environment for Statistical Computing. Vienna, Austria: R Foundation for Statistical Computing. https://www.R-project.org/.

Scannell, Kevin P. 2007. The Crúbadán Project: Corpus building for under-resourced languages. In *Proceedings of the 3rd Web as Corpus Workshop: Building and Exploring Web Corpora*, vol. 4, 5–15. http://cs.slu.edu/~scannell/pub/wac3.pdf.

Schürmann, Thomas & Peter Grassberger. 1996. Entropy estimation of symbol sequences. *Chaos: An Interdisciplinary Journal of Nonlinear Science* 6(3). 414. https://doi.org/10.1063/1.166191.
Simons, Gary F. & Charles D. Fennig. 2013. *Ethnologue: Languages of the World 17th edition*. Dallas, Texas: SIL International. http://www.ethnologue.com.
Simons, Gary F. & Charles D. Fennig. 2017. Ethnologue Global Dataset, Twentieth edition. https://www.ethnologue.com/sites/default/files/Ethnologue-20-Global%20Dataset%20Doc.pdf.
StataCorp. 2015. Stata Statistical Software. StataCorp: StataCorp.
StataCorp. 2023. Stata Statistical Software. StataCorp: StataCorp.
Takahira, Ryosuke, Kumiko Tanaka-Ishii & Łukasz Dębowski. 2016. Entropy Rate Estimates for Natural Language—A New Extrapolation of Compressed Large-Scale Corpora. *Entropy* 18(10). 364. https://doi.org/10.3390/e18100364.
Teahan, W. J., Yingying Wen, Rodger McNab & Ian H. Witten. 2000. A Compression-based Algorithm for Chinese Word Segmentation. *Computational Linguistics* 26(3). 375–393. https://doi.org/10.1162/089120100561746.
Teahan, William J. & David J. Harper. 2003. Using Compression-Based Language Models for Text Categorization. In W. Bruce Croft & John Lafferty (eds.), *Language Modeling for Information Retrieval*, 141–165. Dordrecht: Springer Netherlands. https://doi.org/10.1007/978-94-017-0171-6_7.
Tiedemann, Jörg. 2012. Parallel Data, Tools and Interfaces in OPUS. In *LREC'12 Proceedings*, 2214–2218. Istanbul, Turkey: ELRA.
Twain, Mark. 2004. *The Entire Project Gutenberg Works of Mark Twain*. https://www.gutenberg.org/ebooks/3200. (9 December, 2021).
Tweedie, Fiona J. & R. Harald Baayen. 1998. How Variable May a Constant be? Measures of Lexical Richness in Perspective. *Computers and the Humanities* 32(5). 323–352.
Unicode Consortium. 2019. Unicode Text Segmentation. *Unicode® Standard Annex #29*. http://www.unicode.org/reports/tr29/#Word_Boundaries. (23 July, 2019).
Ward, David J. & David J. C. MacKay. 2002. Fast hands-free writing by gaze direction: Artificial intelligence. *Nature* 418(6900). 838–838. https://doi.org/10.1038/418838a.
Weaver, Warren. 1949. Translation. In William N. Locke & A. Donald Boothe (eds.), *Machine Translation of Languages*, 15–23. Cambridge, MA: MIT Press.
Ziemski, Michał, Marcin Junczys-Dowmunt & Bruno Pouliquen. 2016. The United Nations Parallel Corpus v1.0. In *Proceedings of the Tenth International Conference on Language Resources and Evaluation (LREC'16)*, 3530–3534. Portorož, Slovenia: European Language Resources Association (ELRA). https://www.aclweb.org/anthology/L16-1561.
